# Customizable Manufacturing of Polyamide Membranes with Programmable Layers and High Permselectivity by Electrospray Printer

**DOI:** 10.1002/advs.202509127

**Published:** 2025-09-12

**Authors:** Xieyang Xu, Yingsong Liu, Huijun Yu, Chenshuo Wang, Pin Hou, Huijiao Wang, Yanjun Liu, Kai Zhang, Peidong Su, Chunrong Wang, Jianbing Wang

**Affiliations:** ^1^ School of Chemical and Environmental Engineering China University of Mining and Technology – Beijing Beijing 100083 P. R. China; ^2^ National Key Laboratory of Coal Fine Exploration and Intelligent Development China University of Mining and Technology – Beijing Beijing 100083 P. R. China

**Keywords:** customizable printing, permselectivity, polyamide membrane, polymerization mechanism, programmable electrospray printer

## Abstract

Customizable printing of polyamide (PA) layer is intriguing due to achievement of controllable morphology and programmable crosslinking structure, producing high‐quality PA membranes for wide use. However, the state‐of‐the‐art electrospray facilitated interfacial polymerization only achieved control of layer thickness due to its indiscriminate monomer deposition. It lacked 3D design with slicing to specify layer geometry and is far away from customizable printing without achieving programmable features. Herein, a customizable manufacturing approach is established for printing the PA layer with an original programmable electrospray 3D printer, a coupled polymerization method and a numerical prediction model. In this approach, the PA layer is sliced into several ultrathin films. These films are printed individually by flexible use of interfacial, homogeneous, and surficial polymerization with the aid of the numerical model. The printed PA layer has programmable crosslinking degree varying layer by layer, ultrathin thickness of ≈14 nm, nanopores with narrow distribution of ≈0.32 nm, and intrinsic smoothness with ≈4 nm roughness. This structure enables the membranes to achieve water permeance and NaCl rejection of 3.1 L·m^−^^2^·h^−1^·bar^−1^ and 99.0%, respectively, approaching the limit of permeability‐selectivity trade‐off. This work offers a universal platform for on‐demand fabrication of nanoscale membranes.

## Graphical abstract

A customizable manufacturing approach is established for polyamide (PA) membrane with an original programmable electrospray 3D printer, coupled polymerization method and a numerical prediction model. The printed PA layers has ultrathin thickness, programmable crosslinking degree, and smooth surface. The unique structure enables the PA membrane to achieve high water permeance and NaCl rejection of 3.1 L·m^−^^2^·h^−1^·bar^−1^ and 99.0%, respectively.

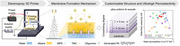

## 1 Introduction

3D printing (also known as additive manufacturing) represents an attractive prospect in the customizable printing of ceramics,^[^ ^1^, ^2^, ^3^ ^]^ metals,^[^ ^4^, ^5^ ^]^ polymers,^[^ ^6^, ^7^ ^]^ and biological organs.^[^ ^8^, ^9^ ^]^ Because of the limitation in resolution at a level of µm, traditional 3D printing techniques are usually insufficient for customizable printing of functional materials with nanostructure.^[^ ^10^, ^11^ ^]^ Pushing customizable printing into nanoscale level is intriguing as many functional materials have nanoscale structure. Polyamide (PA) membranes, one of typical functional materials, rely on nanoscale active layer to function in desalination,^[^ ^12^, ^13^ ^]^ lithium extraction^[^ ^14^, ^15^, ^16^ ^]^ and other separation processes.^[^ ^17^, ^18^, ^19^ ^]^ The performance of PA membranes is greatly determined by the features of active layer properties such as thickness and crosslinking degree.^[^ ^20^, ^21^, ^22^ ^]^ Fabrication of active layer with well‐designed features endows PA membranes with excellent performance including high permselectivity, strong antifouling and anti‐polarization ability.^[^ ^23^, ^24^, ^25^ ^]^ This is full of challenges due to the unique nanoscale structures and complex fabrication procedures of PA layer.

PA layer of reverse osmosis (RO)membranes is traditionally prepared via interfacial polymerization (IP).^[^ ^26^ ^]^ Once the reactants, for example, m‐phenylenediamine (MPD) in an aqueous phase and trimesoyl chloride (TMC) in an organic phase, are in contact at the aqueous/organic interface, a thin film is formed and permitted to grow.^[^ ^22^, ^27^, ^28^ ^]^ The growing film will block the diffusion of reactants across the interface, resulting in diffusion‐limited growth.^[^ ^29^, ^30^ ^]^ Finally, a depth‐heterogeneous PA layer is produced in a few seconds with uncontrollable thickness and crosslinking degree and rough ridge‐and‐valley nanostructures, which have a great effect on water permeability, selectivity, fouling and polarization propensity for separation processes.^[^ ^31^ ^]^ The rapid, uncontrollable and complex IP reactions confound mechanism understanding and pose great difficulties in regulating PA layer formation.^[^ ^32^ ^]^ This limits our ability to obtain excellent PA membranes with high water permeability and salt rejection.

Recently, Chowdhury et.al. presented an electrospray (ES) facilitated IP method for the preparation of smooth PA layers and achieved control of thickness at nanoscale level.^[^ ^33^ ^]^ They referred to the method as “3D printing”. It has excited the research of ES‐IP. However, literature pointed out the method is not real 3D printing due to the lack of 3D design with thin layer slicing to specify layer geometry.^[^ ^34^ ^]^ In addition, the reported electrospray apparatus provides indiscriminate layer‐by‐layer deposition to a drum collector and thus cannot achieve programmable features. Therefore, state‐of‐the‐art technology is still far away from customizable preparation of PA layer. The gaps between the present technology and customizable printing include approaches, slicing technology, 3D configuration, and numerical model.^[^ ^25^, ^34^ ^]^ Furthermore, if scaled‐up production is considered, a flat plate collector may be suitable.^[^ ^34^ ^]^ Other issues need to be addressed including prevention of acyl chloride groups hydrolysis and prohibition of film defects generation as these were believed to be inevitable for ES‐IP.^[^ ^35^ ^]^

In this work, we established a programmable manufacturing approach for customizable printing of PA layer with innovation of programmable electrospray 3D printer and numerical prediction model. The printer had a specially designed mixed printing head, flat plate collector and programmable control system. Based on the model, the printer could produce PA membranes with controllable thickness, intrinsic smoothness and permselectivity higher than commercial membranes. More importantly, a special PA membrane with programmable crosslinking degree was designed and manufactured, and its permselectivity approached the upper‐bound limit of the selectivity‐permeability trade‐off relationship.

## 2 Results and Discussion

### 2.1 Programmable Electrospray 3D Printer and Electrospray Stability

**Figure 1:**
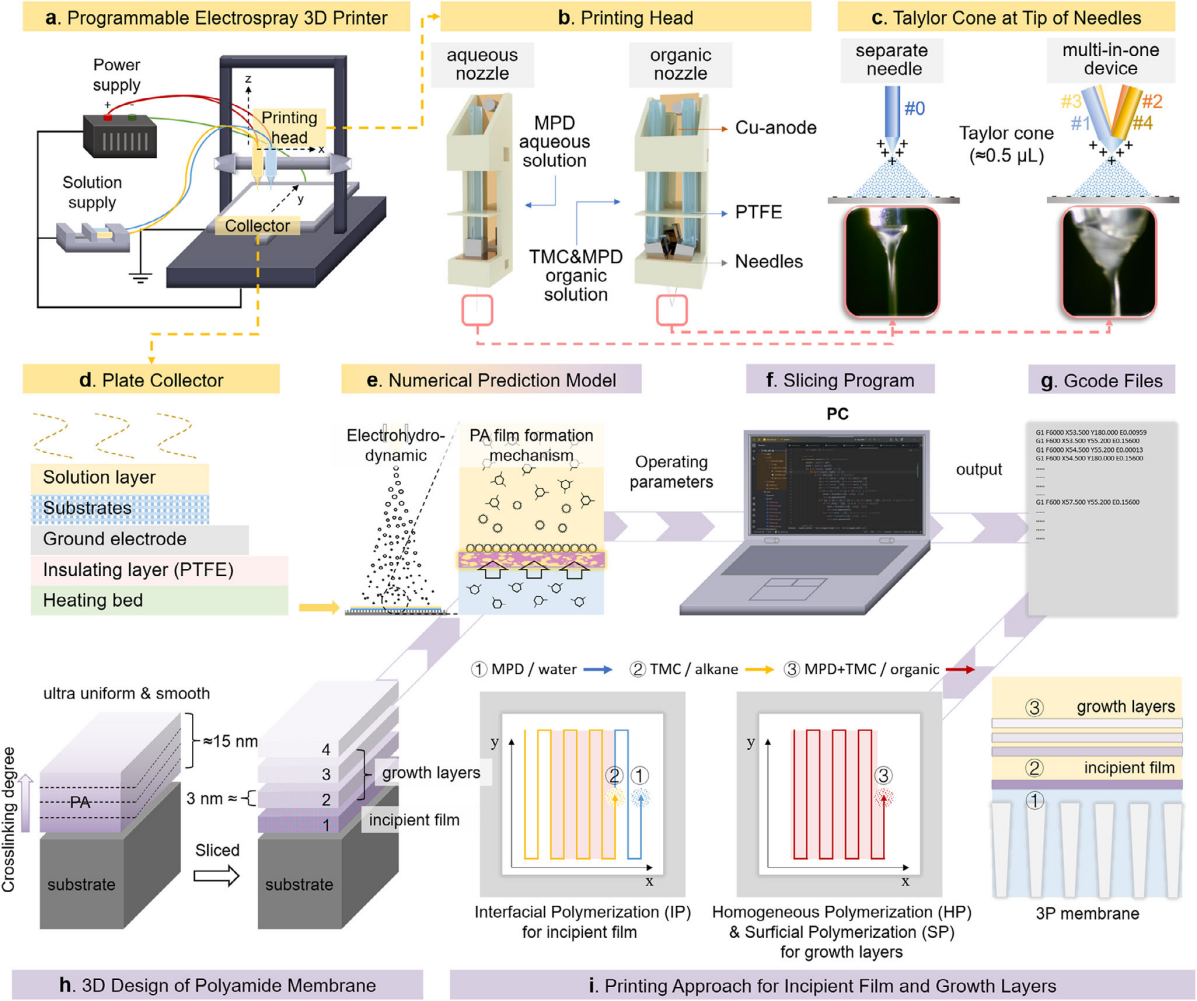
Programmable electrospray 3D printer and customizable manufacturing process of polyamide membranes. a) Programmable electrospray 3D printer (Aqueous nozzle, organic nozzle, flat plate collector, power supply, and solution supply). b) Aqueous nozzle (a separate needle) and organic nozzle (a multi‐in‐one device with 4 needles). c) Taylor cone‐jet spray ejected from separate needle and multi‐in‐one device. d) flat Plate collector. e) Numerical prediction model aiding programmable printing. f) Slicing program. g) Gcode files. h) 3D design of polyamide membrane. i) Schematic of printing approach shows the moving path of spraying needles for the 3P membranes (3P: IP for interfacial polymerization, HP for homogeneous polymerization, and SP for surficial polymerization, respectively).

We invented a programmable electrospray 3D printer (PE3DP) and coupled polymerization preparation method (**Figure** **1**; Note S2, Supporting Information) to realize customizable printing of PA layer. Notably, the PE3DP is specially endowed with a mixed printing head enabling versatile methods (Figure 1a–c), flat plate collector system capable of achieving 3D functionality (Figure 1a,d), numerical prediction model aiding printing operation (Figure 1e), programmable control system, slicing program (Figure 1f) and Gcode files (Figure 1g). The PE3DP sliced the PA layer into an incipient film and several growth layers using thin layer slicing technology (Figure 1h) and printed them via the flexible use of electrospray facilitated interfacial polymerization, homogeneous polymerization and surficial polymerization (ES‐IP‐HP‐SP or ES‐3P) (Figure 1i). As a result, the processes of the incipient film formation and diffusion‐limited layers growth were artificially separated and individually regulated, producing a smooth PA layer with high permselectivity, programmable crosslinking degree and controllable thickness. The PE3DP is operated by a microprocessor with Gcode files. To create Gcode files, the 3D design of the PA layer and printing parameters were written into Gcode files by the self‐developed slicing program (Figure 1f,g). The numerical prediction model was established based on polymerization reaction exploration and electrohydrodynamic analysis to aid the 3D design of the PA layer and determination of printing parameters (Note S3, Supporting Information).

To implement ES‐3P fabrication method, we designed a mixed printing head for the PE3DP, which is assembled by an aqueous nozzle and an organic nozzle (Figure 1b,c). The aqueous nozzle has a separate needle for spraying MPD aqueous solution (Needle #0, Video S1, Supporting Information). Organic nozzle comprises a multi‐in‐one device with four needles (Video S2, Supporting Information). These four needles extruded MPD/ethanol/n‐hexane solution (Needle #1), TMC/n‐hexane solution (Needle #2), n‐hexane liquid (Needle #3) and n‐decane liquid (Needle #4), respectively. Four needles sprayed out different solutions, and the four streams of the extruded solutions were then mixed into a Taylor cone. This design allows simultaneous deposition of MPD and TMC monomers in sprayed organic solutions onto the collector for undergoing polymerization reaction and enables continuous switching of solutions, which reduces the inhomogeneous deposition of monomers in horizontal direction (Video S2).

ES‐IP method was used for preparing the incipient film. An ultrafiltration (UF) membrane substrate was fixed on a flat plate. Three types of UF membrane were used in this study, which were polyether sulfone (PES), polysulfone (PSF) and polyacrylonitrile (PAN) (More details in Table S7, Supporting Information). Then, MPD aqueous solution (Needle #0) and TMC organic solution (Needle #2) were sprayed and deposited onto the surface of substrate (Figure S1, Supporting Information). The printing head traversed along the collector surface at the predetermined route and speeds (Figure 1i; Video S3, Supporting Information). After a single pass over the collector surface (referred to as single scan), an ultrathin incipient film was formed on the surface of the substrate.

The combination of ES‐HP and ES‐SP was used to prepare the growth layers. First, the organic nozzle extruded the solutions. The jets of the four organic solutions were mixed, and HP reactions occurred between MPD and TMC monomers in the mixed solution, producing oligomers with a crosslinking degree less than 50% (Figure S12, Supporting Information). Then, the mixed solution formed stable Taylor cone spray due to the design of the multi‐in‐one device, and the generated droplets were deposited onto the surface of the support. Finally, the oligomers in the droplets grew into a highly cross‐linked PA layer through SP. In this study, three growth layers were prepared, and one scan produced a layer. The resulting 4‐layer membranes were called 3P membranes. Monomer concentrations and organic solvent combinations for each layer printing were determined based on the calculation with the numerical model and we provided an example for their determination in Note S2.2 (Supporting Information). More details about membrane preparation are described in Note S2 (Supporting Information).

**Figure 2:**
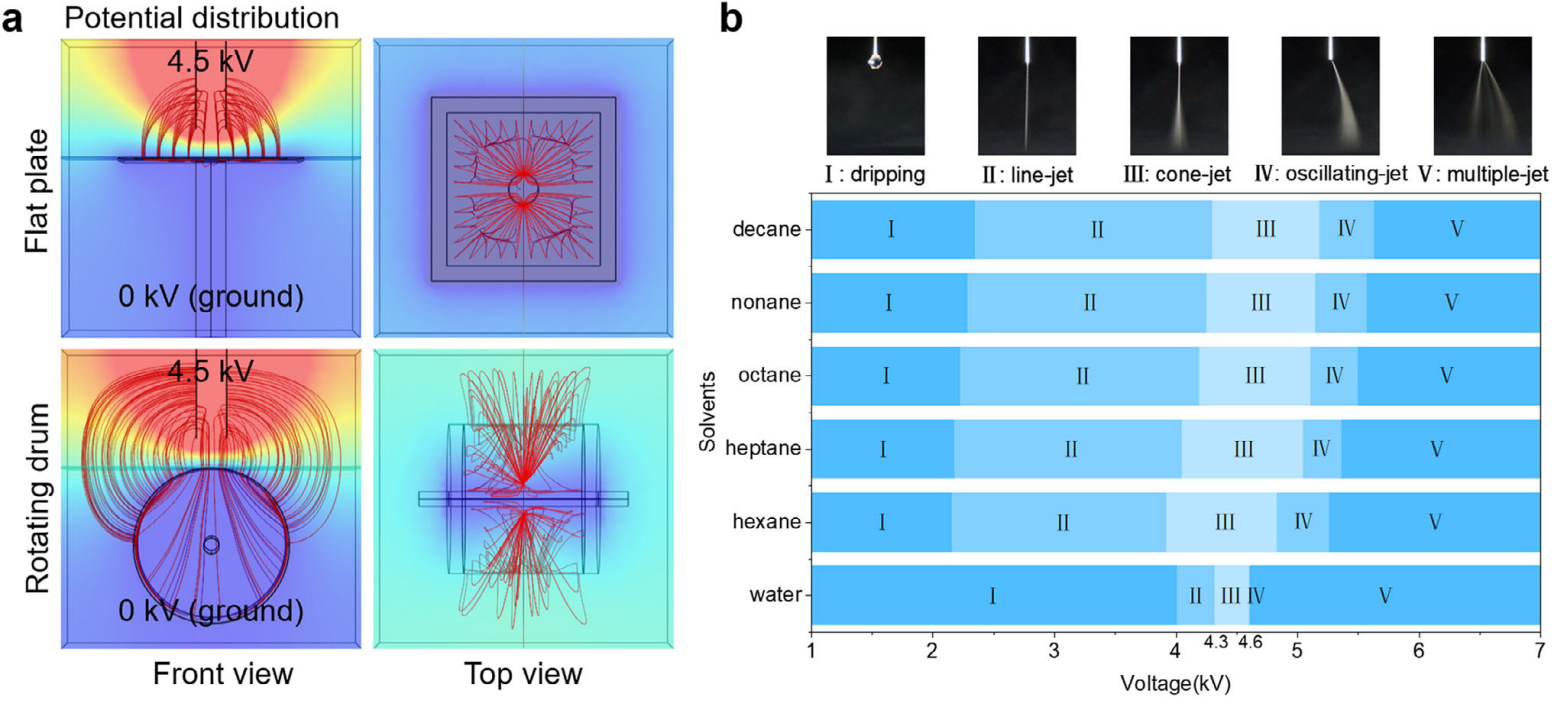
Comparison of potential distribution between various collectors and spray mode for programmable electrospray 3D printer. a) Potential distribution for the PE3DP using a flat plate collector (upper images, upper left for front view, and upper right for top view) and conventional ES‐IP equipment using a rotating drum collector (bottom images, bottom left for front view, and bottom right for top view). b) Evolution of spray mode with varying voltage for aqueous solution and different alkane solutions (I for dripping, II for line‐jet, III for cone‐jet, IV for oscillating‐jet, and V for multiple‐jet).

We configured the PE3DP with a flat plate collector system. Based on Cartesian kinematics, a control system and Gcode files were developed to coordinate the printing head and flat plate collector. The flat plate collector was operated at a relatively low speed on horizontal direction. Thus, the throwing out of the solution can be avoided, ensuring almost all the sprayed monomers deposited on the surface of the UF membrane substrate. This makes the PE3DP easy to control and scale up. The low potential region is evenly distributed on the surface of the flat plate collector so that the field strength is greatest in the perpendicular direction of needle (**Figure** **2**a). As a result, the sprays of both aqueous and organic solutions are kept exactly below the needles in Taylor cone‐jet mode (Figure S5, Supporting Information). This allows the microdroplets formed by the two ejected solutions to deposit on the same location of the collector. However, if a rotating drum is used, the power lines in the vicinity of low potential take on a spindle shape along its axial direction (Figure 2a). The electric field repulsion between the anode needles causes the microdroplets of two ejected solutions to fly in opposite directions, for which it is difficult to achieve uniform continuous coverage and polymerization of monomers. Additionally, the concentration of ionic liquid and applied voltage were carefully optimized many times, and suitable ionic liquid concentrations (Table S1, Supporting Information) and a narrow voltage window (4.3–4.6 kV) were ultimately found for both water and 5 types of alkanes. The conductivity values of aqueous solutions and organic solutions were about 20 and 2.5 µS cm^−1^, respectively. Finally, relatively stable Taylor cone‐jet sprays were obtained (Figure 2b; Video S4, Supporting Information), which is important for membrane production with uniform and reproducible structures and functions.

### 2.2 Formation Mechanisms of Incipient Film and Growth Layers

**Figure 3:**
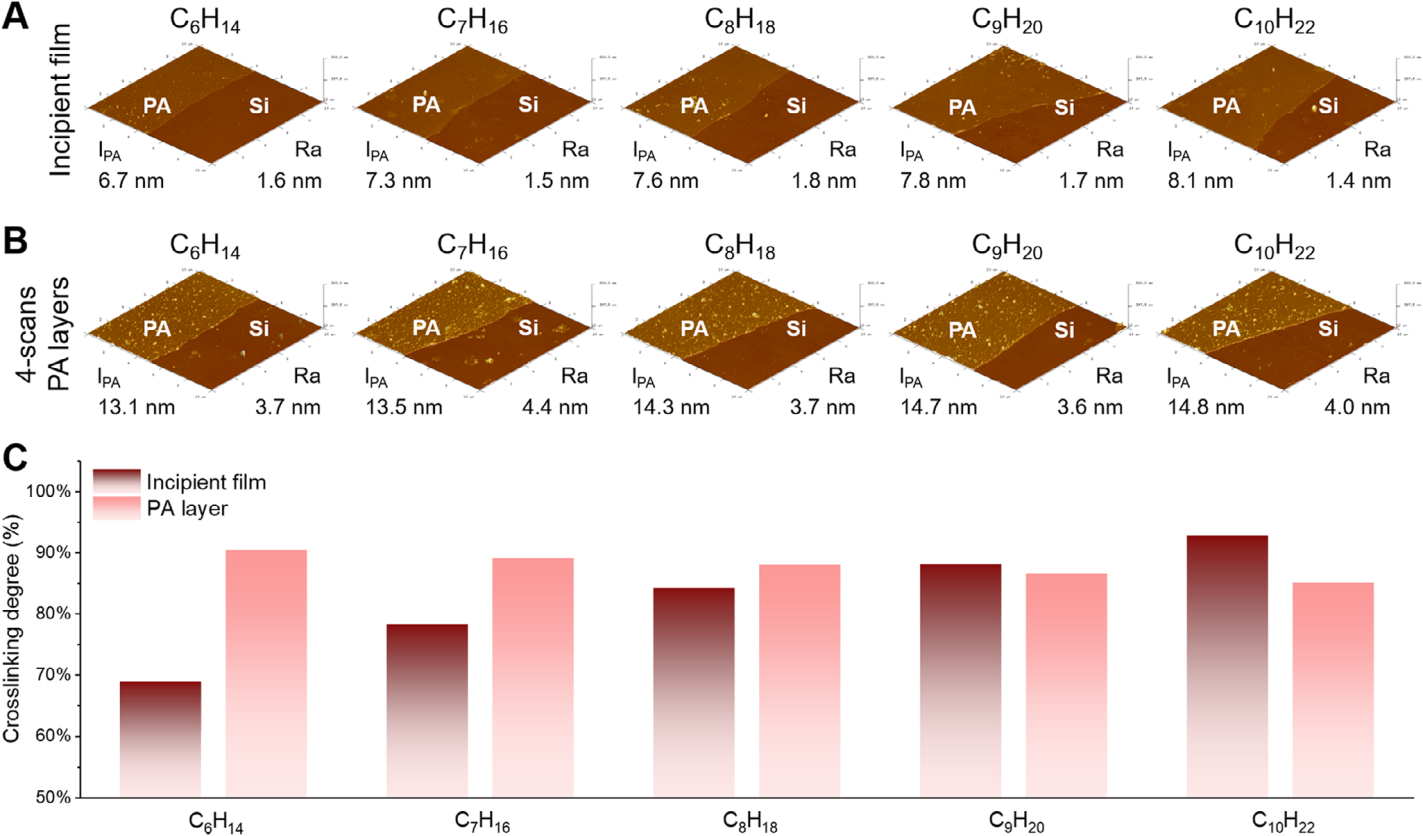
Analysis of AFM and XPS for the 3P membranes prepared by different alkane solvents. a) AFM analysis results of the incipient films (l_PA_ for film thickness and Ra for average roughness). b) AFM analysis results for the separation layer of various 3P membranes (l_PA_ for film thickness and Ra for average roughness). c) Crosslinking degrees of incipient film and PA layer for 3P membrane.

The developed PE3DP realized customizable printing of the incipient film and growth layer with diversified film formation mechanisms. We concluded the mechanism behind ES‐IP is attributed to the polymerization between MPD and TMC induced at the interface between two immiscible microdroplets. To verify the mechanism behind ES‐IP, we fabricated five incipient films by dissolving TMC monomers in five types of organic solvents (n‐hexane, n‐heptane, n‐octane, n‐nonane and n‐decane), respectively. These organic solvents have different volatilization rates. **Figure** **3** summarizes the results from the characterization of the incipient films with the analysis of scanning electron microscopy (SEM), atomic force microscopy (AFM) and X‐ray photoelectron spectroscopy (XPS).^[^ ^33^, ^36^ ^]^ The thicknesses of these incipient films are 6.9, 7.4, 7.6, 7.8 and 8.1 nm, respectively, and their crosslinking degrees are 69%, 78%, 84%, 88%, and 93%, respectively (Figure 3a,c). Both thickness and crosslinking degree increase with the decrease of the volatilization rate of organic solvents. This indicates polymerization time is correlated with volatilization rate of the used organic solvents. Thus, the above mechanism was confirmed and the residence time of organic solvent on the flat plate collector can be used as a critical parameter to regulate ES‐IP for customizable printing of the incipient film.

**Figure 4:**
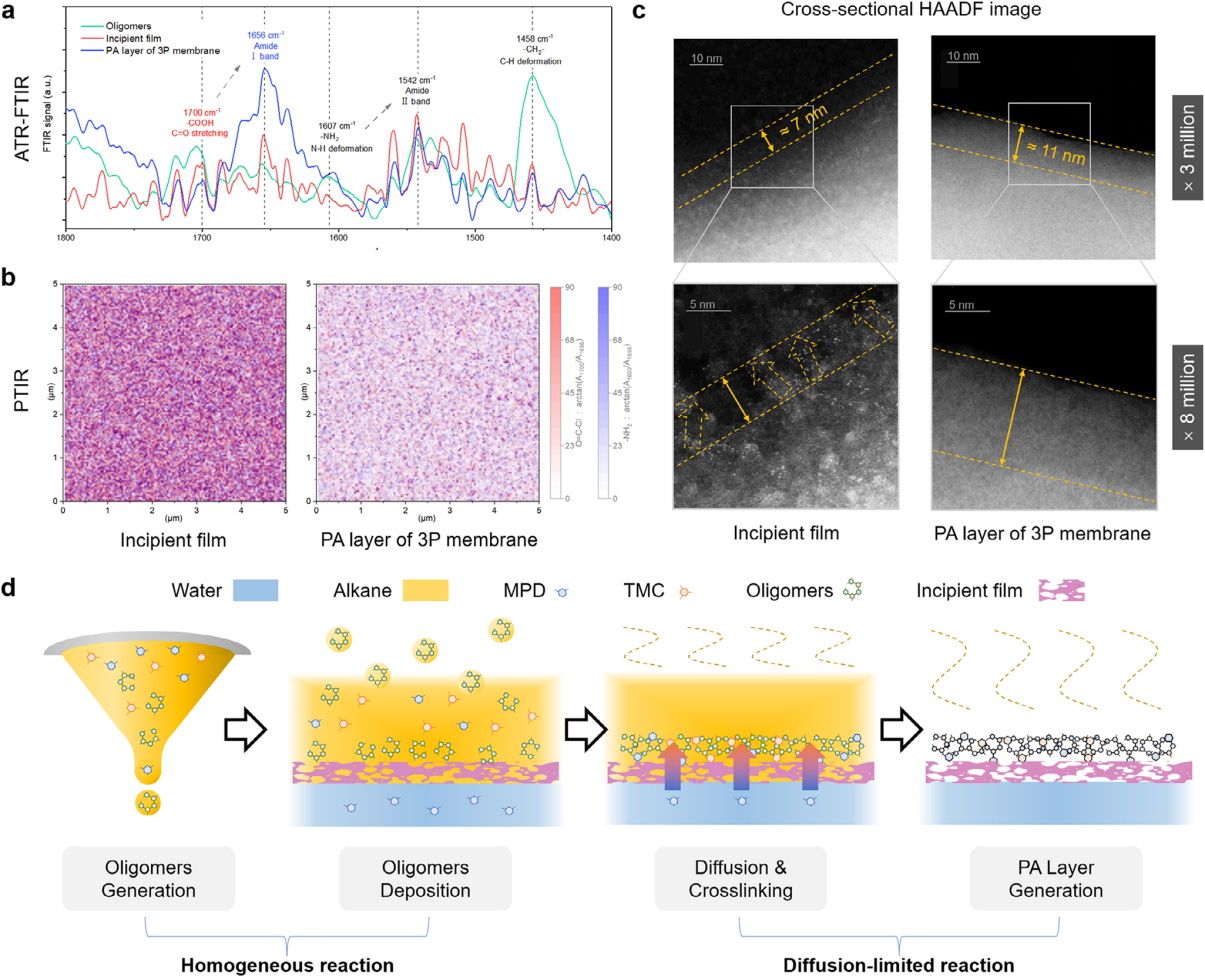
ATR‐FTIR, PTIR, and HAADF analysis for the 3P membrane. a) ATR‐FTIR analysis results of the oligomers, incipient film, and PA layer. The FTIR peaks at 1656, 1542, 1700, and 1600 cm^−1^ were assigned amide I band, amide II band, carboxyl groups, and amine groups, respectively. b) PTIR images of incipient film (left) and the PA layer of the 3P membrane (right). Red spots show the value of arctan(A_1700_/A_1656_) which represents the content of carboxyl group relative to amide bond on the formed polyamide matrix; Blue spots show the value of arctan(A_1600_/A_1656_) which represents the content of amine group relative to amide bond on the formed polyamide matrix. c) Cross‐sectional HAADF image of the incipient film (left) and PA layer (right) after impregnation of AgNO_3_ solution: ×3 million (above), ×8 million (below). d) Schematic for the mechanism of growth layers formation.

In comparison with the incipient film, the formation mechanism of the growth layers was completely different due to the ES‐HP and ES‐SP processes. The formation mechanism was elucidated using five 3P membranes which were prepared by the above incipient films, respectively. Each 3P membrane had three growth layers that were prepared with MPD alcohol/n‐decane solution (0.003%) and TMC n‐decane solution (0.005%). The measured crosslinking degrees of these 3P membranes are 90%, 88%, 87%, 86%, and 85%, respectively, as shown in the analysis of XPS (Figure 3c). They are significantly higher than the crosslinking degree of the oligomers (Figure S12, Supporting Information), indicating the oligomers continued to undergo polymerization reactions after they were deposited on the surface of the incipient film. The spectra of attenuated total reflectance Fourier‐transform infrared spectroscopy (ATR‐FTIR) show that some carboxyl acid groups (1700 cm^−1^) in both the incipient film and oligomers are converted into amide bonds (1656 cm^−1^) during the formation of growth layers (**Figure** **4**a). Additionally, a compact structure of the 3P membrane is observed from the cross‐sectional high angle ring dark field (HAADF) images (Figure 4c), indicating that the incipient film and growth layers are tightly linked due to the reaction between them. The analysis of the angular resolution X‐ray photoelectron spectroscopy (ARXPS) indicates that the incipient film with lower crosslinking degree will result in the growth layer with higher crosslinking degree (Figures S17–S19, Supporting Information). This suggests that some MPD monomers involved in the reaction may come from the aqueous phase beneath the incipient film. The cross‐sectional HAADF images of the incipient film (Figure 4c) present several paths in which silver ions are enriched. MPD molecules in the aqueous solution may enter the organic phase along these paths to participate in SP reaction.

Based on the above discussion, the formation mechanism of growth layers is described in Figure 4d. Briefly, the newly sprayed organic droplets meet the residual aqueous solution, creating a new aqueous/organic interface. MPD molecules in aqueous phase continue to diffuse into organic phase through the interface, participating in the reaction with TMC molecules and oligomers to form PA layers. In the cross‐sectional HAADF images of the 3P membranes, silver ions cannot be observed (Figure 4c), indicating that the SP reaction makes the oligomers denser and pore size smaller so that silver ions cannot diffuse downward. It is deduced that MPD molecules can diffuse through the channels and participate in the SP reaction at the top of the channels. This helps to reduce the size of the upper part of the channel, thereby inhibiting the film defect generation. In addition, the use of MPD organic solutions is helpful to break diffusion‐limitation in conventional IP and avoid contact between acyl chloride bonds and aqueous phase. Therefore, the invented coupled polymerization method significantly solves the problems of ES‐IP. (Note S2.4; Figure S2, Supporting Information).

In addition, the unique film formation mechanism endows the characteristic structures of the PA layer. First, the thicknesses of these 3P membranes are 13.1, 13.4, 14.3, 14.7, and 14.8 nm, respectively (Figure 3b). Linearity in film growth with an increasing number of scans is observed for these membranes, and each organic solution spray results in a growth layer with a thickness of ≈ 2.2 nm (Tables S5 and S6, Supporting Information). Thus, control of thickness per scan is notably consistent. Second, the average roughness of each PA layer in the 3P membrane is less than 2 nm, as shown in Figure 3b and Table S6 (Supporting Information). Lastly, the photothermal induced resonance (PTIR) images of the incipient film show many aggregated red spots and dotted blue spots (Figure 4b). By comparing the FTIR spectra with PTIR spectra, they are identified as amine and carboxyl groups on the formed polyamide matrix, respectively (Figure S15, Supporting Information). It is deduced that after complete evaporation of organic solvents, the diffusion of reactants across the aqueous/organic interface is terminated and IP reaction is stopped, leaving unreacted amine and acyl chloride groups. Then, these acyl chloride groups undergo hydrolysis with water molecules in the environment, producing carboxyl groups. The PTIR images of the third growth layer present uniformly distributed white spots but with fewer red and blue spots. By combining the PTIR spectra, it can be deduced that ES‐HP‐SP causes relatively complete polymerization between MPD molecules and acyl chloride groups, producing amide bonds with even distribution (Figure 4b).

### 2.3 Customizable Printing of PA Layer with Numerical Predication Model

According to the unique mechanism of ES‐3P, we developed the numerical predication model mentioned above to aid customizable printing based on electrohydrodynamic and kinetics analysis (Figure S4; Note S3, Supporting Information). This model includes three sections corresponding to droplet generation, solvent volatilization, and polymerization reaction, respectively. The generated droplets from the needles flied onto the collector and at the same time solvents underwent volatilization. The diameter of droplets was calculated using the scaling laws proposed by Gañán‐Calvo et al.^[^ ^37^ ^]^ and the equations proposed by Chowdhury et al.^[^ ^33^ ^]^ (Figure S20a,b, Supporting Information). The deposited droplets formed a layer of solution on the collector, which finally disappeared due to solvent volatilization. The time for solvent volatilization was calculated based on two‐film theory (more details in Note S3.2; Figure S20c, Supporting Information).

**Figure 5:**
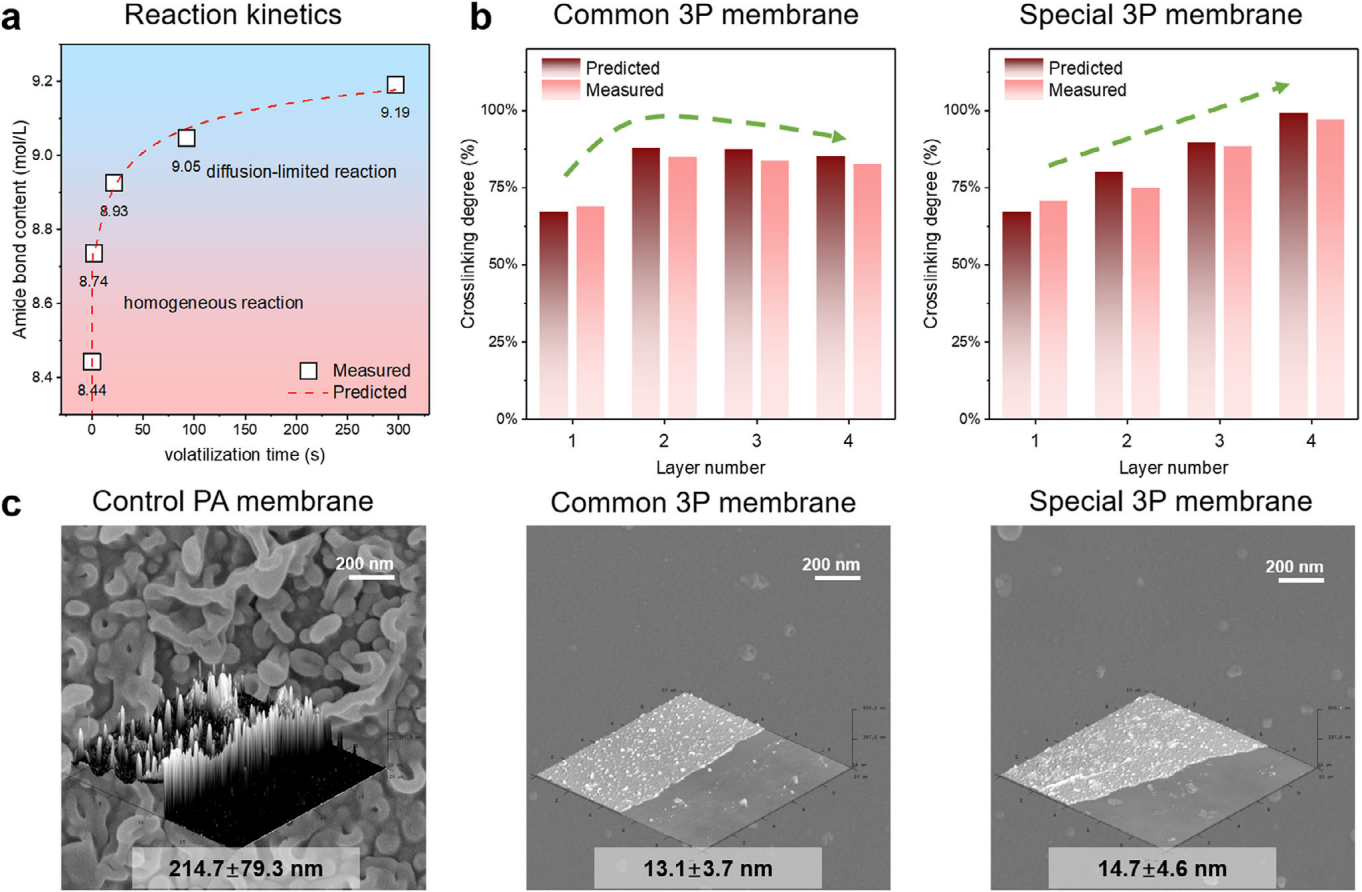
Programmable crosslinking structure and controlled morphology of the common and special 3P membranes. a) Calculated amide bond content by measured data and the predicted amide bond content by the numerical prediction model. b) Predicted and measured crosslinking degree of each PA layer in the common and special 3P membranes. c) SEM images and AFM 3D images of the control PA membrane and the common and special 3P membranes.

For the formation of the incipient film, a homogeneous reaction between MPD and TMC monomers occurred at the beginning, followed by a diffusion‐limited reaction. For the formation of the growth layers, MPD and TMC monomers underwent homogeneous polymerization and produced oligomers. After the deposition of oligomers on the collector, the residual MPD monomers in the aqueous phase diffused across interface, resulting in a diffusion‐limited reaction with the unreacted acyl chloride groups of oligomers. Based on these formation mechanisms of the incipient film and growth layers, the increase of amide bonds contents could be calculated by Equation S25 (Supporting Information) (More details in Note S3.3, Supporting Information). The values of the parameters in Equation S25 (Supporting Information) were estimated by nonlinear fitting between the solvent evaporation time and amide bond contents based on the XPS and AFM analysis of the incipient film (Note S4.1; **Figure** **5**a; Table S4, Supporting Information). With the values of amide bonds contents, the crosslinking degree and thickness of PA layers were predicted using Equations S32 and S33 (Supporting Information) by substituting the monomer concentration and solvent evaporation time, respectively.

Figure 5b shows the errors between the predicated crosslinking degrees of common 3P membrane by the model and the measured crosslinking degrees from the ARXPS analysis^[^ ^38^ ^]^ are less than 10%. In addition, the predicted thickness is also very close to the measured thickness (Table S5, Supporting Information). This confirms the applicability of the model and indirectly proves the above film formation mechanism.

Based on this model, we obtained the quantitative relationships between the features of the PA layer (thickness, crosslinking degree, amide bond density, etc.) and the printing parameters (organic solvent combination, monomer concentration, polymerization time, etc.), as shown in the growth curves in Figure S20 (Supporting Information). With the help of these growth curves, we designed and fabricated a special 3P membrane with controllable thickness, as well as with the programmable crosslinking degrees for each PA layer. The printing strategy and parameters are shown in Note S2 and Table S2 (Supporting Information), respectively. All the printed PA layers have a significantly flatter structure rather than the typical ridge‐valley morphologies of conventional PA films (Figure 5c). It makes the surface roughness of 3P membrane only 3 nm higher than that of the substrate (Figure S24, Supporting Information). Moreover, the pore diameter distribution of the printed PA membranes is very narrow (≈0.32 nm) (Figure S21, Supporting Information), consistent with the uniform distribution of functional groups at the surface of PA layer. These are attributed to not only the low monomer concentration but also the layer formation mechanism. The consistency of the measured and calculated film thickness indicates that the PE3DP produces the PA layer with controllable thickness by controlling the TMC concentration and polymerization time (Table S5, Supporting Information). To endow a special 3P membrane with programmable crosslinking degree (80%, 90% and 99%), MPD concentration and organic solvent combination varied layer by layer. The MPD concentrations used in each layer preparation were 0.003%, 0.003% and 0.012%, respectively, and the organic solvent used were 10% n‐decane + 90% n‐hexane, 100% n‐decane, 100% n‐decane, respectively (Table S2, Supporting Information). The errors between the calculated and measured crosslinking degree are less than 10% for all the growth layers (Figure 5b). This indicates that the PE3DP realized customizable printing of PA layers with programmable crosslinking degree.

### 2.4 Separation Performance of the Common and Special 3P Membranes

**Figure 6:**
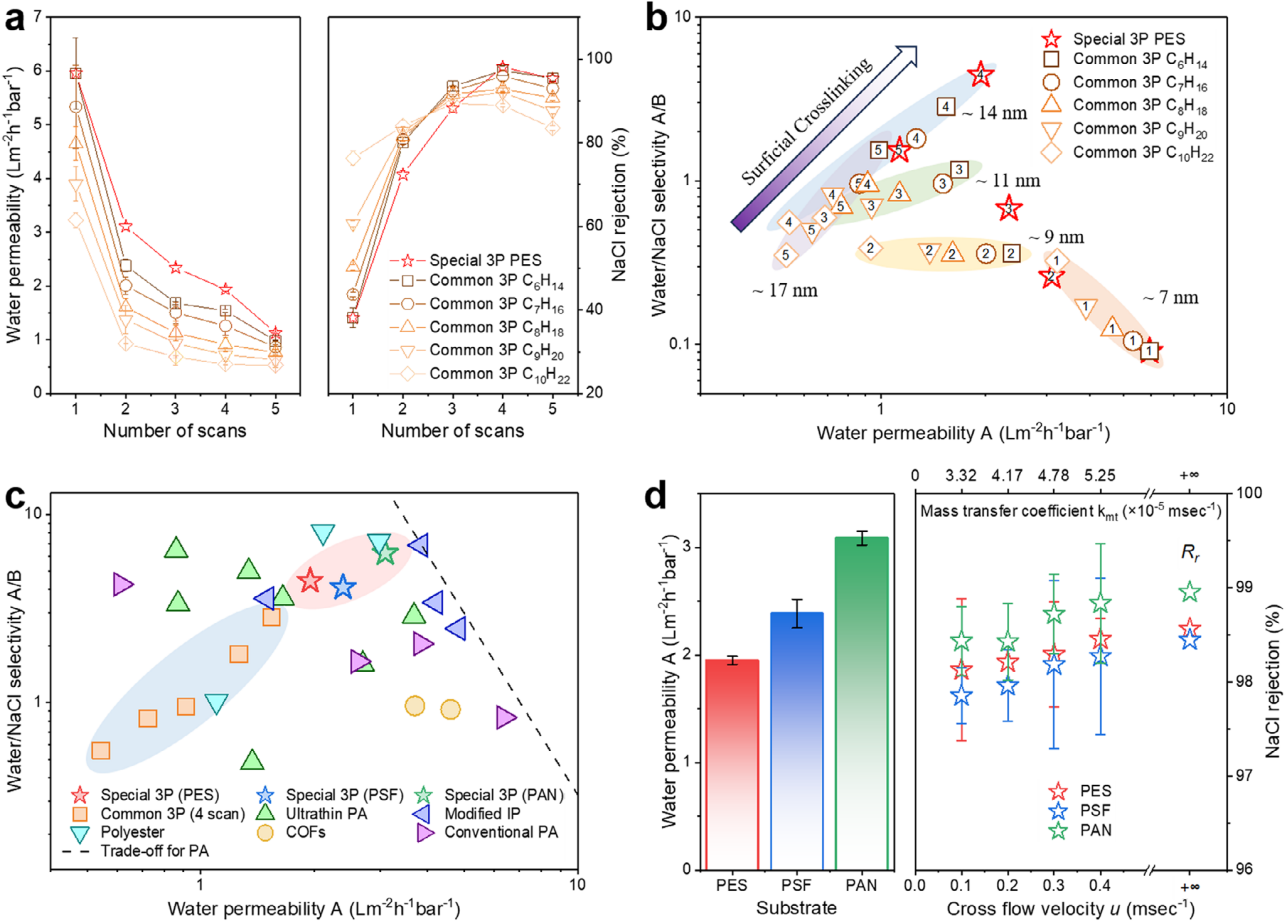
Separation performance of the common and special 3P membranes. a) Water permeability (left) and salt rejection (right) of the common and special 3P membranes with different number of scans. b) Permselectivity of the common and special 3P membranes (the number represents the number of scans). c) Permselectivity of 3P membranes and reported RO membrane (including ultrathin PA membrane,^[^ ^33^, ^41^, ^42^, ^43^, ^44^, ^45^ ^]^ modified IP membrane,^[^ ^46^, ^47^, ^48^ ^]^ polyester membrane,^[^ ^49^, ^50^, ^51^ ^]^ covalent organic frameworks membrane^[^ ^52^, ^53^ ^]^ and conventional PA membrane^[^ ^21^, ^33^ ^]^). d) Effect of substrate (left) and polarization (right) on special 3P membranes.

The separation performance of these films was analyzed by crossflow filtration tests. The incipient films present high water permeance (6.0 LMH bar^−1^) but low NaCl rejection (53.7%) (**Figure** **6**a). Longer solvent volatilization time formed thicker and higher crosslinked films, which led to less water permeable and higher NaCl rejection. With the formation of growth layers, the water permeance of the 3P membrane decreased, and its salt rejection increased. Three growth layers with a thickness of ≈14 nm show the best performance (Figure 6b). Beyond this threshold, the further increase of thickness leads to a decline in both water transport and salt rejection. This is attributed to the insufficient MPD molecules diffusion from the aqueous phase, resulting in a loosely topmost, which deteriorates the permselectivity of the membranes. The common 3P membrane fabricated by three scans with organic solvent of C_6_H_14_ for preparing the incipient film presents a water flux of 1.5 LMH bar^−1^ and a salt rejection of 97.3%, comparable to those of the industry‐standard conventional PA membrane (Figure 6c). This indicates that lower crosslinking degree in the incipient film and higher crosslinking degree in the growth layer resulted in enhanced water permeability and water/salt selectivity. It is probably attributed to the weaker funnel effect and higher dehydration energy of the active separation layer with this structure.^[^ ^25^, ^39^ ^]^

The performance of the 3P membranes was further optimized by creating the PA layer with programmable crosslinking degree. The special 3P membrane presents obviously better performance than the common 3P membrane and control PA membrane, with a water flux higher than 2.0 LMH bar^−1^ and a salt rejection higher than 98.0% (Figure 6a). This indicates that the PA layer with gradually increasing crosslinking degree is more conducive to improving permselectivity than the PA layer with constant crosslinking degree or gradually decreasing crosslinking degree. Due to the self‐gutter effect of the higher permeability loose film, this structure of PA layer reduces the overall transport pathways, significantly increasing the water permeation while maintaining salt rejection and encountering less concentration polarization and less fouling. The more permeable substrate resulted in higher permeance of the special membranes (PSF: 2.4 LMH bar^−1^; PAN: 3.1 LMH bar^−1^), while maintaining similar salt rejection (PSF: 97.9%; PAN: 98.4%) (Figure 6d). When the membranes were tested with high crossflow velocity, higher salt rejection were obtained due to the diminishing of concentration polarization. The real salt rejections (Rr) estimated by the correction of mass transfer coefficient were 98.6%, 98.4% and 99.0% for PES, PSF and PAN substrates, respectively.

The separation performance of the 3P membrane was compared with that of the state‐of‐the‐art RO membranes (Figure 6c). The performance of the special 3P (PAN) membrane is better than most of these RO membranes. In addition, it approaches the upper limit of the selectivity‐permeability trade‐off relationship of the PA membrane. It should be mentioned that this work is to establish a customizable manufacturing method for membranes, rather than to outperform an industry‐standard membrane. Conventional IP typically produces PA layer with the loose upper segment and dense lower segment.^[^ ^39^ ^]^ However, this configuration suffers more severe concentration polarization and fouling.^[^ ^40^ ^]^ In contrast, the PE3DP could produce an asymmetric PA structure consisting of the increasingly dense growth layers in the upper part and the loose incipient film attached to the porous substrate at the bottom. This structure enables superior water permeability and water/salt selectivity. Therefore, customizable printing with the PE3DP produces PA layers with controlled morphology and programmable crosslinking structure, ultimately leading to high‐performance membranes for efficient desalination with RO technology.

## 3 Conclusion

The developed programmable electrospray printer can realize the customizable printing approach to fabricate spatially heterogeneous PA membranes with controllable morphology and programmable crosslinking structure. The thickness of the layer is as low as 14 nm with as little as 3‐nm resolution and the surface of the layer is intrinsically smooth with the roughness of ≈4 nm. These features of the layer are helpful to reduce the effective water transport length of PA layer and fouling propensity. Additionally, the programmable crosslinking structure with the crosslinking degree increasing layer by layer is helpful to reduce funnel effect. The top dense layer is responsible for high ion selectivity, while the lower loose layer is responsible for high permeability during the filtration process. This structure enables the membranes to achieve a water flux of 3.1 L·m^−^^2^·h^−1^·bar^−1^ and NaCl rejection and 99.0%. With the help of the numerical model, we can not only operate the electrospray printer but also design and fabricate the PA layer with well‐designed features to endow the membranes with excellent performance such as high permselectivity, strong antifouling and anti‐polarization ability.

This method can be readily integrated with the existing production line of membrane production. Figure S22 (Supporting Information) shows a prototype of printer for industrial scale‐up. Here, a flat plate collector serving as the negative electrode lifts the UF membrane upward, while a multi‐nozzle array works as the positive electrode. Nozzles in the same row spray identical solutions to prepare a PA layer, and different rows correspond to specific layers in this study. This configuration significantly reduces the consumption of MPD and TMC monomers and solvents, maintaining economic feasibility.

The adaptation of this printing approach to other monomers or interfacial reactions might enable the development of other polymer membranes for use in other separations. This work brings a transformation of electrospray into a customizable manufacturing technology, offering a universal platform for on‐demand fabrication of complex‐structured nanoscale membranes through fully automated control and multi‐material compatibility.

## Conflict of Interest

The authors declare no conflict of interest.

## Supporting information



Supporting Information

Supplemental Data

Supplemental Video 1

Supplemental Video 2

Supplemental Video 3

Supplemental Video 4

## Data Availability

The data that support the findings of this study are available from the corresponding author upon reasonable request.
