# Supplementary material for: Customizable Manufacturing of Polyamide Membranes with Programmable Layers and High Permselectivity by Electrospray Printer
Source: Adv Sci (Weinh). 2025 Sep 12;12(45):e09127. doi: 10.1002/advs.202509127 (PMC12677634; doi:10.1002/advs.202509127)
Supplement: Supplementary file 1 — Supporting Information [file ADVS-12-e09127-s003.docx]

Supporting Information

**Customizable Manufacturing of Polyamide Membranes with Programmable Layers and High Permselectivity by Electrospray Printer**

*Xieyang Xu, Yingsong Liu, Huijun Yu, Chenshuo Wang, Pin Hou, Huijiao Wang, Yanjun Liu, Kai Zhang, Peidong Su, Chunrong Wang and Jianbing Wang**

The Supporting Information text includes:

Note S1 to S5

Figure S1 to S23

Table S1 to S7

References ^[1-66]^

Other supporting materials include:

Video S1 to S4

Code S1

**Table of Contents**

[Note S1 Materials 3](#_Toc204372608)

[Note S2 Programable electrospray printer and methods for PA layer printing 4](#_Toc204372609)

[Note S3 Development of the numerical prediction model 8](#_Toc204372610)

[Note S4 Application of the numerical prediction model 15](#_Toc204372611)

[Note S5 Characterization and analysis methods 18](#_Toc204372612)

[Figure S1. 25](#_Toc204372613)

[Figure S2. 26](#_Toc204372614)

[Figure S3. 27](#_Toc204372615)

[Figure S4. 28](#_Toc204372616)

[Figure S5. 29](#_Toc204372617)

[Figure S6. 30](#_Toc204372618)

[Figure S7. 31](#_Toc204372619)

[Figure S8. 32](#_Toc204372620)

[Figure S9. 33](#_Toc204372621)

[Figure S10. 34](#_Toc204372622)

[Figure S11. 35](#_Toc204372623)

[Figure S12. 36](#_Toc204372624)

[Figure S13. 37](#_Toc204372625)

[Figure S14. 38](#_Toc204372626)

[Figure S15. 39](#_Toc204372627)

[Figure S16. 40](#_Toc204372628)

[Figure S17. 41](#_Toc204372629)

[Figure S18. 42](#_Toc204372630)

[Figure S19. 43](#_Toc204372631)

[Figure S20. 44](#_Toc204372632)

[Figure S21. 45](#_Toc204372633)

[Figure S22. 46](#_Toc204372634)

[Figure S23. 47](#_Toc204372635)

[Table S1. 48](#_Toc204372636)

[Table S2. 49](#_Toc204372637)

[Table S3. 50](#_Toc204372638)

[Table S4. 51](#_Toc204372639)

[Table S5. 52](#_Toc204372640)

[Table S6. 53](#_Toc204372641)

[Table S7. 54](#_Toc204372642)

[Video S1. 55](#_Toc204372643)

[Video S2. 55](#_Toc204372644)

[Video S3. 55](#_Toc204372645)

[Video S4. 55](#_Toc204372646)

[Code S1. 55](#_Toc204372647)

[References 56](#_Toc204372648)

Note S1 Materials

Three types of commercial ultrafiltration (UF) membranes were purchased from RisingSun Membrane Engineering & Technology Co. Ltd. They are US020 (Polysulfone based UF membrane), UE020 (Polyether sulfone based UF membrane) and UN100 (Polyacrylonitrile based UF membrane), respectively. These UF membranes were used as porous substrates, and their characteristics are shown in Table S7. A commercial SW30XLE RO TFC membrane from Dow was used to benchmark performance against our membranes. 1,3,5-benzenetricarbonyl trichloride (TMC), n-hexane, n-heptane, n-octane, n-nonane, n-decane and ethyl alcohol were purchased from J&K Scientific Co. Ltd. (Beijing). Ionic liquid [Tri-hexyl tetradecyl phosphonium bis-(trifluoro methyl sulfonyl) amide] were received from Sigma-Aldrich Co. Ltd. m-Phenylenediamine (MPD) obtained from Aladdin Chemistry Co. Ltd. Other chemicals were obtained from Shanghai Macklin Biochemical Technology Co. Ltd. Deionized (DI) water with a resistance of 18.2 MΩ cm was used in all experiments. All reagents were of analytical standard and used without any further purification.

Note S2 Programable electrospray printer and methods for PA layer printing

2.1 Programable electrospray printer

The developed programmable electrospray 3D printer (PE3DP) mainly comprises a printing head, printing material supply system, collector system, motion system, power supply system and control system. Figure S1a shows the photo of the PE3DP, and the schematic description is shown in Figure 1.

The printing head of the PE3DP is assembled by an aqueous nozzle (a separate needle) and an organic nozzle (a multi-in-one device with 4 needles). These 5 needles (316L, 30G) are fixed in the polylactic acid (PLA) structural components. The separate needle is in the vertical direction, while the other 4 needles are tilted to form a four-cone shape, with the needle tips facing each other. The tilt angle is 14.7°. This allows the organic solutions that sprayed from the 4 needles to form a Talyor cone, and then, the MPD and TMC monomers deposit on the collector simultaneously for undergoing polymerization reaction.

The printing material supply system is used to supply printable materials (MPD and TMC monomers) to the printing head during the electrospray process. It is composed of injection pumps and PTFE pipes. The injection pumps and needles are connected through the PTFE pipes. Each injection pump is controlled by a stepper motor.

The collector system uses a flat plate (a mechanical stage) to attach a UF membrane substrate on its surface for collecting the printable materials (MPD and TMC monomers). The UF membrane substrate is placed onto a grounded electrode. The grounded electrode is insulated from the mechanical stage by a polytetrafluoroethylene (PTFE) plate. Additionally, a heating bed layer with a temperature sensor is set between the mechanical stage and PTFE plate.

The motion system is used to realize the 3D relative motion between the printing head and the mechanical stage. The printing head is attached on a roller slide rail and moves along the X-axis direction. At the same time, the roller slide rail is attached on a gantry structure and moves along the Z-axis direction. The mechanical stage is attached on a roller slide rail and moves along the Y-axis direction. This design ensures that the printing needles can move along the X-axis and Z-axis directions, while the collector can move along the Y-axis direction. Their movement is controlled by the X/Y/Z motors and drivers.

The power system is used to supply low voltage and high voltage power to the PE3DP. A high-voltage DC power source that can generate up to 20 kV is used for electrospray. Low voltage (0~24 V) is used for the operation of motherboard, stepper motors, fans and hot bed.

The control system is used to coordinate the printing material supply, power supply, motion control and temperature control of the PE3DP. It is composed of BIGTREETECH Octopus Pro V1.0 motherboard and BIGTREETECH Pi V1.2 host computer (all from BIQU Technology Co., Limited). Both motherboard and host computer are equipped with the Klipper firmware (https://www.klipper3d.org/) for the execution of Gcode files. Cartesian kinematics is used to realize the cooperative control between electrospray and 3D motion.

2.2 Preparation for Electrospray printing

Firstly, a UF membrane was attached at the desired position of collector and secured with conductive tapes. Disposable syringes were loaded with aqueous and/or organic solutions and connected to the PTFE pipes of the nozzle group. The PTFE pipes were filled with solutions and the needle tips were wiped with an alcohol pad to remove dust or solvent.

Secondly, the surface of the collector was heated to the predetermined temperature (25℃ in this study) by using the heating bed. Coordinating correction and zeroing were performed for the gantry structure. The needles raised to the predetermined height (20 mm), moved to the preparation area and reciprocated. At this time, the high-voltage power supply turned on and the injectors pushed the solution to form spray.

To keep both aqueous and organic solution sprays in cone-jet mode, the applied voltage and ionic liquid concentration were optimized many times. Firstly, the voltage was tuned carefully, and a voltage window was found for keeping aqueous solution spray in cone-jet mode. Secondly, lipophilic ionic liquid was added into non-polar alkanes, so that the organic solution spray was also kept in cone-jet mode. The amount of lipophilic ionic liquid was also carefully optimized, and the results are shown in Table S1. Finally, fine adjustment of voltage was conducted, and a very narrow voltage window (4.3~4.6 kV) was obtained for electrospray (Figure 2b).

Finally, the optimal electrospray conditions were obtained, and both aqueous and organic solution sprays could form circular deposition on the collector, as shown in Figure S6. When electrospray was stabilized, the printing needles moved along the predetermined path above the substrate.

2.3 Procedures for PA layer printing

For preparing the 3P membranes, an incipient film was first printed and then several growth layers were printed. For fabricating the incipient film, the aqueous nozzle and organic nozzle extruded MPD aqueous solution and TMC n-alkane solution directly to the flat plate collector, respectively. As monomer solutions emerged from the needle tips, many droplets were formed in the presence of a strong electric field. These droplets flied onto the surface of the collector. The immiscibility between MPD aqueous droplets and TMC organic droplets permitted the interfacial polymerization reaction to occur at the phase boundary.

The aqueous nozzle and organic nozzle were arranged in the x direction and travelled in a zig-zag path above the surface of the collector (Figure S1b). The path spacing was 1 mm, ensuring an approximate 90% overlap. The moving speed was 10 mm/s. A single pass over the surface of the collector is referred to as a “single scan.” After a single pass, an ultrathin incipient film was formed on the surface of porous substrate due to the interfacial polymerization reaction between MPD and TMC monomers. When fabricating the incipient films, the concentration of MPD and TMC monomers were 0.2%:0.010%, respectively. Five types of organic solvents (n-hexane, n-heptane, n-octane, n-nonane and n-decane) were used to dissolve TMC monomers.

After the formation of the incipient film, the extrusion of MPD aqueous solution and TMC n-alkane solution was stopped. Then, three growth layers were printed by electrospray to prepare the 3P membranes, with one layer for each scan. During preparation, 4 needles in the multi-in-one device extruded MPD/ethanol/n-hexane solution (Needle #1), TMC/n-hexane solution (Needle #2), n-hexane liquid (Needle #3) and n-decane liquid (Needle #4), respectively. These solutions were mixed and MPD and TMC monomers in the mixed organic solution underwent homogeneous polymerization (HP) in about 0.3 seconds to produce oligomers. By adjusting the tilt angle of the needles and spray parameters, a stable Taylor cone spray of the mixed solutions was obtained. Then, the mixture was deposited on the collector and grew into a highly cross-linked polymer layer.

After one scan, the needles returned to the starting position at a speed of 50 mm/s (taking approximately 5 seconds) and the next scan was initiated. For each scan, the total injection speed of the multi-in-one device was kept constant (1.55 mL/h). The monomer concentrations and organic solvent combinations for preparing the growth layers were determined based on calculations using the numerical model. For instance, when a growth layer in the 3P membrane is designed with a thickness of 2.2 nm, the calculated TMC concentration is 0.005%. After a specific TMC concentration is selected, the growth curves of crosslinking degree can be obtained using the numerical model (Figure S20). The growth curves show that when the MPD concentration is 0.003%, crosslinking degree will increase to 85% at a volatilization time of 50 seconds. To keep the volatilization time of organic solvents within 50 seconds, the ratio of n-hexane to n-decane in the mixed organic solution should be controlled at 5:1.

Initially, the same organic solutions were used to prepare three growth layers, which contained 0.003% MPD and 0.005% TMC with n-decane as the solvent. The obtained 3P membranes were referred to as common 3P membranes. Then, in order to prepare special 3P membranes with crosslinking degree increasing layer by layer, the monomer concentration and solvent combinations of the organic solution for preparing each layer were determined using the method mentioned above. 0.2% MPD aqueous and 0.010% TMC n-hexane solutions were used to print the incipient films. For the fabrication of the first growth layer, the mixed organic solution contained 0.003% MPD and 0.005% TMC, and the solvent consisted of 10% n-decane and 90% n-hexane. For the fabrication of the second growth layer, the mixed organic solution contained 0.003% MPD and 0.005% TMC, and solvents consisted of 100% n-decane. For the fabrication of the third growth layer, the mixed organic solution contained 0.012% MPD and 0.005% TMC, and solvents consisted of 100% n-decane. Further details of the operating parameters are provided in Table S2.

After electrospray printing, the membranes were placed in an oven and cured at 80℃ for 10 min to completely volatilize the solvent and the ionic liquid. The cured membranes were kept in deionized water at 4℃, pending characterization or separation performance tests.

2.4 Shortage of ES-IP and ES-HP methods in PA membrane printing

The ES-IP, ES-HP, and ES-SP methods were used for printing the PA membrane. The formation process and structure of the PA membrane are shown in Figure S2.

In the ES-IP process, aqueous solution and organic solution are deposited alternately on the substrate. It promotes the hydrolysis of acyl chloride, which can reduce the crosslinking degree of PA layers. As a result, there were more carboxy groups at the top and more amine groups at the bottom of each PA layer (Figure S2a). This makes it difficult to customize the structure of PA layers by ES-IP method.

In the ES-HP process, all MPD and TMC are dissolved in the organic phase. Therefore, homogeneous polymerization occurs in the organic phase, resulting in the formation of loose oligomers of PA. These oligomers are then deposited on the water/alkane interface. Because no MPD monomers are dissolved in the water phase, oligomers cannot be fixed and further crosslinked, resulting in the formation of very loose PA layer (Figure S2b). In our experiments, when DMF was used to dissolve the substrate, the PA layer prepared using the ES-HP method was immediately broken. ES-HP is also unsuitable for the customizable printing of PA layers.

The combination of ES-IP, ES-HP and ES-SP (ES-3P) is different from single ES-IP or single ES-HP. With the protection of the incipient layer and the organic solvent, the amine groups and the acyl chloride groups on the upper surface of the incipient layer (from ES-IP) and the PA oligomers (from ES-HP) will not be hydrolyzed. Additionally, during the preparation of the growth layer, MPD in water phase can diffuse upward, which plays a key role in fixing and crosslinking the oligomers. Under these conditions, it is possible to prepare dense PA films layer-by-layer and independently adjust their thickness and crosslinking degree (Figure S2c).

2.5 Development of Gcode files from the slicing program for PA layer printing

In this work, Gcode files were generated from the slicing program for PA layer printing. We used Python to write slicing programs in the Pycharm software system (PyCharm Community Edition 2023.3.5, JetBrains s.r.o, https://www.jetbrains.com/zh-cn/pycharm/), which converted the procedures of PA layer fabrication into Gcode files. The Gcode files can be recognized by the Klipper firmware of the microcomputer chip in the PE3DP", which can drive stepper motors to conduct the printing procedures according to the Gcode files.

Figure S3 shows a flowchart of the slicing program that we developed in the Pycharm software system. First, the printing data are input into the slicing software. The data include blueprints, solution concentration, operating parameters and printer setup. The blueprints are then pre-processed and divided into different printing areas of different layers. Finally, the boundaries of each print area are identified, and the print path is generated. Spray solutions are switched between different printing areas by changing the push speed of different injectors.

The slicing software, which converts the procedures of PA layer fabrication into Gcode files, is uploaded as an attachment.

Note S3 Development of the numerical prediction model

A numerical model for 3P membranes was developed based on electrohydrodynamic and kinetic studies. Using this numerical model, certain features of the PA layer properties can be predicted according to the operation parameters of the PE3DP.

The model was divided into three parts: droplet generation, solvent volatilization and polymerization reaction, as shown in Figure S4.

3.1 Droplet generation

During electrospray, monomer solutions left the needles in the presence of a strong electric field. They were sprayed in the form of droplets and deposited onto the surface of the collector. The diameter of droplets in cone-spray mode is estimated according to the scale law proposed by Gañán-Calvo et al.^[37]^.

For the MPD aqueous solution, which has a high surface tension (72.8 N/mm), the droplet diameter can be calculated as follows:

$D_{p}=\left(1.2\left[\frac{Q}{\gamma_{s}\varepsilon_{0}/\left(\rho_{s}K_{s}\right)}\right]-0.3\right)\times\left(\gamma_{s}\varepsilon_{0}^{2}\rho_{s}K_{s}^{2}\right)^{1/3}$ (S1)

where, *D_p_^r^* is the reference droplet diameter, m; *Q^r^* is the reference flow rate, m^3^/s; *γ_s_* is the surface tension of the solution, N/m; ε_0_ is the dielectric constant of vacuum, 8.854×10^−12^ C^2^/(N·m^2^); ρ_s_ is the solution density, kg/m^3^; *K_s_* is the conductivity of the solution, s·C^2^/kg·m^2^; μ*_a_* is viscosity of air, Pa·s; *Q* is the actual flow rate, m^3^/s; ε*_s_* is the relative dielectric constant of the solution

For the organic solutions, which have a low surface tension (22.8 N/mm), the droplet diameter can be calculated as follows:

$D_{p}=\left\{1.6\left[\frac{Q}{\left(\varepsilon_{s}-1\right)^{1/2}\gamma_{s}\varepsilon_{0}/\left(\rho_{s}K_{s}\right)}\right]-1.0\right\}\times\left(\varepsilon_{s}-1\right)^{1/3}\times\left(\gamma_{s}\varepsilon_{0}^{2}\rho_{s}K_{s}^{2}\right)^{1/3}$ (S2)

3.2 Solvent volatilization

During electrospray, the ejected droplets flew onto the surface of the collector. When the droplets flew, the solvent volatilized, resulting in a decrease in droplet diameter^[37]^. The diameter at time (t) was denoted as *D_p_*(t). Specifically, the diameter at the beginning of flight was denoted as *D_p_*(0), which can be calculated using Equation (S1) and (S2). When the droplets were deposited on the surface of the electrode, their diameters were denoted as *D_p_*(*t_D_*). Henry's law can be used to calculate the diameter during flight, which was reported by Chowdhury et al.^[33]^. Based on their previous work, Equation (S3) was obtained:

$\left\{\begin{matrix} z=D_{p}\left(t\right)^{1/4} \\ \frac{C}{6A}z^{6}-\frac{C}{3A^{2}}z^{3}+\frac{C}{3A^{3}}\ln\left(Az^{3}+1\right)=Bt+D \\ A=0.276\left(\frac{\mu_{a}}{\rho_{a}D_{sa}}\right)^{1/3}\times\left(\frac{\rho_{a}}{\mu_{a}}\times\frac{\left(\varepsilon_{0}\gamma_{s}\right)^{1/2}\Phi}{f_{d}\mu_{a}H}\right)^{0.5} \\ B=-\rho_{a}D_{sa}\ln\left\{1-\left(\frac{M_{s}}{M_{\infty}}\right)\exp\left[\frac{LM_{s}}{RT_{bw}}\right]\left(1-\frac{T_{bw}}{T_{L}}\right)\right\} \\ C=\frac{2\pi\rho_{s}}{2} \\ D=\frac{C}{6A}D_{p}\left(0\right)^{\frac{3}{2}}-\frac{C}{3A^{2}}D_{p}\left(0\right)^{\frac{3}{4}}+\frac{C}{3A^{3}}\ln\left(AD_{p}\left(0\right)^{\frac{3}{4}}+1\right) \end{matrix}\right.$ (S3)

where, *t* is the flight time, s; Ф is the potential difference between the needles and surface of the collector, V; ƒ_d_ is the viscous drag coefficient; μ_𝑎_ is viscosity of air; 𝐻 is the needles to plate collector distance, mm; ρ*_a_* is the air density, kg/m^3^; 𝐷_s𝑎_ is diffusivity of solvent in air; 𝑀_𝑠_ is molecular weight of solvent; 𝑀_∞_ is molecular weight of air; 𝐿 is latent heat of vaporization of solvent, J/kg; 𝑅 is global gas constant, J/(mol·K); 𝑇_𝑏_*_s_* is boiling point of solvent, K; 𝑇_𝐿_ is liquid surface temperature, K.

The flight distance is the integral of flight speed over time. Therefore, Equation (S4) is obtained:

$H=\int_{0}^{t_{D}} v\left(t\right)dt=\frac{\left(\varepsilon_{0}\gamma_{s}\right)^{1/2}\Phi}{f_{d}\mu_{a}H}\times\int_{0}^{t_{D}} D_{p}\left(t\right)^{\frac{1}{2}}dt$ (S4)

By solving the Equation (S3) and Equation (S4), the values of *t_D_* and *D_p_*(*t_D_*) are obtained.

After the droplets were deposited on the surface of the collector, they spread out and formed a layer of solution. The solvent would continue to volatilize, and the thickness of the solution layer would decrease. The volatilization rate of alkane solvent, *dn/dt* (mol/s), can be estimated according to the two-film theory.

$\frac{dn}{dt}=Sk_{sa}\left(p_{s}-p_{s}*\right)$ (S5)

where, *S* is the membrane area; *k_sa_* is the mass transfer coefficient from solvent to air; *p_s_* is saturated vapor pressure of solvent; *p_s_^*^* is the partial pressure of the solvent in the air.

*k_sa_* is calculated with follow equation:

$k_{sa}=\frac{D_{sa}}{RT\delta_{a}}\times\frac{p_{a}}{p_{s}}\ln\left(\frac{p_{a}}{p_{a}-p_{s}}\right)$ (S6)

where δ*_a_* is the thickness of the gas film (0.4 mm in this study); *p_a_* is atmospheric pressure.

The change of the thickness of the solution layer is calculated using the following equation:

$\frac{dl_{s}}{dt}=\frac{M_{s}}{\rho_{s}S}\frac{dn}{dt}$ (S7)

where *M_s_* is the molar mass of solvent; *ρ_s_* is the density of the solvent.

By substituting the Equation (S5) and (S6) into (S7), Equation (S8) is obtained:

$\frac{dl_{s}}{dt}=\frac{M_{s}}{\rho_{s}}\left(p_{s}-p_{s}*\right)\frac{D_{sa}}{RT\delta_{a}}\times\frac{p_{a}}{p_{s}}\ln\left(\frac{p_{a}}{p_{a}-p_{s}}\right)$ (S8)

As the concentrations of MPD and TMC were very low, their effects on the volatilization of the solvent were neglected in this study. Therefore, the solvent evaporation time *t_V_* is calculated using the following equation:

$t_{V}=\frac{l_{s}\left(0\right)}{\frac{M_{s}}{\rho_{s}}\left(p_{s}-p_{s}*\right)\times\frac{D_{sa}}{RT\delta_{a}}\times\frac{p_{a}}{p_{s}}\ln\left(\frac{p_{a}}{p_{a}-p_{s}}\right)}$ (S9)

where, *l_s_*(0) is the initial thickness of the solution layer.

The *l_s_*(0) is calculated with the following equation:

$l_{s}\left(0\right)=\frac{Q_{ES}}{d_{ES}v_{ES}}\times\left(\frac{D_{p}\left(t_{D}\right)}{D_{p}\left(0\right)}\right)^{3}$ (S10)

where, *Q_ES_* is the injection speed during electrospray; *d_ES_* is the spacing of the printing paths during electrospray; *v_ES_* is the moving speed of the needles during electrospray.

By substituting Equation (S9) into (S10), Equation (S11) for calculating the volatilization time (*t_V_*) is obtained:

$t_{V}=\frac{\frac{Q_{T}}{d_{ES}v_{ES}}\times\left(\frac{D_{p}\left(t_{D}\right)}{D_{p}\left(0\right)}\right)^{3}}{\frac{M_{s}}{\rho_{s}}\left(p_{s}-p_{s}*\right)\times\frac{D_{sa}}{RT\delta_{a}}\times\frac{p_{a}}{p_{s}}\ln\left(\frac{p_{a}}{p_{a}-p_{s}}\right)}$ (S11)

3.3 Polymerization reaction

(1) Background

A mathematical model was proposed in previous studies [27, 29] for the formation of a thin-film membrane under non-steady-state conditions. In this model, the two-dimensional surface of the film is assumed to be infinite. The concentrations of reactants and products during interfacial polymerization are described by the following partial differential equation.

$\frac{\partial N_{i}}{\partial t}=D_{i}\frac{\partial^{2}N_{i}}{\partial x^{2}}+r_{i}\left(N_{i}\right)$ (S12)

Where, $D_{i}\frac{\partial^{2}N_{i}}{\partial x^{2}}$ is the diffusion term; $r_{i}\left(N_{i}\right)$ is the reaction term; *x* is the distance from the interface; *t* is the time elapsed from the moment the two solutions are brought into contact.

Conventional interfacial polymerization includes coupled processes, such as the diffusion of MPD and TMC, the reaction between MPD and TMC monomers, the reaction between MPD monomers and unreactive acyl chloride group of PA layers, the reaction between TMC monomers and unreactive amine group of PA layers, and the reaction between unreactive amine group and acyl chloride group in PA layers. Based on these reactions, Equation (S12) can be extended to the following equations^[27]^:

$\begin{matrix} \frac{\partial N_{MPD}}{\partial t}=\frac{\partial}{\partial x}\left(D_{MPD}\frac{\partial N_{MPD}}{\partial x}\right)-2kN_{MPD}\left(3N_{TMC}+N_{O=C-Cl}\right) \\ \frac{\partial N_{TMC}}{\partial t}=\frac{\partial}{\partial x}\left(D_{TMC}\left(\frac{\partial N_{TMC}}{\partial x}+\frac{N_{TMC}}{1-N_{PA}}\frac{\partial N_{PA}}{\partial x}\right)\right)-3kN_{TMC}\left(2N_{MPD}+N_{-NH2}\right) \\ \frac{\partial N_{PA}}{\partial t}=6kN_{MPD}N_{TMC}+2kN_{MPD}N_{O=C-Cl}+3kN_{TMC}N_{-NH2}+kN_{-NH2}N_{O=C-Cl} \\ \frac{\partial N_{-NH2}}{\partial t}=2kN_{MPD}\left(3N_{TMC}+N_{O=C-Cl}\right)-3kN_{TMC}N_{-NH2}-kN_{-NH2}N_{O=C-Cl} \\ \frac{\partial N_{O=C-Cl}}{\partial t}=6kN_{TMC}\left(2N_{MPD}+N_{-NH2}\right)-2kN_{MPD}N_{O=C-Cl}-kN_{-NH2}N_{O=C-Cl} \end{matrix}$ (S13)

where, *N_MPD_* is the molar concentration of MPD molecules; *N_TMC_* is the molar concentration of TMC molecules; *N_PA_* is the molar concentration of amide bonds; *N_-NH2_* is the molar concentration of unreacted amine group; *N_O=C-Cl_* is the molar concentration of unreacted acyl chloride group; *D_MPD_* is the diffusion coefficient of MPD molecules; *D_TMC_* is the diffusion coefficient of TMC molecules; *k* is the second order reaction rate constant of amine and acyl chloride groups.

The diffusion coefficient (*D_i_*) of MPD and TMC molecules is related to the volume fraction of polymers in PA layer:

$D_{i}=D_{i,0}\left(1-\frac{V_{PA}}{V_{PA}^{\max}}\right)^{\alpha}=D_{i,0}\left(1-\frac{k_{V}N_{PA}}{k_{V}N_{PA}^{\max}}\right)^{\alpha}=D_{i,0}\left(1-\frac{N_{PA}}{N_{PA}^{\max}}\right)^{\alpha}$ (S14)

where, *D_i,_*_0_ is the diffusion coefficient of MPD and TMC in the pure solvent solution; *V_PA_* is volume fraction of polymers in PA layer; *V_PA_^max^* is maximum volume fraction of polymers in PA layer; α is diffusion hindrance factor; *k_V_* is the molar volume of polymers in PA layer, which is 70 cm^3^/mol in this study; *N_PA_^max^* is maximum molar concentration of amide bonds.

An accurate solution of this model requires sufficient computing power and a long solution time. To save computer power and calculation time, the above numerical model was simplified based on some assumptions.

(2) Kinetics applied in this study

For the 3P membranes, their PA layer can be divided into an incipient film and several growth layers. As both the incipient film and growth layers are particularly thin, the concentrations of the reactants and products in each ultrathin layer can be assumed to be constant along the thickness direction. Therefore, the partial differential equation describing the formation of each ultrathin layer can be simplified to an ordinary differential equation.

For the preparation of the incipient film, MPD droplets are deposited on the substrate before TMC droplets. Therefore, at the early stage of incipient film formation, the MPD supply is sufficient, resulting in a homogeneous reaction between MPD and TMC. At the final stage of incipient film formation, the MPD supply is limited by its diffusion through the formed ultrathin film between MPD and TMC microdroplets. This results in a diffusion-limited reaction between MPD and the unreacted acyl chloride groups.

For the preparation of the growth layers, Needle #1 extrudes MPD organic solution, while Needle #2 simultaneously extrudes TMC organic solution. These two solutions were mixed in the Taylor-cone, and the MPD and TMC monomers in the mixed organic solutions underwent homogeneous polymerization to produce oligomers. These oligomers then deposit on the generated network of the incipient film. The residual MPD monomers in the aqueous phase pass through the channels of the incipient film and enter the organic phase to react with the oligomers. Thus, in the Taylor-cone, there is a homogeneous polymerization between MPD and TMC. Then, on the surface of formed PA layer, the MPD supply is limited by its diffusion through PA membrane, resulting in a diffusion-limited reaction between free MPD and the unreacted acyl chloride groups (named surface polymerization).

Therefore, at the early stage for polymerization reaction of each layer, MPD supply is sufficient, and the consumption of acyl chloride follows the second-order reaction kinetics:

$\frac{dN_{O=C-Cl}}{dt}=-2kN_{MPD}N_{O=C-Cl}$ (S15)

where, *N_O=C-Cl_* is the concentration of acyl chloride groups (both in TMC and PA), mol/L; *N_O=C-Cl_*^0^ is the initial concentration of acyl chloride groups in the organic phase, mol/L; *N_MPD_*^0^ is the initial concentration of MPD in the organic phase, mol/L; *k* is the second-order reaction kinetic constant.

Since the consumption of MPD and TMC was very small, and the concentration of MPD can be expressed as a multiple of the concentration of TMC, that is, *N_MPD_* = *cN_TMC_*. Therefore, Equation (S15) was solved as Equation (S16):

$\begin{matrix} N_{O=C-Cl}=N_{O=C-Cl}^{0}\frac{1}{2kcN_{O=C-Cl}^{0}t+1} \\ =N_{O=C-Cl}^{0}\frac{1}{2kN_{MPD}^{0}t+1} \end{matrix}$ (S16)

When the hydrolysis of acyl chloride is ignored, the amount of amide bond produced is equal to the consumption of acyl chloride:

$\begin{matrix} N_{PA}=N_{O=C-Cl}^{0}-N_{O=C-Cl} \\ =N_{O=C-Cl}^{0}-N_{O=C-Cl}^{0}\frac{1}{2kcN_{O=C-Cl}^{0}t+1} \\ =N_{O=C-Cl}^{0}\frac{2kN_{MPD}^{0}t}{2kN_{MPD}^{0}t+1} \end{matrix}$ (S17)

where *N_PA_* is the concentration of amide group in the reaction region.

We assumed that the deposited TMC molecules are completely consumed; the initial concentration of acyl chloride groups is equal to the maximum molar concentration of amide bonds. Therefore, Equation (S18) is obtained.

$N_{PA}=N_{PA}^{\max}\frac{2kN_{MPD}^{0}t}{2kN_{MPD}^{0}t+1}$ (S18)

where *N_PA_^max^* is the maximum concentration of amide group in the reaction region.

At the final stage of each layer formation, the supply of MPD molecules is limited by their diffusion. Thus, the diffusion rate of MPD is equal to the consumption rate of MPD in polymerization reaction and the amount of amide bond production.

$\frac{dN_{PA}}{dt}=2kN_{MPD}^{W-O}N_{O=C-Cl}$ (S19)

$2\frac{1}{l_{i}}\left(D\frac{N_{MPD}^{W-O}}{l_{D}}\right)=2kN_{MPD}^{W-O}N_{O=C-Cl}$ (S20)

where *N_MPD_^W-O^* is the molar concentrations of MPD that diffused into organic phase from aqueous phase; *k* is the reaction rate constant of polymerization reaction. *D* is the diffusion coefficient of MPD in PA; *l_D_* is the thickness of diffusion layer, 14 nm in this study; *l_i_* is the design thickness of layer i.

By substituting Equation (S14) and (S19) into (S20), Equation (S21) is obtained:

$\frac{dN_{PA}}{dt}=\frac{2N_{MPD}^{W-O}}{l_{D}l_{i}}D_{MPD,0}\left(1-\frac{k_{V}}{V_{PA}^{\max}}N_{PA}\right)^{\alpha}$ (S21)

After integration, Equation (S22) is obtained:

$N_{PA}=N_{PA}^{\max}\left\{1-\left[\left(\alpha-1\right)\frac{1}{N_{PA}^{\max}}\left(\frac{2D_{MPD,0}N_{MPD}^{W-O}}{l_{D}l_{i}}t+C_{2}\right)\right]^{\frac{1}{1-\alpha}}\right\}$ (S22)

To calculate the amount of amide bond produced during the transition stage, a Sigmoid function was used to give the concentration of MPD molecules in the polymerization reaction.

For the preparation of the incipient film, Equation (S23) is used.

$\left\{\begin{matrix} \sigma\left(t\right)=\frac{1}{1+e^{-\ln\left(t/t_{T}\right)}}=\frac{t}{t+t_{T}} \\ \sigma'\left(t\right)=1-\sigma\left(t\right)=1-\frac{t}{t+t_{T}} \end{matrix}\right.$ (S23)

where, *t* is the reaction time, s; *t_T_* is the threshold time for entering the diffusion-limited growth stage, 0.3 s in this study.

For the preparation of the growth layers, there are two parts of MPD monomers. One part was the MPD monomers that diffused through the incipient film from aqueous solution. The other was the MPD monomers that dissolved in organic solution. Therefore, taking this influence into account, Equation (S24) is used.

$\left\{\begin{matrix} \sigma\left(t\right)=\frac{N_{MPD}^{W-O}}{N_{MPD}^{W-O}+N_{MPD}^{O}}\times\frac{1}{1+e^{-\ln\left(t/t_{T}\right)}}=\frac{N_{MPD}^{W-O}}{N_{MPD}^{W-O}+N_{MPD}^{O}}\times\frac{t}{t+t_{T}} \\ \sigma'\left(t\right)=1-\sigma\left(t\right)=1-\frac{N_{MPD}^{W-O}}{N_{MPD}^{W-O}+N_{MPD}^{O}}\times\frac{t}{t+t_{T}} \end{matrix}\right.$ (S24)

where, *N_MPD_^W-O^* is the molar concentration of MPD monomers that diffused into organic phase from aqueous phase; *N_MPD_^O^* is the molar concentration of MPD monomers that dissolved in organic phase when preparing growth layers.

Finally, for the fabrication of both the incipient film and growth layers, the amide bond concentrations can be calculated using Equation (S25):

$N_{PA}=N_{PA}^{\max}\frac{2kN_{MPD}^{O}t}{2kN_{MPD}^{O}t+1}\sigma'\left(t\right)+N_{PA}^{\max}\left\{1-\left[\left(\alpha-1\right)\frac{1}{N_{PA}^{\max}}\left(\frac{2D_{MPD,0}N_{MPD}^{W-O}}{l_{D}l_{i}}t+C_{2}\right)\right]^{\frac{1}{1-\alpha}}\right\}\sigma\left(t\right)$ (S25)

where, *N_MPD_*^0^ is the initial concentration of MPD monomers in homogeneous reaction occurs. In the preparation of the incipient film, it equals the concentrations of MPD monomers that diffused into organic phase from aqueous phase (*N_MPD_^W-O^*). When printing the growth layers, it equals the concentrations of MPD monomers that dissolved in organic phase (*N_MPD_^O^*).

An equation was used to estimate *N_MPD_^W-O^*, which considers the partition of MPD between aqueous solution, organic solution and formed PA layer.

$N_{MPD}^{W-O}=N_{MPD}^{W}P_{\text{interface}}P_{PA}=N_{MPD}^{W}P_{\text{interface}}\left(1-\sum_{1}^{i-1} \frac{N_{PA}^{x}l_{x}}{N_{PA}^{\max}l_{D}}\right)$ (S26)

where, *N_MPD_^W^* is the molar concentration of MPD monomers that dissolved in aqueous solution; *P_interface_* is the partition coefficient when MPD diffused through water-oil interface, which is 0.03 in this work; *P_PA_* is the partition coefficient of MPD between formed PA layer and organic solution; *N_PA_^x^* is the concentration of amide bond for formed PA layer x; *l_x_* is the thickness of layer x.

Note S4 Application of the numerical prediction model

The developed numerical prediction model contains 5 parameters: second-order reaction constant (*k*), maximum amide bond content (*N_PA_^max^*), diffusion coefficient of MPD in the pure solvent (*D_MPD_*_,0_), bending factor (α) and the constant (*C*_2_). If the values of these parameters are known in advance, the numerical prediction model can be used to calculate the amide bond content of PA layers. Additionally, the numerical prediction model can further be used to predict the crosslinking degree and thickness of PA layers since the amide bond content is related to these features of the film properties in the 3P membranes.

4.1 Parameters estimation for the numerical prediction model

In this work, the values of these 5 parameters (*k*, *N_PA_^max^*, *D_MPD_*_,0_, α and *C*_2_) were estimated by a nonlinear fitting method with the amide bond content of the incipient film.

According to previous studies^[36]^, interfacial polymerization is described by the following equation:


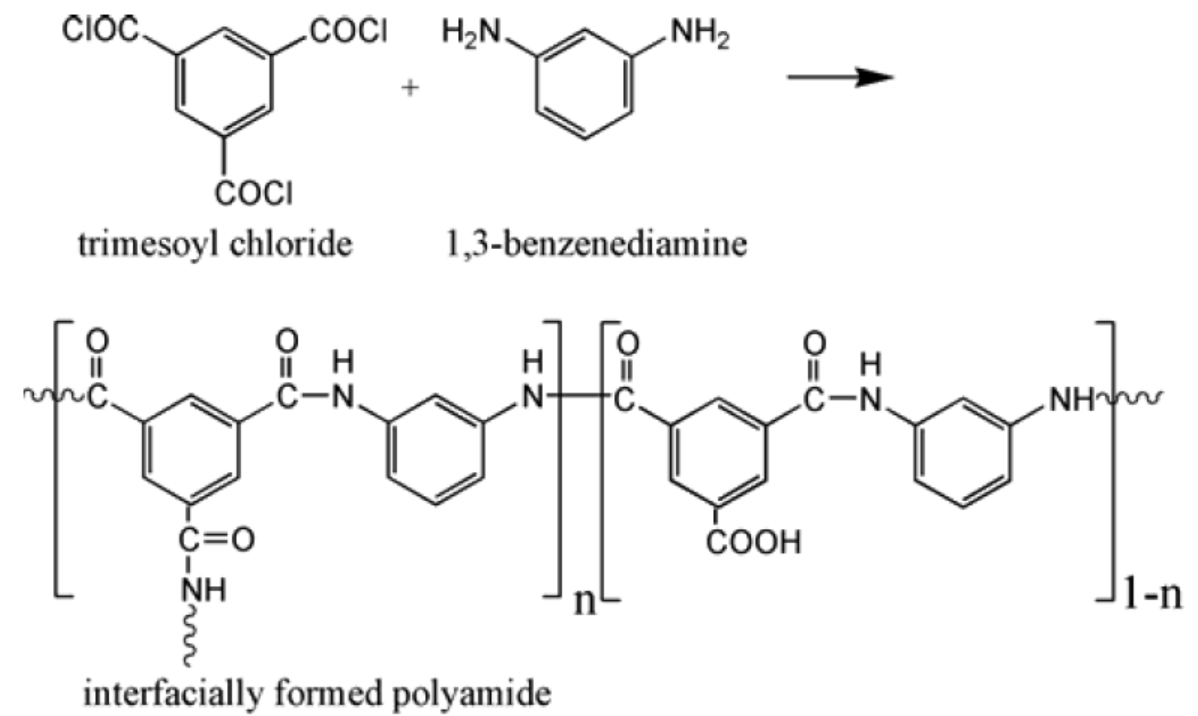
 (S27)

where n is the percentage of crosslinked units, that is, the crosslinking degree (*DNC*); 1-n is the percentage of linear units.

Based on this equation, the *DNC* is calculated as follows:

$DNC=\frac{N_{PA}}{N_{TMC}}-2$ (S28)

$N_{TMC}=\frac{\sigma_{TMC,g}}{l_{PA}M_{TMC}}$ (S29)

where, σ*_TMC,g_* is the deposition amount of TMC, g/mm^2^; *l_PA_* is the thickness of PA layer, nm.

For electrospray, the deposition amount of TMC monomers is related to the injection rate of TMC solutions, the concentration of TMC monomers, the moving speed of nozzle and the distance between two adjacent printing paths. Equation (S30) is used to calculate the deposition amount of TMC monomers.

$\sigma_{TMC}=\frac{c\left(TMC\right)\times Q_{T}}{v_{ES}\times d_{ES}}$ (S30)

where *c(TMC)* is the concentration of TMC solution, g/mL; *Q_T_* is the flowrate of TMC solutions injected by the syringe pump, mL/s; *v_ES_* is the moving speed of needles, mm/s; *d_ES_* is the spacing of printing paths, mm.

By substituting Equation (S29) and (S30) into (S28), Equation (S31) is obtained:

$N_{PA}=\frac{c\left(TMC\right)Q_{T}\left(DNC+2\right)}{v_{ES}d_{ES}M_{TMC}l_{PA}}$ (S31)

The XPS and AFM analysis of PA layers can provide the values of *DNC*, *l_PA_* and *N_PA_*. Specifically, we prepared the five incipient films using MPD aqueous solution and five TMC organic solvents (n-hexane, n-heptane, n-octane, n-nonane and n-decane, respectively). With the XPS and AFM analysis, the measured values of DNC and *l_PA_* were obtained for the five incipient films. Then, the values of *N*_PA_ were calculated using Equation (S31), and the results are shown in Table S3.

As the mechanism behind ES-IP is attributed to the polymerization between MPD and TMC induced by the interface between two immiscible microdroplets, the reaction time of interfacial polymerization for preparing the incipient film is assumed to be the volatilization time of organic solvent. Therefore, Equation (S11) is used to calculate the reaction time of interfacial polymerization for the incipient film, and the results are shown in Table S3.

By substituting the values of *N_PA_* and reaction time into Equation (S25), the following five equations can be obtained, with each for one type of organic solvent. These equations all contain 5 identical parameters, which are second-order reaction constant (*k*), maximum amide bond content (*N_PA_^max^*), diffusion coefficient of MPD in the pure solvent (*D_MPD_*_,0_), bending factor (α) and the constant (*C*_2_), respectively. Based on these 5 equations, nonlinear fitting was conducted and the values for the 5 parameters were obtained. The results are shown in Table S4. As the correlation coefficient (R^2^) is 0.999, the values of these 5 parameters can be used to predict the crosslinking degree and thickness of PA layers with the numerical prediction model.

4.2 Predicting the crosslinking degree and thickness of PA layers with the numerical prediction model

With the values of the parameters (*k*, *N_PA_^max^*, *D_MPD_*_,0_, α and *C*_2_) listed in Table S4, the numerical prediction model can be used to predict the crosslinking degree and thickness of PA layers in various 3P membranes using the following equations.

$DNC=\frac{3N_{PA}}{N_{PA}^{\max}}-2$ (S32)

$l_{PA}=\frac{c\left(TMC\right)Q_{T}\left(DNC+2\right)}{v_{ES}d_{ES}M_{TMC}N_{PA}}$ (S33)

Equation (S32) and (S33) are derived from Equation (S28) and (S31), respectively. By substituting the N_PA_ obtained from the numerical prediction model into Equation (S32), the *DNC* of PA layers is calculated. Then, the *DNC* and *N_PA_* are used to calculate the *l_PA_* with Equation (S33).

In this study, we designed common and special 3P membranes and fabricated them with the PE3DP. The printing parameters and detailed regulation strategy are shown in Note S2 and Table S2, respectively. We used the numerical prediction model to predict the amide bond content of the incipient film and growth layers in these two membranes. Then, with the predicted amide bond contents, we calculated the crosslinking degree and thickness of the incipient film and growth layers using Equation (S32) and Equation (S33). The results are shown in Table S5. The XPS and AFM analysis were conducted for the two membranes, and the results are also shown in Table S5. The difference between the predicted and measured values is less than 10%. This indicates that the numerical prediction model is applicable and can be used for the operation of the PE3DP.

Note S5 Characterization and analysis methods

5.1 Method for separating PA layer from UF membrane

For the analysis of AFM, XPS, FTIR and PTIR, it is necessary to transfer PA layers to the Si wafer, avoiding the interference of UF membrane. Figure S7 shows a schematic of the method for transferring PA layers to the Si wafer. Specifically, A TFC-PA membrane was first attached onto the Si wafer. Then, dimethylformamide (DMF) was dripped onto the back cover of the membrane, and the UF membrane was dissolved by DMF. Thus, the non-woven fabrics were separated with PA layer. Finally, the separated PA layer was washed with DMF for 10 mins, air dried at room temperature for 24 h, and stored for characterization.

5.2 Scanning electron microscopy (SEM)

A scanning electron microscopy (SEM, Hitachi S-4700, Japan) was used to image the top surface of the polyamide layer. For imaging surface morphology, the samples of the membrane were dried, attached to the SEM stage, and sputter coated with a thin layer of gold (Au) and platinum (Pt) under vacuum (0.6 torrs). A coating time of 100 seconds with 20 mA current was selected, which adds ~10−20 nm of coating. After coating, membranes were imaged in the SEM using a 3-4 kV accelerating voltage, a working distance of 5-10 mm, and the ETD detector using SE mode.

5.3 Atomic force microscope (AFM)

An atomic force microscope (AFM, Dimension Icon, Bruker, USA) was used to analyze the thickness of PA layers, the intrinsic roughness of PA layers and the surface roughness of 3P membrane. The thickness and the intrinsic roughness of PA layers were measured with the samples that were attached to the silicon wafer (Figure S8) ^[54]^. The surface roughness of 3P membranes was measured directly without any further pretreatment.

Initially, a 20 μm × 20 μm area was imaged at 1 Hz to ensure that there was no unusual obstruction that could damage the probe tip. Next, 10 μm × 10 μm images were acquired at line rates of 0.5 Hz, using intermittent-contact (also known as AC) mode with typical feedback gain settings to optimize surface tracking. The low line rate was applied to best extract the true surface topography. Finally, the AFM images were analyzed by NanoScope Analysis software (NanoScope Analysis 3.0, Burker). When the Si wafer region in the images was treated by the “Plane fit” tool of NanoScope software, the boundary between the PA layer and Si wafer was clearly visible. Then, the height statistical histogram of images shows two distinct peaks. The difference in height between the two peaks can represent the thickness of the PA layer. The “Roughness” tool was used to calculate the average roughness (Ra), root mean square roughness (Rq) and surface area difference (SAD) of PA layers and 3P membrane. The Ra represents the whole roughness of PA layers. The Rq was more sensitive to the extreme values of the thickness, and it can reflect the bulges that generated by the intrusion of PA layer into UF membrane pores in this study. SAD shows the increase of surface area caused by the roughness of PA layers. These results are tabulated in Table S6.

5.4 X-ray photoelectron spectroscopy (XPS)

XPS spectroscopy was performed on PA membranes using a Multifunctional X-ray Photoelectron Spectroscope (Escalab 250XI, Thermo Scientific, Japan) with aluminum Kα radiation as X-ray source (1486.6 eV).

Survey XPS spectra were obtained by sweeping over 0–1350 eV electron binding energy with a resolution of 1 eV. Each survey spectra were the average of five survey scans. High resolution scans were obtained by an average of 100 scans with a resolution of 0.1 eV for C 1s peak, N 1s peak and O 1s peak, respectively. For all scans, a spot size of 650 μm was used. Sample charging was minimized by an electron flood gun operating at 3 eV. The high-resolution XPS spectra were subtracted by the Shirley-type background, and Gaussian-Lorentz peak deconvolution was performed to estimate the binding energy shift (δ_BE_) of carbon 1s. For each membrane type, the deconvoluted peaks were normalized against the peak at the lowest binding energy (corrected to 284.8 eV).

For the analysis of XPS spectra, the deconvolved C1s XPS narrow scan of PA layers can be divided into hydrocarbon groups (C*-C and C*-H) at 284.8 eV, amide and ether groups (C*-N, C*-O-C) at 285.8 eV, and carbonyl groups (O*=C-O and O*=C-N) at 287.8 eV. The N1s XPS narrow scan of both membranes showed amide group (O=C-N*) at 399.7 eV without any other groups. The deconvolved O1s XPS narrow scan of PA layers can be divided into carbonyl group (O*=C-O and O*=C-N) at 531.0 eV, sulfonic group (O*=S=O) at 531.8 eV, carbon-oxygen single bond (C-O*) at 532.5 eV, and water (H_2_O*) at 533.2 eV.

According to previous studies^[36]^, the crosslinking degree (DNC) can be calculated using the data obtained from the XPS analysis, and the calculation equation is as follows:

$DNC=\frac{4-\frac{C_{O*=C}+C_{O*-C}}{C_{N}}\times2}{1+\frac{C_{O*=C}+C_{O*-C}}{C_{N}}}\times100\%$ (S34)

Where C_N_ is the atomic percentage of elemental nitrogen; C_O*=C_ is the atomic percentage of oxygen element in O=C bond; C_O*-C_ is the atomic percentage of oxygen element in O-C bond.

5.5 Angle-resolved X-ray photoelectron spectroscopy (ARXPS)

According to previous studies^[38, 55]^, angle-resolved X-ray photoelectron spectroscopy (ARXPS) is one method to evaluate atomic concentrations with depth for polymers. Therefore, we used ARXPS to evaluate atomic concentrations with depth for the incipient film and growth layers in this work.

First, the 3P membranes were subjected to the ARXPS analysis with the X-ray irradiated from the surface (labeled as front side). Then the PA layer of the 3P membranes was transferred onto the Si wafer and subjected to the ARXPS analysis with the X-ray irradiated from the side attached to the UF membranes (labeled as back side). For the front side and back side, both the survey spectra and the narrow spectra (for C 1s, N 1s and O 1s) were obtained at photoemission angles of 0°, 35° and 70°.

For the 4-layers 3P membrane, the photoelectron intensity *I_i_^f^* of element *i* at the front side is:

$I_{i}^{f}=\Phi TA\sigma\left(\begin{matrix} \int_{0}^{d_{4}} C_{i,4}e^{-z/\left(\lambda_{i}\cos\theta\right)}dz \\ +\int_{0}^{d_{3}} C_{i,3}e^{-d_{4}/\left(\lambda_{i}\cos\theta\right)}e^{-z/\left(\lambda_{i}\cos\theta\right)}dz \\ +\int_{0}^{d_{2}} C_{i,2}e^{-\left(d_{3}+d_{4}\right)/\left(\lambda_{i}\cos\theta\right)}e^{-z/\left(\lambda_{i}\cos\theta\right)}dz \\ +\int_{0}^{d_{1}} C_{i,1}e^{-\left(d_{2}+d_{3}+d_{4}\right)/\left(\lambda_{i}\cos\theta\right)}e^{-z/\left(\lambda_{i}\cos\theta\right)}dz \end{matrix}\right)$ (S35)

The photoelectron intensity *I_i_^b^* of element *i* at the back side is:

$I_{i}^{b}=\Phi TA\sigma\left(\begin{matrix} \int_{0}^{d_{1}} C_{i,1}e^{-z/\left(\lambda_{i}\cos\theta\right)}dz \\ +\int_{0}^{d_{2}} C_{i,2}e^{-d_{1}/\left(\lambda_{i}\cos\theta\right)}e^{-z/\left(\lambda_{i}\cos\theta\right)}dz \\ +\int_{0}^{d_{3}} C_{i,3}e^{-\left(d_{1}+d_{2}\right)/\left(\lambda_{i}\cos\theta\right)}e^{-z/\left(\lambda_{i}\cos\theta\right)}dz \\ +\int_{0}^{d_{4}} C_{i,4}e^{-\left(d_{1}+d_{2}+d_{3}\right)/\left(\lambda_{i}\cos\theta\right)}e^{-z/\left(\lambda_{i}\cos\theta\right)}dz \end{matrix}\right)$ (S36)

where Φ is the x-ray flux; *T* is the transmission/detector efficiency; *A* is the analysis area; σ is the cross-section for photoelectron emission; *t* is the thickness of the layer; *C_i,j_* is the atomic percentage of the element in question in the layer; *z* is the depth; λ*_i_* is the photoelectron inelastic mean free path through the layer; θ is the photoemission angle relative to the normal to the sample surface.

Evaluating the integrals, Equation (S47) and (S48) are obtained：

$I_{i}^{f}=\Phi TA\sigma\lambda_{i}\cos\theta\left(\begin{matrix} C_{i,4}\left(1-e^{-d_{4}/\left(\lambda_{i}\cos\theta\right)}\right) \\ +C_{i,3}e^{-d_{4}/\left(\lambda_{i}\cos\theta\right)}\left(1-e^{-d_{3}/\left(\lambda_{i}\cos\theta\right)}\right) \\ +C_{i,2}e^{-\left(d_{3}+d_{4}\right)/\left(\lambda_{i}\cos\theta\right)}\left(1-e^{-d_{2}/\left(\lambda_{i}\cos\theta\right)}\right) \\ +C_{i,1}e^{-\left(d_{2}+d_{3}+d_{4}\right)/\left(\lambda_{i}\cos\theta\right)}\left(1-e^{-d_{1}/\left(\lambda_{i}\cos\theta\right)}\right) \end{matrix}\right)$ (S37)

$I_{i}^{b}=\Phi TA\sigma\lambda_{i}\cos\theta\left(\begin{matrix} C_{i,1}\left(1-e^{-d_{1}/\left(\lambda_{i}\cos\theta\right)}\right) \\ +C_{i,2}e^{-d_{1}/\left(\lambda_{i}\cos\theta\right)}\left(1-e^{-d_{2}/\left(\lambda_{i}\cos\theta\right)}\right) \\ +C_{i,3}e^{-\left(d_{1}+d_{2}\right)/\left(\lambda_{i}\cos\theta\right)}\left(1-e^{-d_{3}/\left(\lambda_{i}\cos\theta\right)}\right) \\ +C_{i,4}e^{-\left(d_{1}+d_{2}+d_{3}\right)/\left(\lambda_{i}\cos\theta\right)}\left(1-e^{-d_{4}/\left(\lambda_{i}\cos\theta\right)}\right) \end{matrix}\right)$ (S38)

The photoelectron intensity of element *i* (*I_i_*) is simplistically written as:

$I_{i}=\Phi TA\sigma\lambda_{i}\cos\theta\sum_{j=1}^{n} C_{i,j}\alpha_{i,j}\kappa_{i,j}$ (39)

where α*_i,j_* is the attenuation coefficient of element *i* in layer *j*; κ*_i,j_* is the integral coefficient of element *i* in layer *j*.

Therefore, the atomic percentage of element at angle θ (C*_i,_*_θ_) is:

$C_{i,\theta}=\frac{\frac{I_{i}}{\Phi TA\sigma}}{\sum_{i} \frac{I_{i}}{\Phi TA\sigma}}=\frac{\lambda_{i}\cos\theta\sum_{j=1}^{n} C_{i,j}\alpha_{i,j}\kappa_{i,j}}{\sum_{i} \left(\lambda_{i}\cos\theta\sum_{j=1}^{n} C_{i,j}\alpha_{i,j}\kappa_{i,j}\right)}$ (S40)

For element C, N and O, their inelastic mean free paths (λ*_i_*) are similar in PA layer^[56]^, resulting in similar α and κ. The distribution of the three elements in PA layer is also relatively uniform. Therefore, the denominator could be simplified as follows:

$\begin{matrix} \sum_{i} \left(\lambda_{i}\cos\theta\sum_{j=1}^{n} C_{i,j}\alpha_{i,j}\kappa_{i,j}\right)\approx\lambda\cos\theta\sum_{i} \left(\sum_{j=1}^{n} C_{i,j}\alpha_{i,j}\kappa_{i,j}\right) \\ \approx\lambda\cos\theta\sum_{i} C_{i}\times\sum_{j=1}^{n} \alpha_{i,j}\kappa_{i,j} \\ =\lambda\cos\theta\sum_{j=1}^{n} \alpha_{i,j}\kappa_{i,j} \end{matrix}$ (S41)

Then, Equation (S40) can be simplified into Equation (S42):

$C_{i,\theta}\approx\frac{\sum_{j=1}^{n} C_{i,j}\alpha_{i,j}\kappa_{i,j}}{\sum_{j=1}^{n} \alpha_{i,j}\kappa_{i,j}}$ (S42)

With the results of the ARXPS, the element content of each PA layer could be obtained by nonlinear fitting of C*_i,_*_θ_ using the Microsoft Excel Solver and used for calculating the crosslinking degree of PA layers with Equation (S34).

5.6 Attenuated total reflection - Fourier transform infrared spectrum (ATR-FTIR)

ATR-FTIR spectra were obtained using a Nicolet iS10 FTIR spectrometer (Thermo Scientific, MA, USA) equipped with a QuestATR element (45º Diamond Crystal, SPECAC, UK) and an Omnic software (Omnic 7.3, Thermo Electron Corporation). The PA layer was first separated from the substrate and then pressed tightly against the crystal plate. Carbon dioxide and water vapor were removed during measurements. ATR spectra were corrected at 1800 cm^–1^ and 800 cm^–1^ by the Auto baseline tool. The penetration depth at this wavelength coverage is approximately 500 nm, which is much higher than the thickness of PA layers. Therefore, ATR-FTIR spectra can provide data about chemical structures throughout the whole PA layer of the 3P membranes.

5.7 Photothermal-induced resonance (PTIR)

Atomic force microscopy-infrared spectroscopy (AFM-IR), named in some publications as photothermal-induced resonance (PTIR), is a scanning probe technique where a pulsed, tunable, IR laser is added to an AFM instrument, resulting in nanoscale IR molecular chemical information^[57-58]^. Anasys nanoIR3 PTIR instrument (Burker, MA, USA) interfaced with a tunable pulsed laser was used to collect PTIR spectra and images with wavelength tunable from 3600 cm^−1^ to 2700 cm^−1^, and from 1800 cm^−1^ to 800 cm^−1^. The laser was focused on the sample to a ≈50 μm spot size and the samples were illuminated from the top of the side at a spectral resolution of 2 cm^−1^. AFM-IR data were obtained in tapping-mode with commercially available gold-coated silicon AFM probes. The PTIR images were labelled with the laser wavelength used in the experiments, typically close but not always corresponding exactly to the IR peak maximum. All PTIR, phase and topography images were obtained at 0.5 Hz scan rate, with a 40 nm pixel size in both the horizontal and vertical directions. Morphological and infrared spectra, as well as chemical imaging, were processed using Analysis studio software (Analysis Studio v3.17, Burker).

Similar peaks can be found in PTIR spectra when compared with the ATR-FTIR spectra. That means the peaks in PTIR spectra at 1656, 1542, 1700 and 1600 cm^−1^ can also be assigned to amide I band, amide II band, carboxyl groups and amine groups, respectively^[36]^. Therefore, we carried out surface scanning of the incipient film and PA layer of 3P membrane to obtain the arctan value of A_1700_/A_1656_ and A_1600_/A_1656_, respectively. The arctan value of A_1700_/A_1656_ represents the relative amount of carboxyl groups to amide groups, while the arctan value of A_1600_/A_1656_ represents the relative amount of amine groups to amide groups, respectively.

5.8 Scanning transmission electron microscopy (STEM)

A scanning transmission electron microscopy (STEM) (JEM-ARM300F, JEOL, Japan) was used to image the cross section of 3P membrane in the high-angle annular dark-field (HAADF) mode. The acceleration voltage of STEM was 300 kV. A camera length of 125 mm was used.

Firstly, we prepared 40 μM and 1 μM AgNO_3_ solution for the silver binding and surface washing steps, respectively^[59]^. 0.1 M NaOH and HCl solution were used to adjust the pH of the binding and washing solution. Secondly, the samples of the 3P membranes were wetted in DI water for 30 mins, cut into 2 cm × 1 cm (length × width), and immersed in 10 mL of binding solution (40 μM AgNO_3_) for 10 min. Then, the membranes were carefully transferred to 10 mL of washing solution (1 μM AgNO_3_) for 7 min to rinse off the unbound Ag^+^. After the rinsing step was repeated four times, the membranes were blotted dry. Finally, the dried membranes were embedded, sliced and imaged with the STEM. At a magnification of 8 million times, the bound silver ions and the organic PA layer form a significant contrast. Therefore, the adsorption site of the silver ions, carboxyl group, could be well located.

5.9 Grazing incidence small angle X-ray scattering (GISAXS)

A grazing incidence small angle X-ray scattering (GISAXS) (Xeuss 2.0, Xenocs, France) was used to analyze the pore size distributions for PA layer of 3P membranes^[60-62]^. The PA layers were separated from UF membrane and fixed on Si wafer before the test. The monochromatized X-ray wavelength was λ = 0.154189 nm. A detector (Pilatus 3R 300K) was used for GISAXS experiments where the distance between the sample and the detector was 15.0 cm. The data acquisition time for each GISAXS pattern was 300 s. Due to the curvature of Ewald sphere, a missing wedge appears when the scattering pattern is projected on 𝑞𝑟−𝑞𝑧 plane (Figure S21a&b). These data were then analyzed with Fit2D software (Fit2D V18.002, Andy Hammersley) to obtain the pore size distributions (Figure S21c).

5.11 Separation performance test

Separation performance of the membranes was investigated by laboratory-scale cross-flow flat membrane equipment with an effective area of 20.0 cm^2^ at room temperature^[63]^. All reverse osmosis (RO) tests were performed at an operation pressure of 15.5 bar. NaCl solutions (2.0 g/L) were used as feed solutions with the pH values were adjusted to neutral. The membranes had been compacted at 20.0 bar for 2 h before tests to obtain a stable flux.

The pure water flux of the membranes (*J_w_*, L/m^2^ h) was calculated with the following equation:

$J_{w}=\frac{V_{w}}{A_{m}\Delta t}$ (S43)

where *V_w_* (L) is the volume of permeated water; *A_m_* (m^2^) is the effective area of the PA membranes; Δ*t* (h) is the filtration time.

The salt rejection *R* (%) was calculated according to the following equation:

$R=\left(1-\frac{C_{p}}{C_{f}}\right)\times100\%$ (S44)

where *C_p_* (g/L) and *C_f_* (g/L) are the NaCl concentrations of the permeated solutions and feed solutions, respectively.

The concentrations of NaCl solutions were measured by electrical conductivity (DDSJ-308A, Leici, China). At least three membrane samples were used for all separation performance measurements.

Water permeability (A) was calculated using the following equation:

$A=\frac{J_{w}}{\Delta P}$ (S45)

Solute permeability (𝐵) was calculated using the following equation^[64]^:

$B=J_{w}\left(\frac{1-R}{R}\right)\exp\left(-\frac{J_{w}}{k_{mt}}\right)$ (S46)

where, 𝑘_𝑚𝑡_ is the mass transfer coefficient for the cross-flow system. It can be calculated by following equations^[65-66]^:

$Sh=1.85\left(\mathrm{Re}Sc\frac{d_{h}}{L}\right)^{0.33}$ (S47)

$Sh=\frac{k_{mt}L}{D_{AB}}$ (S48)

Here, *Re* is the Reynolds number; 𝑆ℎ is the Sherwood number; 𝐿 is the characteristic length; *D*_AB_ is the diffusion coefficient of NaCl in water. The resulting mass transfer coefficient calculated for the cross-flow system was found to be 3.32 × 10^−5^ m/s. Then, the water/salt permselectivity (A/B) can be obtained.


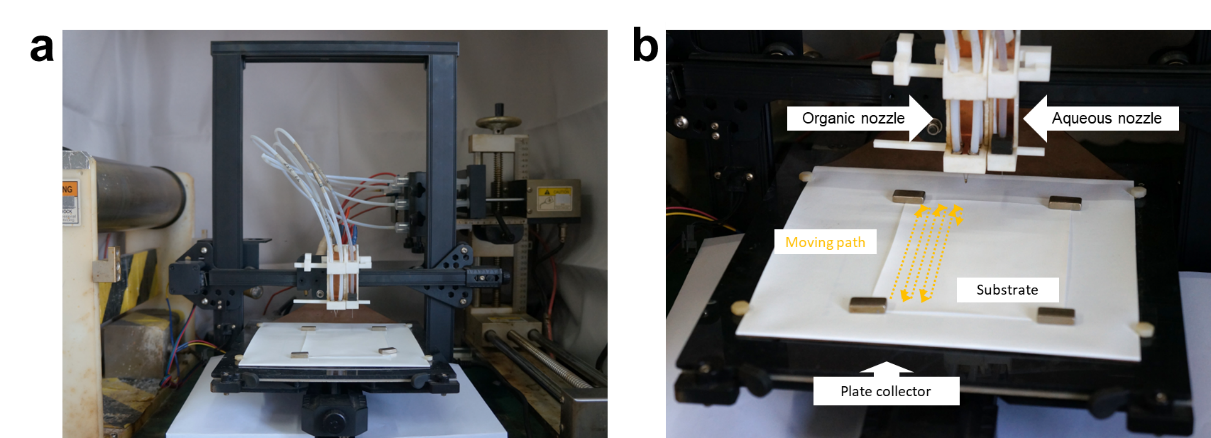


Figure S1.

Photo of (a) programmable electrospray 3D printer (PE3DP) and (b) moving of nozzle over collector.


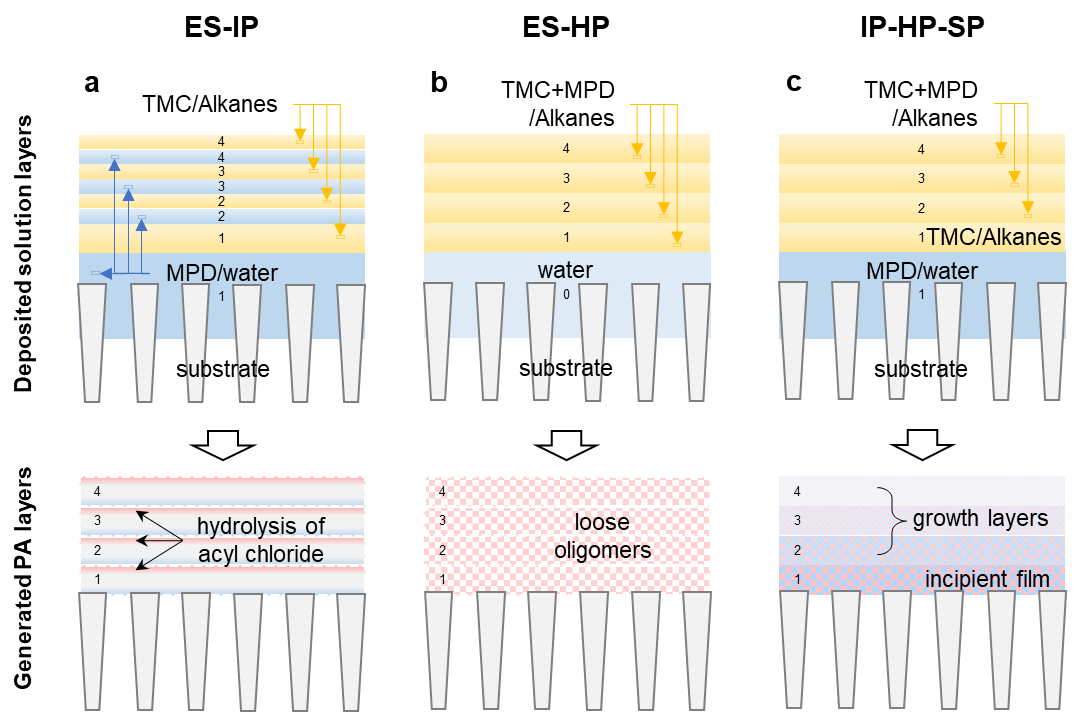


Figure S2.

Schematic for cross section of deposited solution layers and generated PA layers in (**a**) ES-IP, (**b**) ES-HP and (**c**) 3P method. The numbers in this figure represents number of scans by PE3DP. The color of PA layers represents the type of end groups (carboxy group in red and amine group in blue).


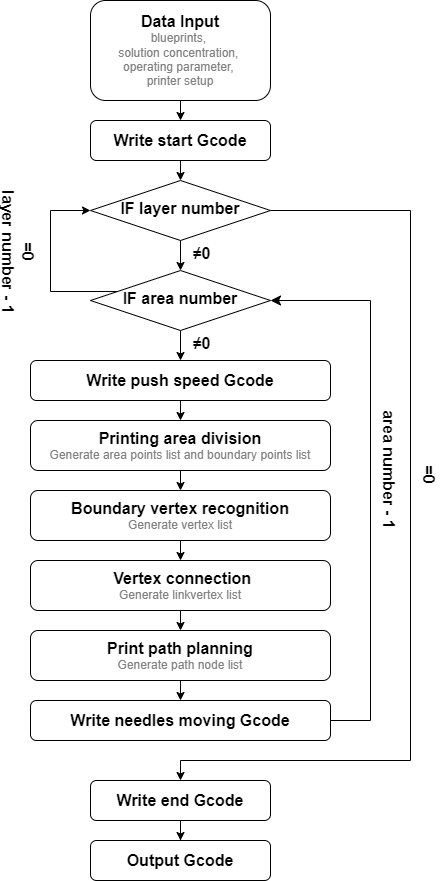


Figure S3.

Flow chart of the slicing program of PA layer printing generating Gcode files.


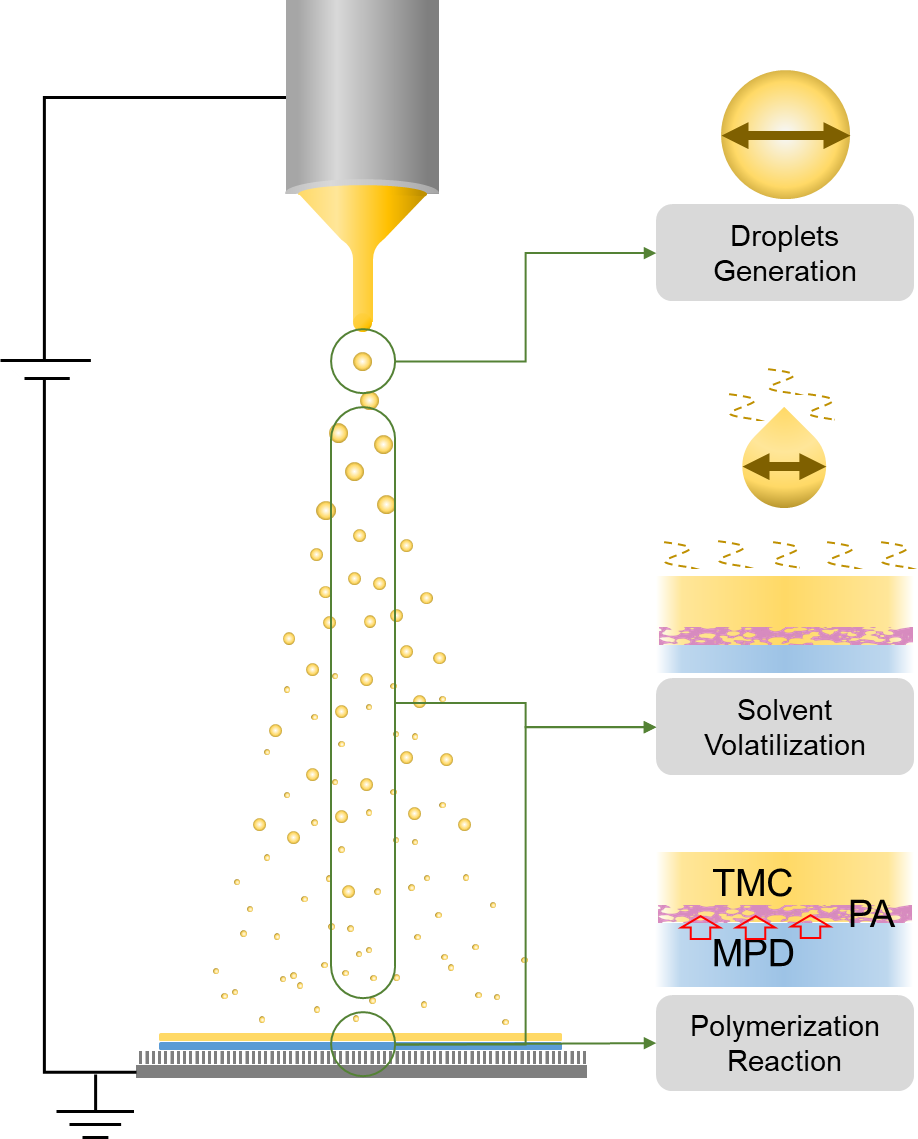


Figure S4.

Schematic for the numerical prediction model. It can be divided into three parts, droplet generation, solvent volatilization and polymerization reaction.


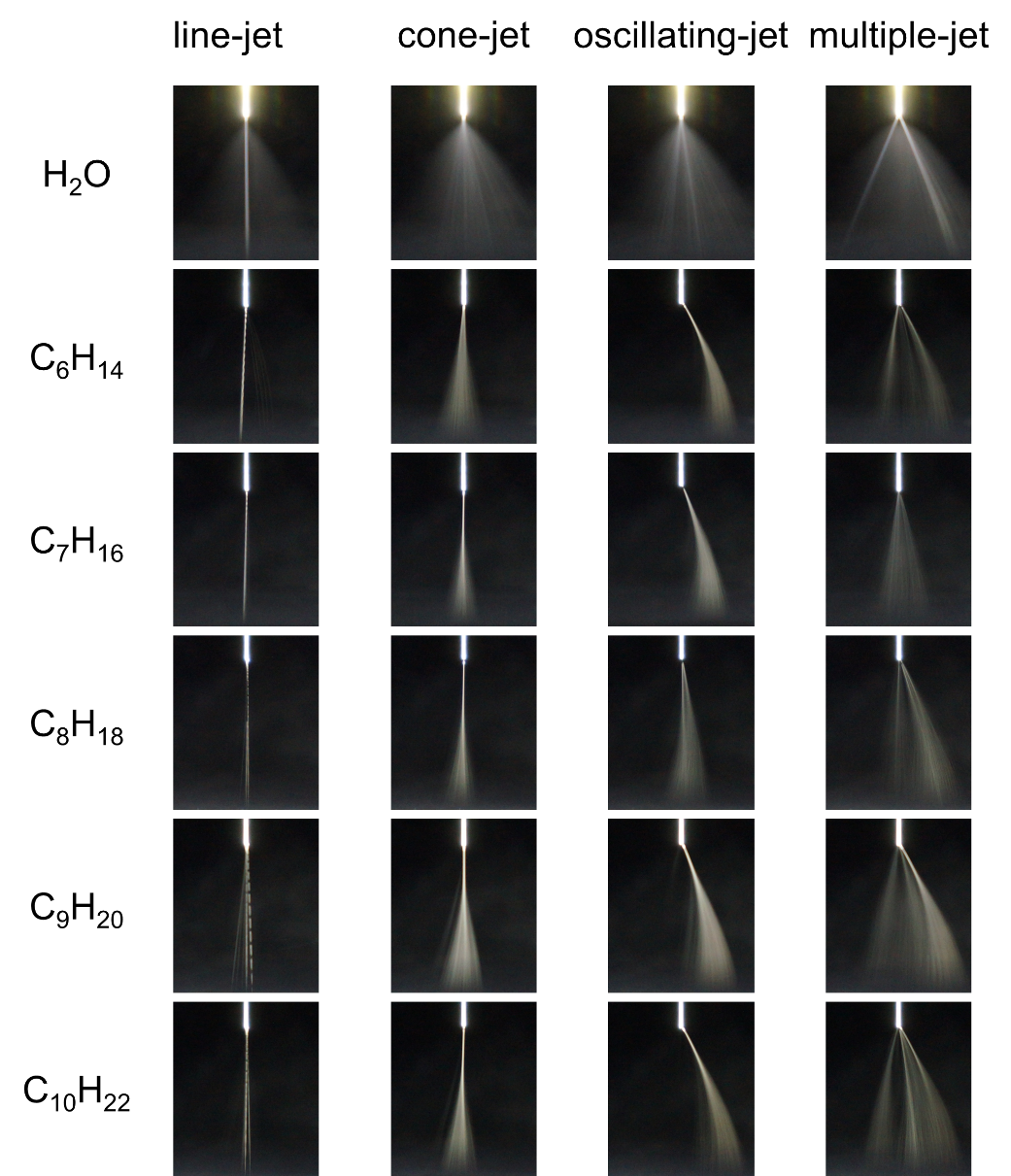


Figure S5.

Images for the ejection of aqueous and organic solutions from the needles in various electrospray modes.


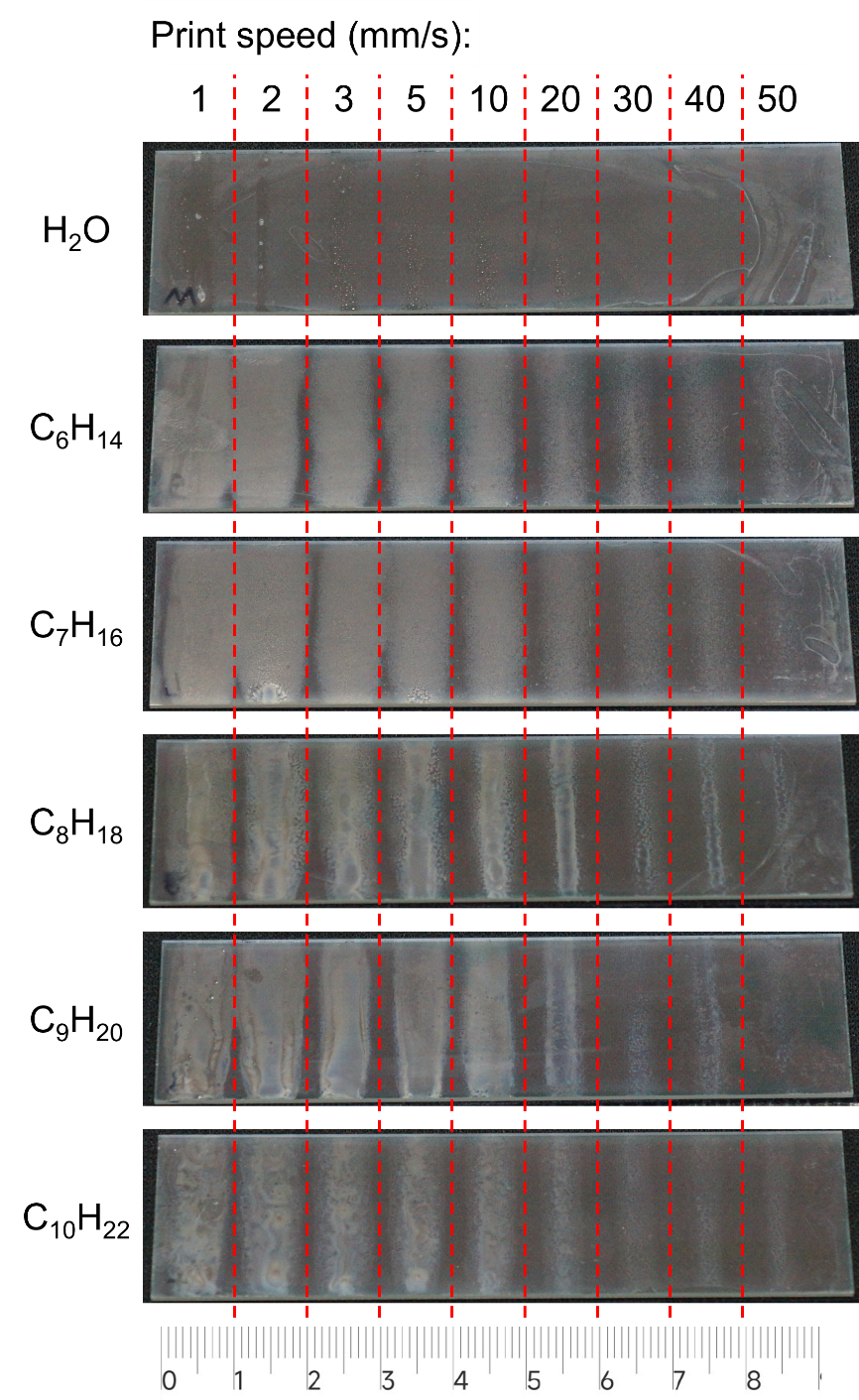


Figure S6.

Photos of aqueous and organic solution deposited on the surface of the collector.


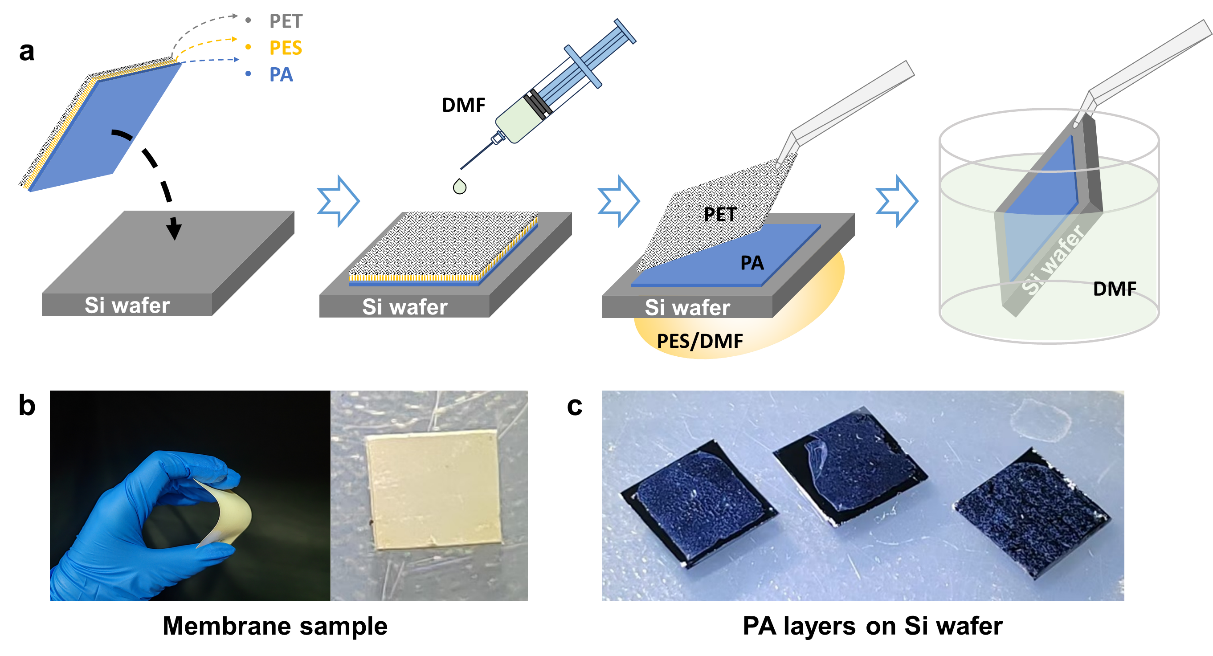


Figure S7.

**a**, Diagram of separation process for separating PA layer from the substrate. **b**, Samples of 3P membrane. **c**, The PA layers on Si wafer.


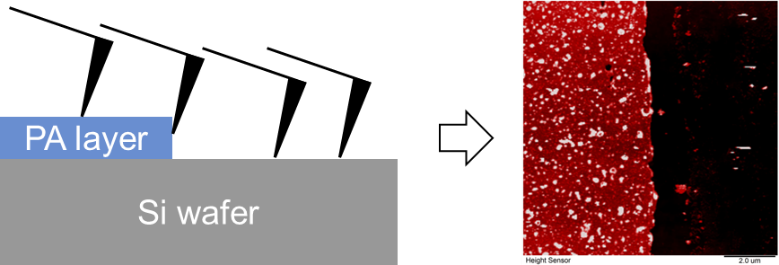


Figure S8.

Diagram of the measurement of PA layer thickness with AFM


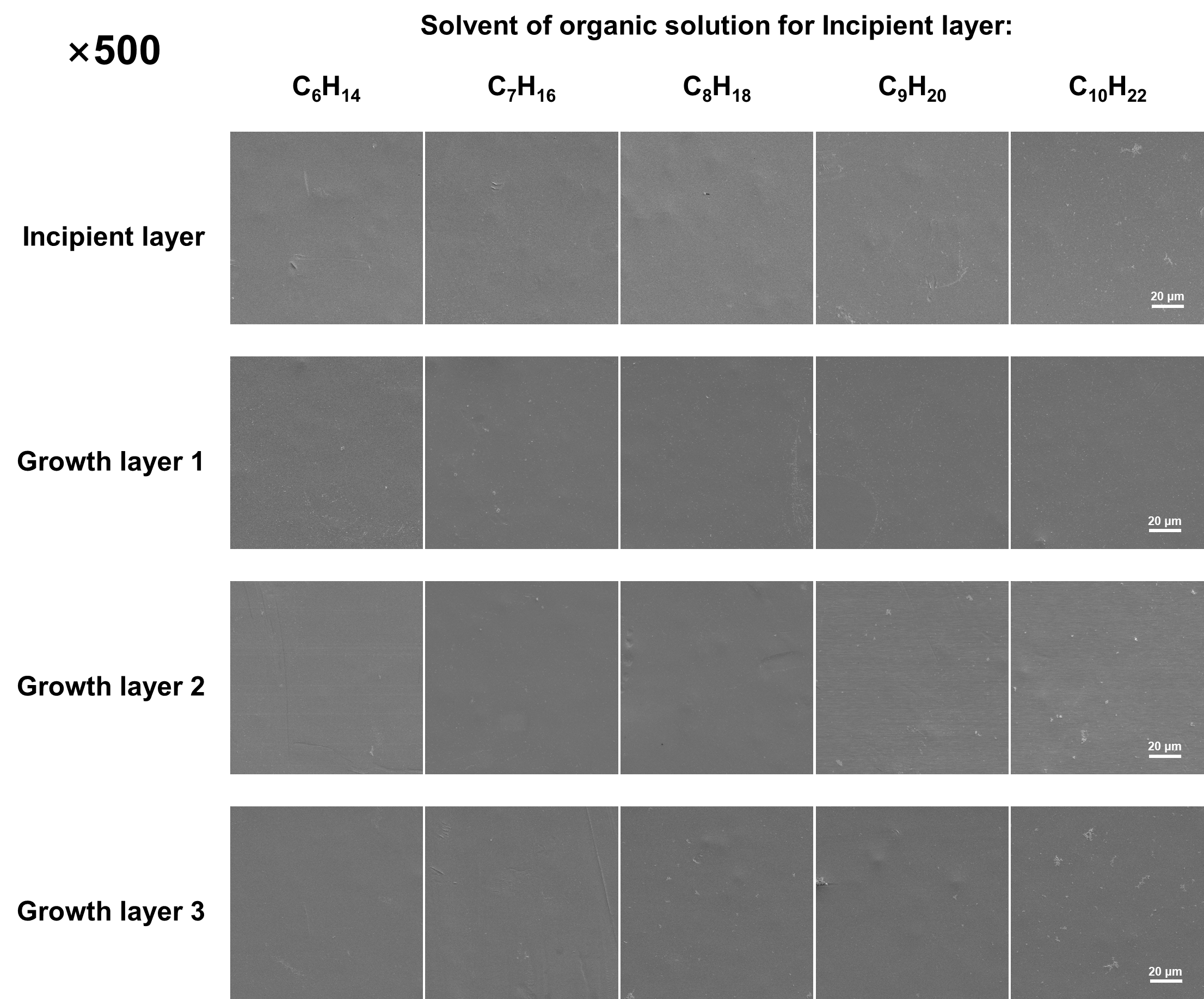


Figure S9.

SEM images (×500) for the surface of the incipient film and growth layers in various common 3P membranes prepared with different TMC organic solutions.


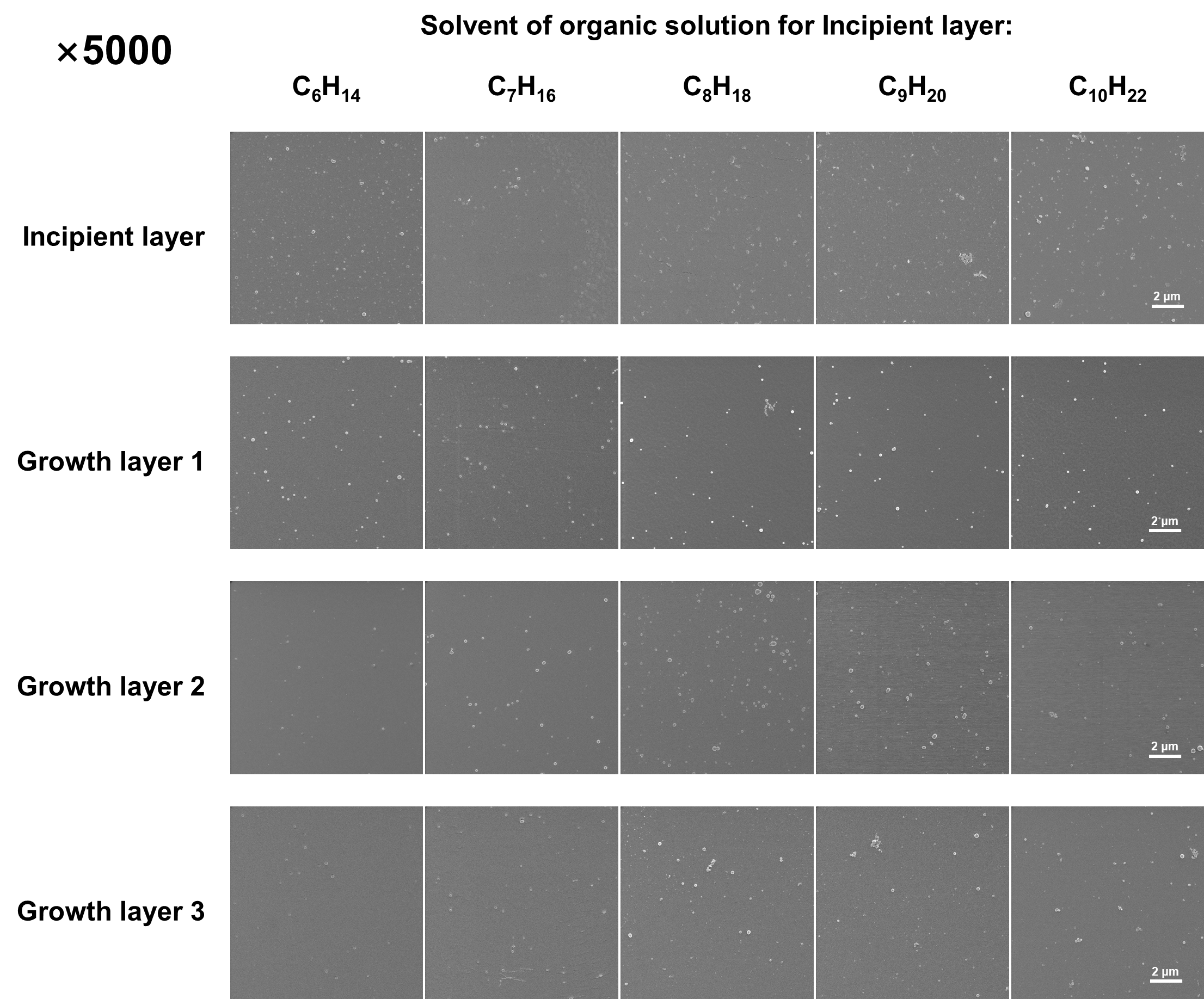


Figure S10.

SEM images (×5000) for the surface of the incipient film and growth layers in various common 3P membranes prepared with different TMC organic solutions.


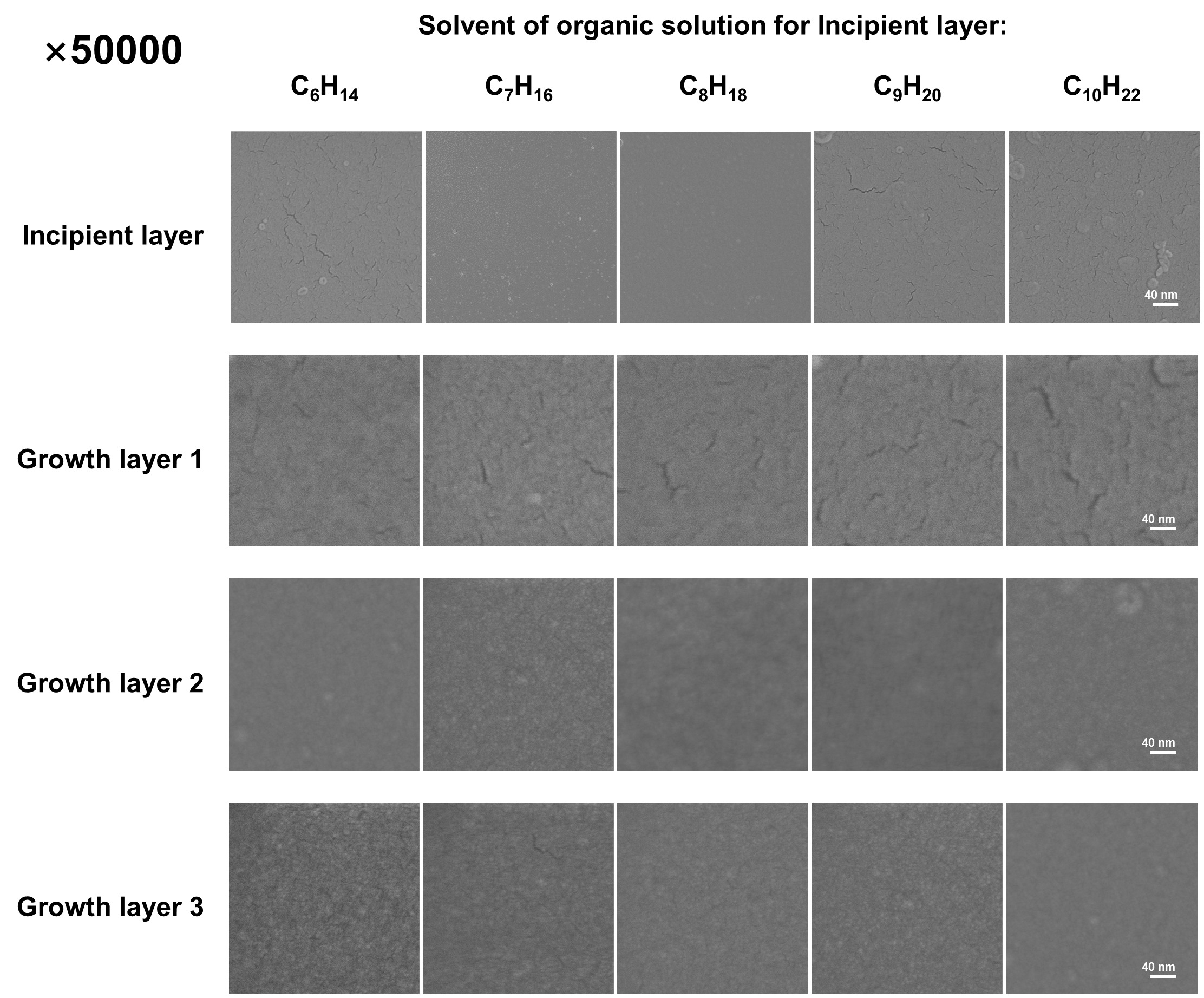


Figure S11.

SEM images (×50000) for the surface of the incipient film and growth layers in various common 3P membranes prepared with different TMC organic solutions.


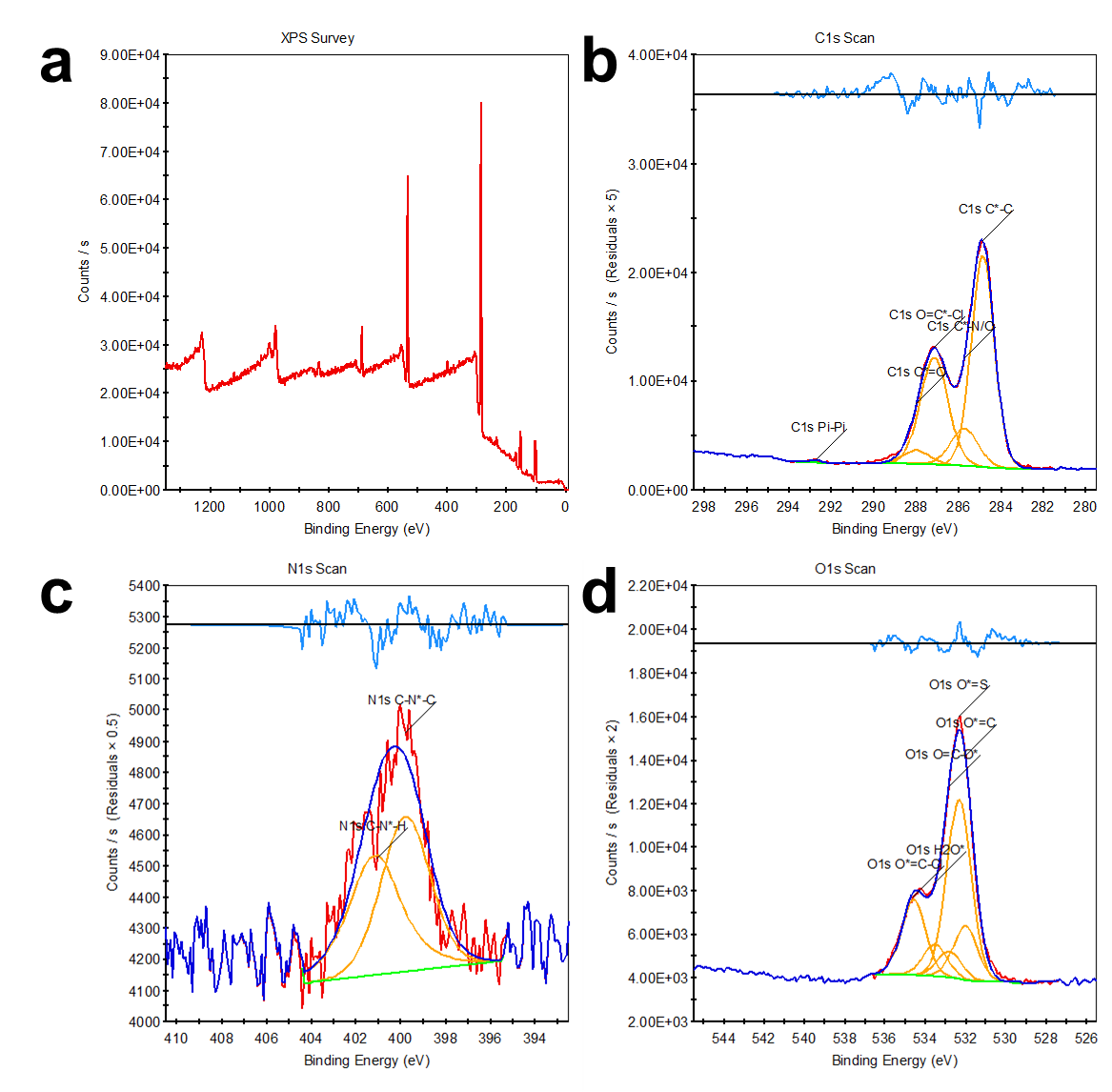


Figure S12.

XPS analysis results of the oligomers: (**a**) Survey XPS spectra; (**b**) C1s narrow spectra; (**c**) N1s narrow spectra; (**d**) O1s narrow spectra. According to the XPS results, the crosslinking degree of oligomers is less than 50%. In addition, peaks of amine and acyl chloride were observed in narrow spectra. The amount of acyl chloride groups is much larger than that of amine groups.


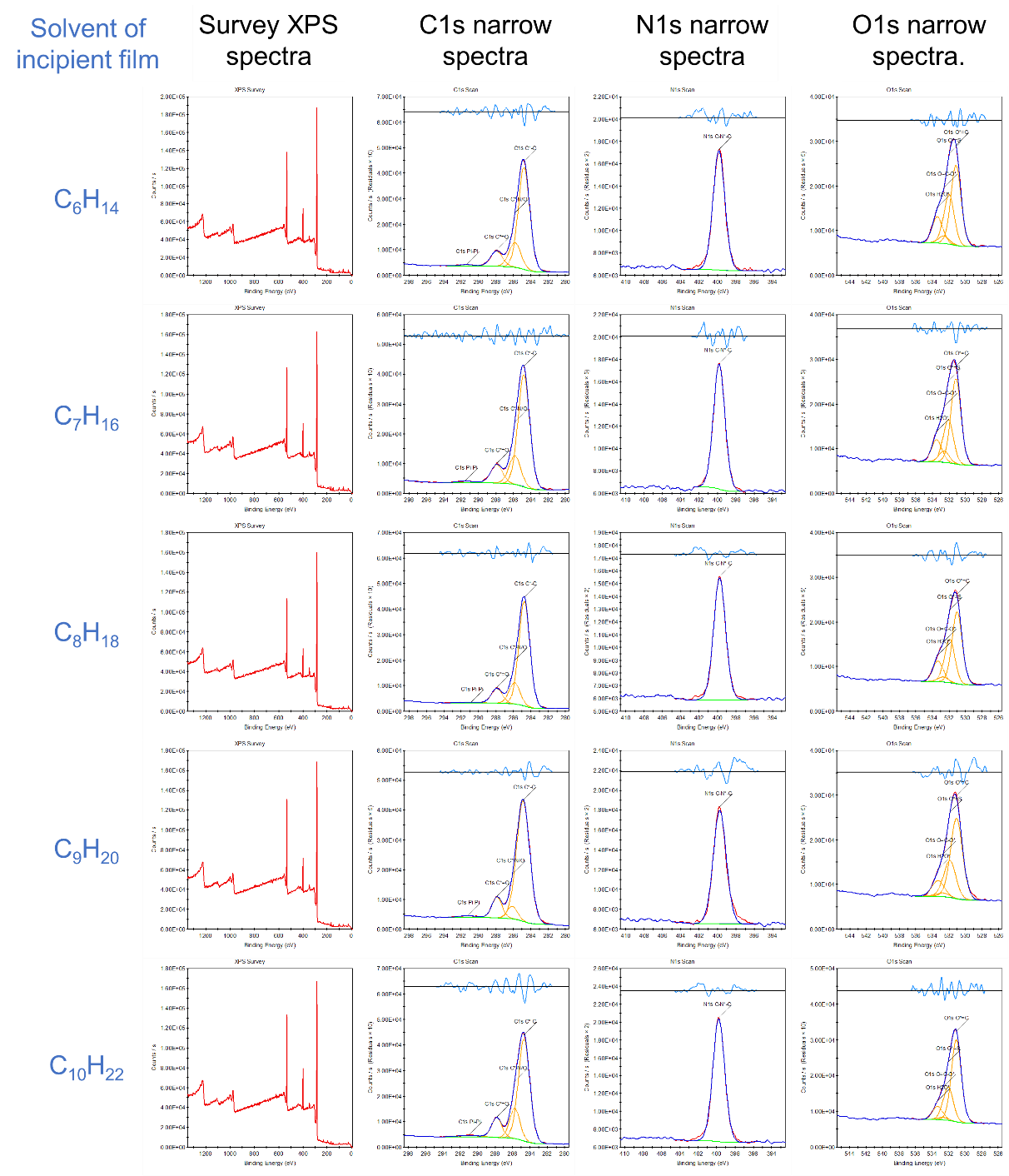


Figure S13.

XPS analysis results of the incipient film in the 3P membranes prepared with different TMC organic solutions.


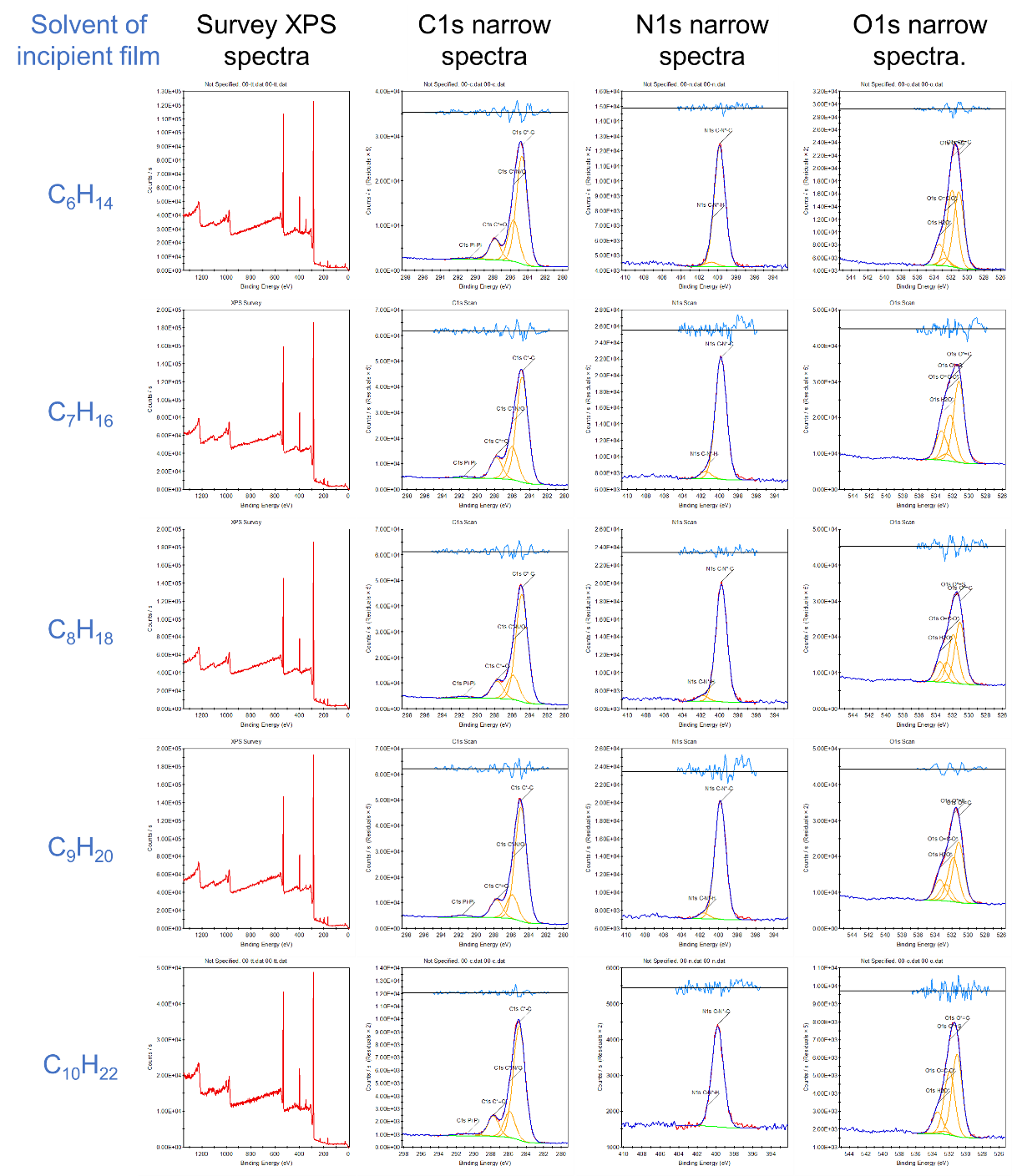


Figure S14.

XPS analysis results of the growth layers in the 3P membranes prepared with different TMC organic solutions.


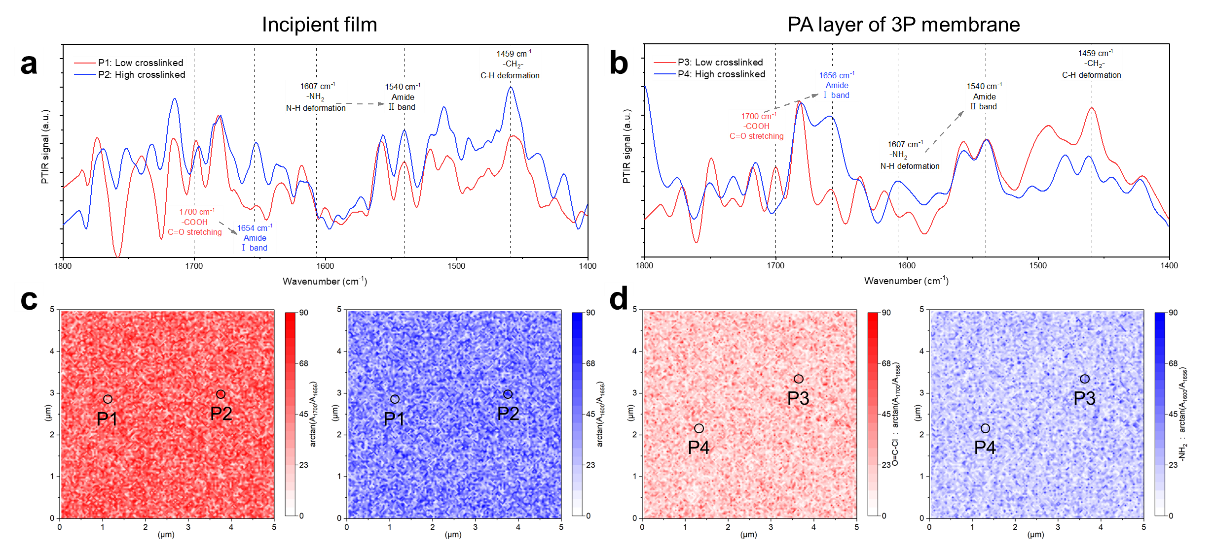


Figure S15.

AFM-IR analysis results of the incipient film and PA layer of 3P membrane. **a**, PTIR spectra of the incipient film with wavelength from 1800 cm^−1^ to 800 cm^−1^. **b**, PTIR spectra of the PA active layer with wavelength from 1800 1800 cm^−1^ to 800 cm^−1^. **c**, PTIR images of the incipient film. **d**, PTIR images of the PA active layer. In the PTIR images, red spots show the value of arctan(A_1700_/A_1656_) which represents the content of carboxyl group relative to amide bond; Blue spots show the value of arctan(A_1600_/A_1656_) which represents the content of amine group relative to amide bond.


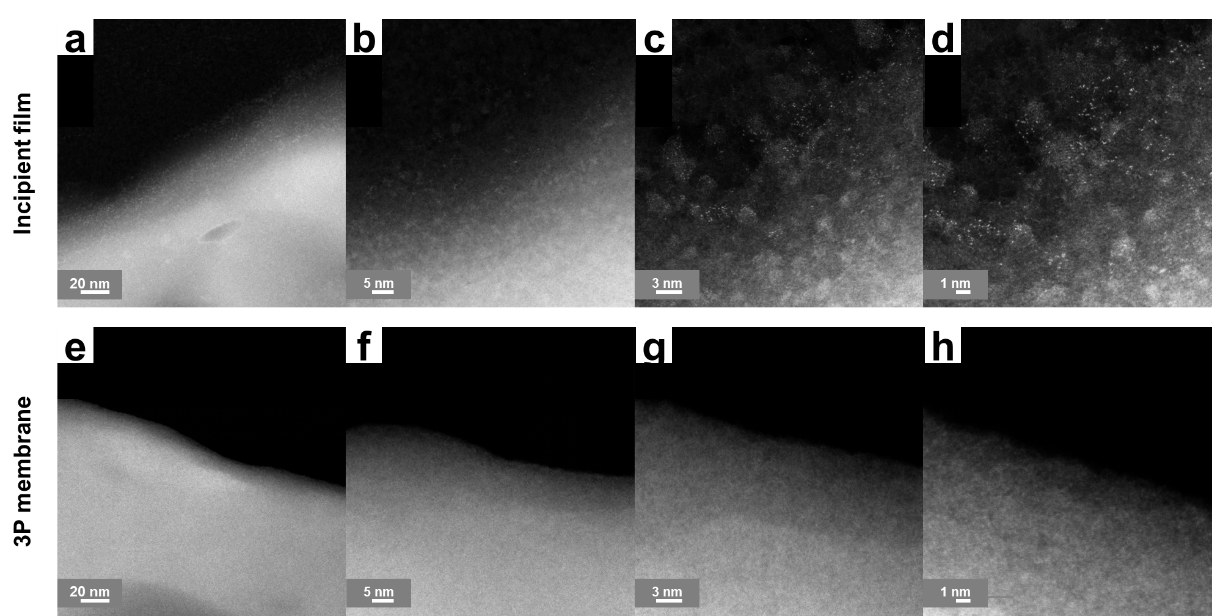


Figure S16.

Cross-sectional HAADF images of the incipient film and PA active layer of the 3P membrane. They were immersed in AgNO_3_ solution before analysis. After impregnation, silver binding between carboxyl groups and Ag^+^ was observed. The bound Ag^+^ would be brighter than the C, N, and O elements in the HAADF images. Therefore, the location of Ag^+^ in the incipient film and PA active of the 3P membrane can be determined in the cross-sectional HAADF images. As shown in Figure S14, many Ag^+^ are enriched in several paths in the incipient film. This indicates the existence of silver ion diffusion channels, which may be used as diffusion channels for MPD. However, similar paths are not found in the cross-sectional HAADF images of the PA layers. This indicates that the growth layers are tight, and the diffusion of Ag^+^ was significantly prohibited.


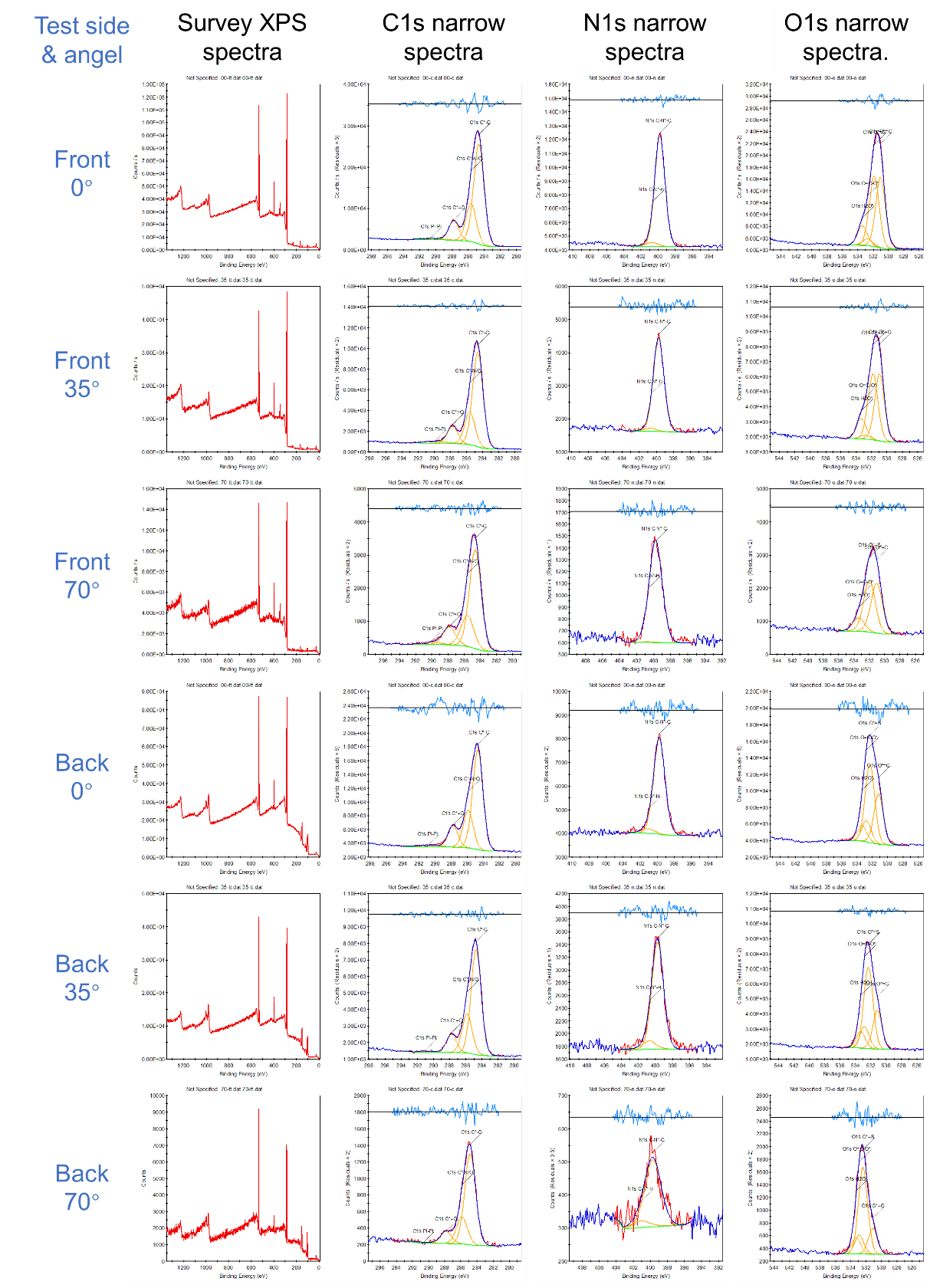


Figure S17.

ARXPS analysis results of the common 3P membrane in which the incipient film was printed with TMC/n-hexane solution (TMC/C_6_H_14_).


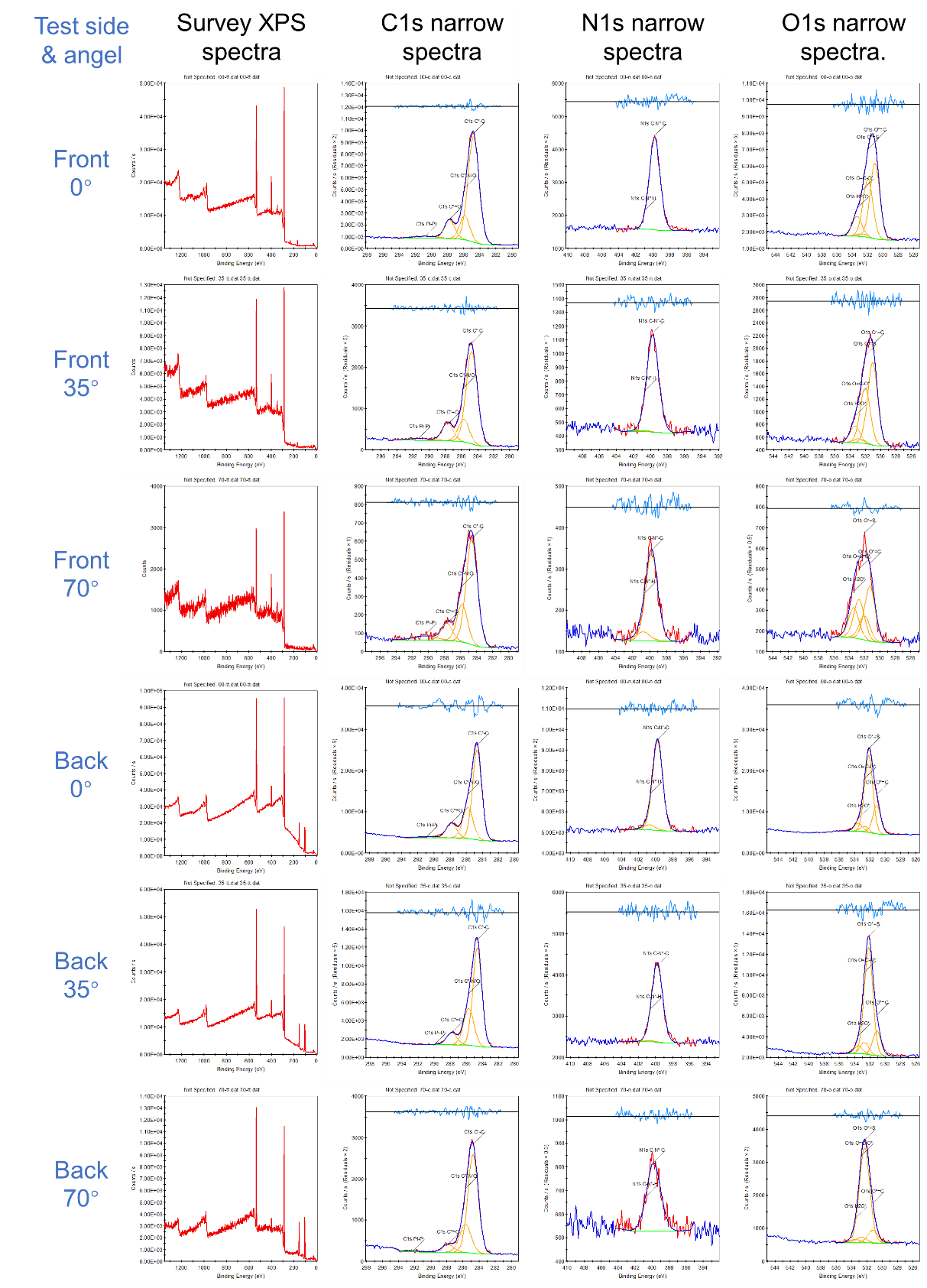


Figure S18.

ARXPS analysis results of the common 3P membrane in which the incipient film was printed with TMC/n-decane solution (TMC/C_10_H_22_).


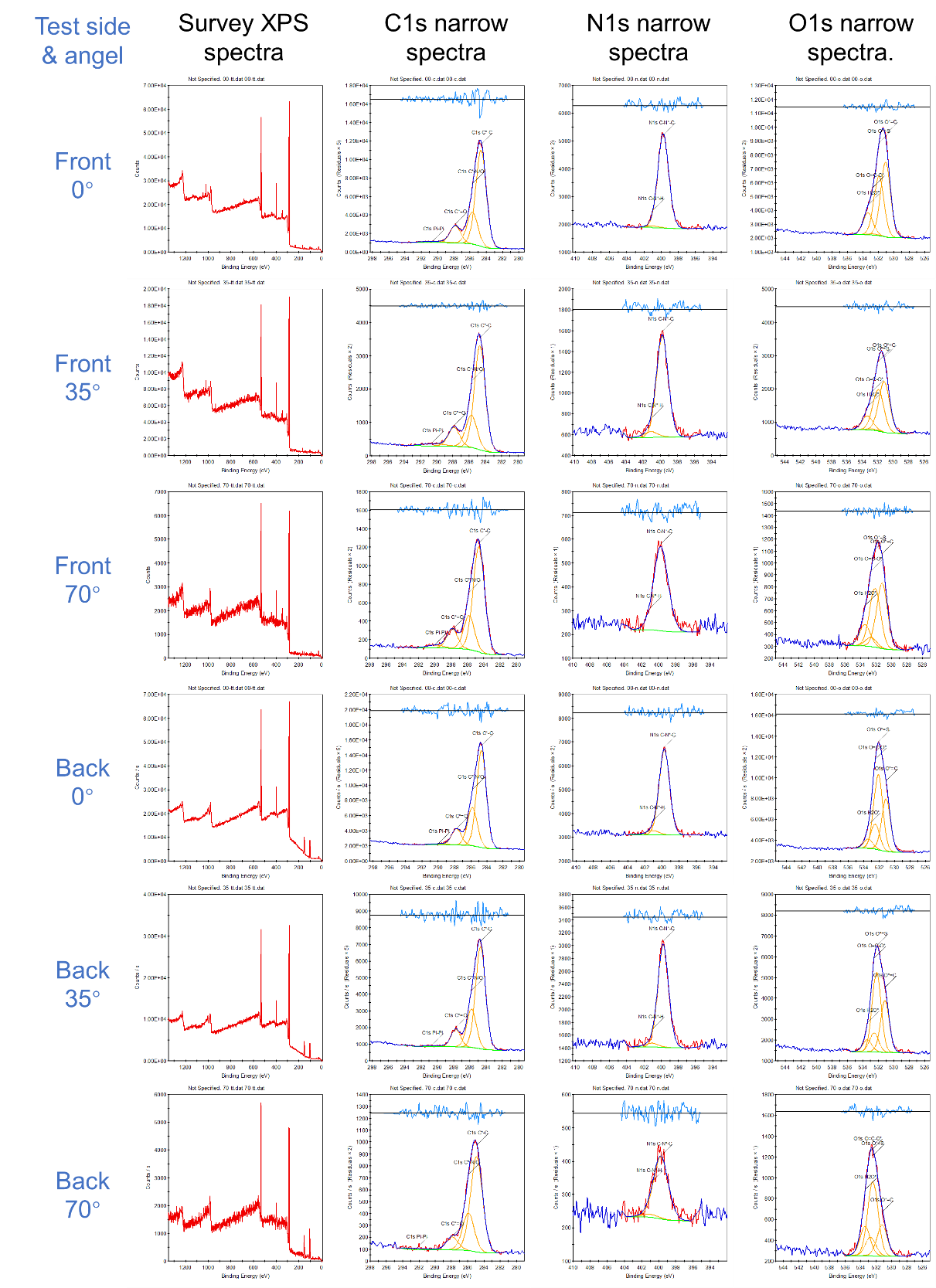


Figure S19.

ARXPS analysis results of the special 3P membrane.


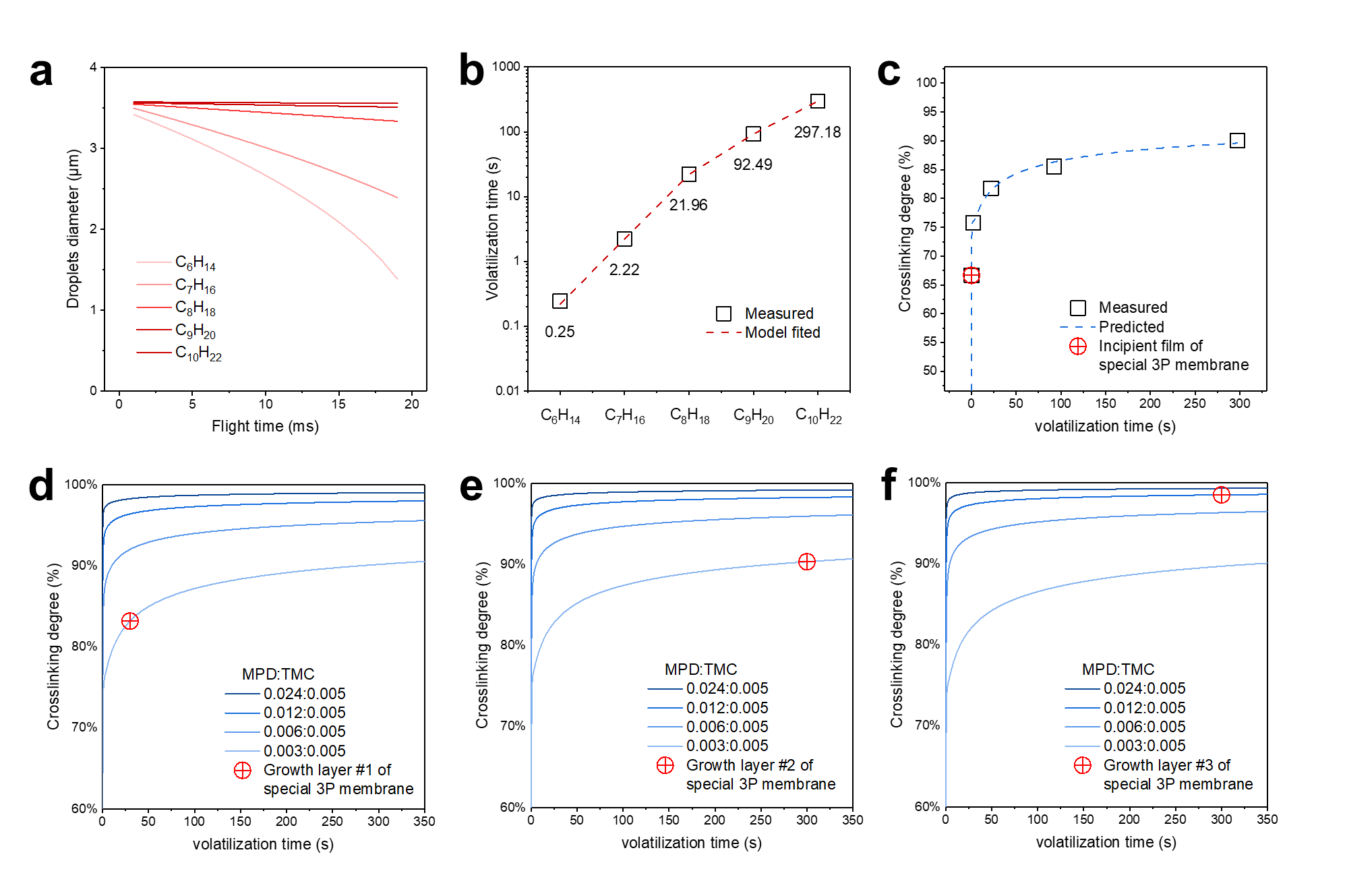


Figure S20.

Predicted and measured values of droplet diameter, volatilization time and amide bond content. **a**, Diameters of droplets for different organic solvents. **b**, Time required for organic solvent on the flat plate collector to completely evaporate. **c~f**, Non-linear relationship between crosslinking degree, monomer concentration and volatilization time that calculated with the model for incipient film, growth layer #1, growth layer #2 and growth layer #3. The preparation conditions of the special 3P membrane are marked with "⊕".


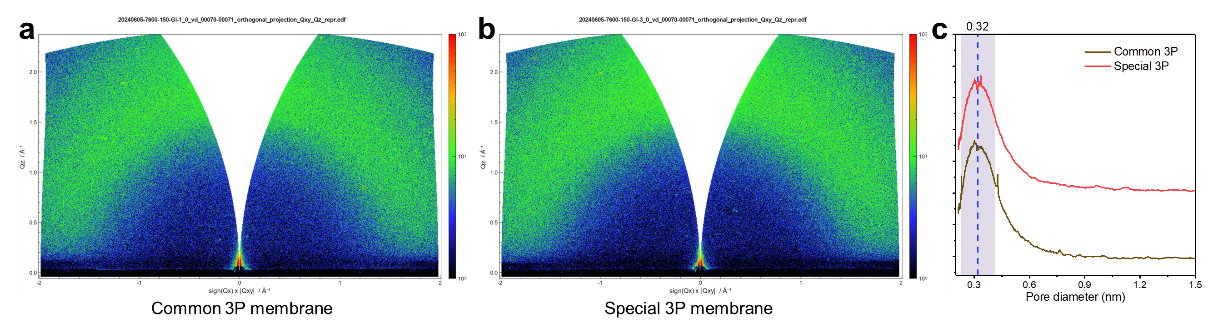


Figure S21.

GISAXS analysis results of PA layers from (**a**) common 3P membrane and (**b**) special 3P membrane, and (**c**) their pore size distributions.


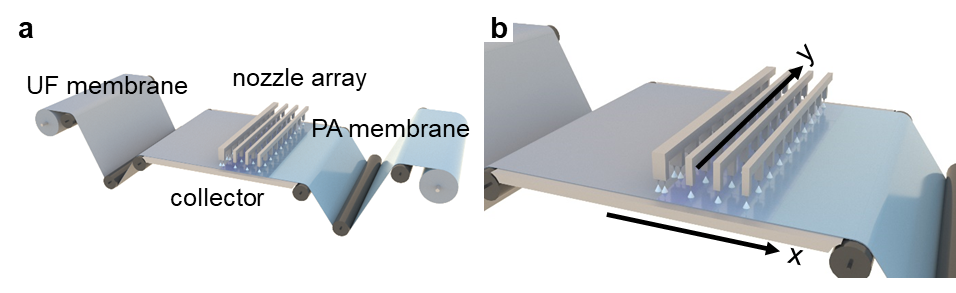


Figure S22.

Scale-up design for PE3DP. (a) system structure diagram; (b) moving mode.


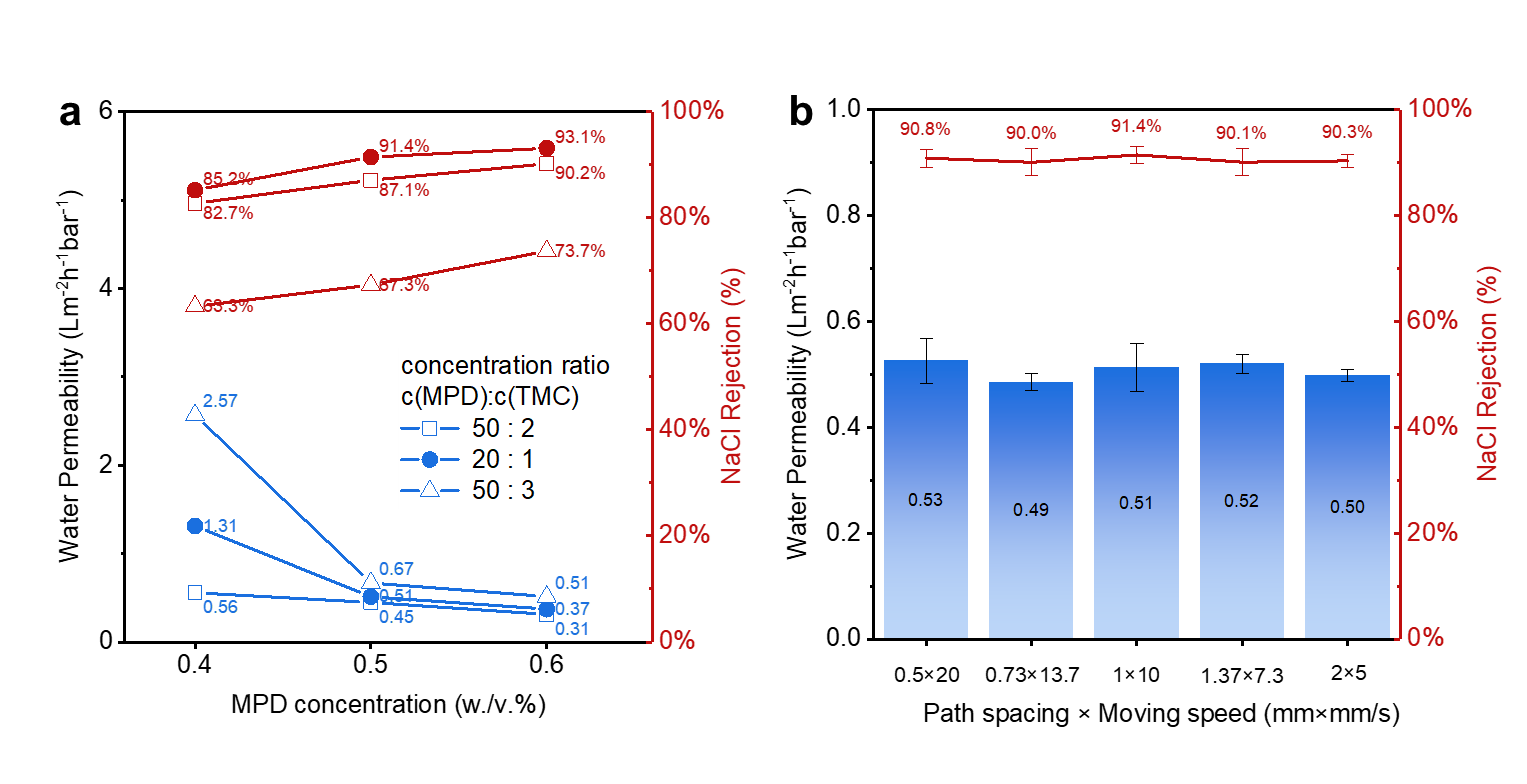


Figure S23.

Optimization of electrospray parameters for single scan. (a) concentration ratio; (b) path spacing and needle moving speed.


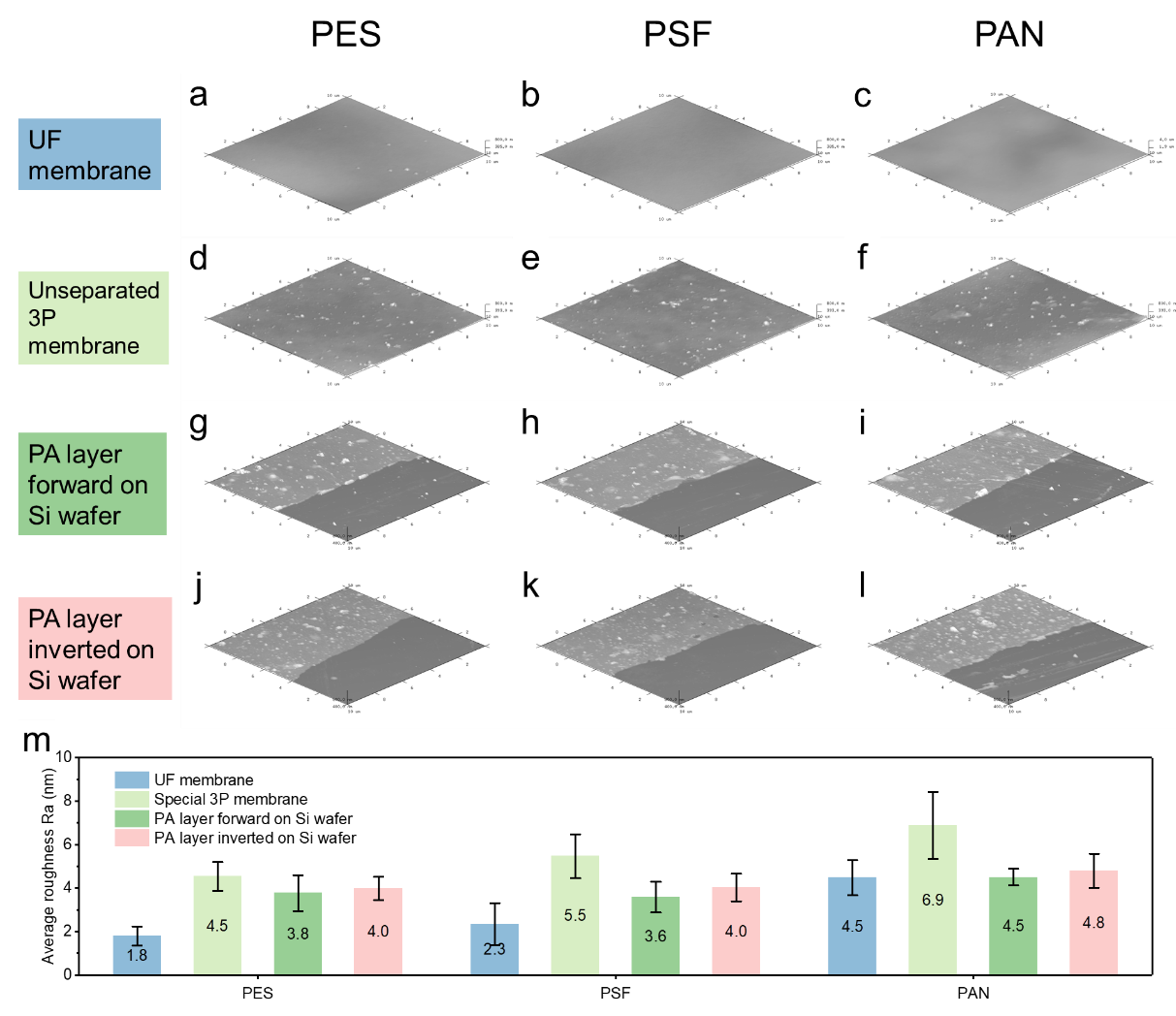


Figure S24.

AFM analysis of (a~c) UF membrane, (d~f) unseparated 3P membrane and (g~l) separated PA layer, and (m) the comparison of their average roughness. The roughness that was measured with the unseparated sample represents the surface roughness 3P membrane, and it was affected by the substrate. The fabrication of PA layer on the substrate resulted in an increase in roughness within 2~3 nm. The roughness that was measured with the separated PA layers represents the intrinsic roughness of PA layers. It is about 4 nm and shows the uniformity of the electrospray method in this study.

Table S1.

Optimal concentration of ionic liquid added into various organic solutions

| Alkanes | Surface tension  (10^−3^ N/m) | Ionic liquid concentration  (μL/mL) |
| --- | --- | --- |
| n-hexane | 18.02 | 0.75 |
| n-heptane | 20.18 | 0.84 |
| n-octane | 21.54 | 0.90 |
| n-nonane | 22.60 | 0.94 |
| n-decane | 23.44 | 0.98 |

Table S2.

Operating parameters of the PE3DP for printing the common and special 3P membranes

| **Method** | | **Needle**  **size** | **Distance between tip and collector** | **Voltage** | **Monomer solution** | **Solvent** | **Flowrate of solution** | **Path**  **spacing** | **Needle moving**  **Speed** | **Number of scans** |
| --- | --- | --- | --- | --- | --- | --- | --- | --- | --- | --- |
|  |  | gauge | mm | kV | / | / | mL/h | mm | mm/s |  |
| Common 3P  membrane | incipient  film | 30 | 20 | 4.5 | 0.2%MPD | water | 1.05 | 1 | 10 | 1 |
|  |  |  |  |  | 0.010%TMC | alkanes | 1.55 |  |  |  |
|  | growth layers | 30 | 20 | 4.5 | 0.003%MPD+  0.005%TMC* | 100% decane* | 1.55** | 1 | 10 | 3 |
| Special 3P  membrane | incipient  film | 30 | 20 | 4.5 | 0.2%MPD | water | 1.05 | 1 | 10 | 1 |
|  |  |  |  |  | 0.010%TMC | hexane | 1.55 |  |  |  |
|  | growth layer #1 | 30 | 20 | 4.5 | 0.003%MPD+  0.005%TMC* | 90% hexane+  10% decane* | 1.55** | 1 | 10 | 1 |
|  | growth layer #2 | 30 | 20 | 4.5 | 0.003%MPD+  0.005%TMC* | 100% decane* | 1.55** |  |  | 1 |
|  | growth layer #3 | 30 | 20 | 4.5 | 0.0012%MPD+  0.005%TMC* | 100% decane* | 1.55** |  |  | 1 |

* These values give concentration of monomers and the ratio of solvents in the mixed organic solutions.

** The total flow rate of the mixed organic solutions.

Table S3.

Calculated values of amide bond content and reaction time that used in nonlinear fitting.

| Solvent of TMC solution | Reaction time (s) | Amide bond content (mol/L) |
| --- | --- | --- |
| n-hexane | 0.245 | 8.44 |
| n-heptane | 2.22 | 8.73 |
| n-octane | 21.96 | 8.92 |
| n-nonane | 92.49 | 9.05 |
| n-decane | 297.18 | 9.19 |

Table S4.

Vaues of the model parameters (k, N_PA_^max^, D_MPD,0_, α and C_2_) obtained from nonlinear fitting

| Parameter | Physical meanings | Value | Unit |
| --- | --- | --- | --- |
| k | second-order reaction constant | 1.32×10^7^ | cm^3^/(mol·s) |
| N_PA_^max^ | maximum Amide bond content | 9.50 | mol/L |
| D_MPD,0_ | diffusion coefficient of MPD in the pure solvent | 1.71×10^−6^ | cm^2^/s |
| α | bending factor | 5 | - |
| C_2_ | constant | 4.10 | s |
| R^2^ | correlation coefficient | 0.999 | - |

Table S5.

Predicted and measured values of the crosslinking degree and thickness for the incipient film and growth layers for the common and special 3P membranes.

| Layers | Crosslinking degree (%) | | | |
| --- | --- | --- | --- | --- |
|  | Common 3P membrane | | Special 3P membrane | |
|  | predicted | Measured | predicted | measured |
| Incipient film | 67 | 69 | 67 | 71 |
| Growth layer 1 | 88 | 85 | 80 | 75 |
| Growth layer 2 | 87 | 84 | 90 | 88 |
| Growth layer 3 | 85 | 83 | 99 | 97 |
| Layers | Thickness (nm) | | | |
|  | Common 3P membrane | | Special 3P membrane | |
|  | predicted | Measured | predicted | measured |
| Incipient film | 6.8 | 6.7 | 6.8 | 6.7 |
| Growth layer 1 | 2.8 | 2.1 | 3.0 | 2.5 |
| Growth layer 2 | 2.8 | 2.3 | 2.8 | 2.8 |
| Growth layer 3 | 2.9 | 2.1 | 2.6 | 2.9 |

Table S6.

Thickness and intrinsic roughness of the incipient film and PA layer of various membranes.

| Sample | Solvents | Thickness  (nm) | Ra  (nm) | Rq  (nm) | SAD  (%) |
| --- | --- | --- | --- | --- | --- |
| Incipient film | n-hexane | 6.7 | 1.6 | 2.8 | 0.28 |
|  | n-heptane | 7.3 | 1.5 | 3.2 | 0.12 |
|  | n-octane | 7.6 | 1.8 | 3.6 | 0.32 |
|  | n-nonane | 7.8 | 1.7 | 3.1 | 0.22 |
|  | n-decane | 8.1 | 1.4 | 2.5 | 0.13 |
| PA active layer of the common  3P  membranes | n-hexane | 13.1 | 3.7 | 5.5 | 0.97 |
|  | n-heptane | 13.5 | 4.4 | 6.0 | 0.90 |
|  | n-octane | 14.3 | 3.7 | 5.4 | 0.83 |
|  | n-nonane | 14.7 | 3.6 | 5.9 | 1.00 |
|  | n-decane | 14.8 | 4.0 | 6.2 | 1.06 |
| Sample | No. of scans | Thickness  (nm) | Ra  (nm) | Rq  (nm) | SAD  (%) |
| Common  3P  membrane | 1^a^ | 6.7 | 1.6 | 2.8 | 0.28 |
|  | 2^b^ | 8.7 | 2.8 | 5.6 | 0.77 |
|  | 3^c^ | 11.0 | 2.2 | 4.6 | 0.33 |
|  | 4^d^ | 13.1 | 3.7 | 5.5 | 0.97 |
| Special  3P  membrane | 1^a^ | 6.7 | 1.6 | 2.8 | 0.28 |
|  | 2^b^ | 9.1 | 2.7 | 5.5 | 0.44 |
|  | 3^c^ | 11.9 | 4.7 | 6.4 | 1.35 |
|  | 4^d^ | 14.7 | 4.6 | 6.2 | 0.79 |

^a^ Thickness and intrinsic roughness for the incipient film; ^b^ Thickness and intrinsic roughness for the PA layer consisting of the incipient film and the first growth layer; ^c^ Thickness and intrinsic roughness for the PA layer consisting of the incipient film and the first and second growth layers; ^d^ Thickness and intrinsic roughness for the PA layer.

Table S7.

Properties of the substrate.

| UF membrane | water permeability  (LMH bar^-1^) | molecular weight cut-off  (MWCO) | conductivity  - dry membrane  (μS·cm^-1^) | conductivity  - wetted membrane*  (μS·cm^-1^) |
| --- | --- | --- | --- | --- |
| Polyether sulfone (PES) | 422 | 20000 | 2.9×10^-10^ | 7.8 |
| Polysulfone (PSF) | 516 | 20000 | 3.1×10^-11^ | 3.4 |
| Polyacrylonitrile (PAN) | 668 | 100000 | 3.2×10^-11^ | 13.9 |

* The UF membranes were wetted by the aqueous solution (20 μS/cm).

Video S1.

Taylor cone at the tip of separate needle.

Video S2.

Taylor cone at the tip of multi-in-one device.

Video S3.

Printing process of programmable electrospray 3D printer.

Video S4.

Optimization of voltage to realized stable Taylor cone-jet.

Code S1.

Slicing program for programmable electrospray 3D printer

References

[1] Y. Zhao, J. Zhu, W. He, Y. Liu, X. Sang, R. Liu, *Nat. Commun.* **2023**, 14, 2381.

[2] T. Chen, P. Xu, M. Qiu, X. Chen, Z. Zhong, Y. Fan, *J. Membr. Sci.* **2024**, 706, 122941.

[3] N. Salitra, J. Gurauskis, H. Groger, *Angew. Chem. Int. Ed.* **2024**, 63, e202316760.

[4] W. Jung, Y. H. Jung, P. V. Pikhitsa, J. Feng, Y. Yang, M. Kim, H. Y. Tsai, T. Tanaka, J. Shin, K. Y. Kim, H. Choi, J. Rho, M. Choi, *Nature* **2021**, 592, 54.

[5] A. Jarlöv, Z. Zhu, W. Ji, S. Gao, Z. Hu, P. Vivegananthan, Y. Tian, D. R. Kripalani, H. Fan, H. L. Seet, C. Han, L. Tan, F. Liu, M. L. S. Nai, K. Zhou, *Mater. Sci. Eng. R Rep.* **2024**, 161, 100834.

[6] J. Feng, T. Gao, F. Morlet-Savary, M. Schmitt, C. Dietlin, J. Zhang, X. Peng, P. Xiao, F. Dumur, J. Lalevee, *Angew. Chem. Int. Ed.* **2025**, e202425198.

[7] M. I. Segal, A. J. Bahnick, N. G. Judge, M. L. Becker, *Angew. Chem. Int. Ed.* **2025**, 64, e202414016.

[8] S. J. Lee, W. Jeong, A. Atala, *Adv. Mater.* **2024**, 36, e2408032.

[9] J. M. Kronenfeld, L. Rother, M. A. Saccone, M. T. Dulay, J. M. DeSimone, *Nature* **2024**, 627, 306.

[10] Z.-X. Low, Y. T. Chua, B. M. Ray, D. Mattia, I. S. Metcalfe, D. A. Patterson, *J. Membr. Sci.* **2017**, 523, 596.

[11] W. Xu, S. Jambhulkar, D. Ravichandran, Y. Zhu, M. Kakarla, Q. Nian, B. Azeredo, X. Chen, K. Jin, B. Vernon, D. G. Lott, J. L. Cornella, O. Shefi, G. Miquelard-Garnier, Y. Yang, K. Song, *Small* **2021**, 17, e2100817.

[12] M. A. Shannon, P. W. Bohn, M. Elimelech, J. G. Georgiadis, B. J. Marinas, A. M. Mayes, *Nature* **2008**, 452, 301.

[13] M. Elimelech, W. A. Phillip, *Science* **2011**, 333, 712.

[14] A. Razmjou, M. Asadnia, E. Hosseini, A. Habibnejad Korayem, V. Chen, *Nat. Commun.* **2019**, 10, 5793.

[15] Y. Liang, Y. Zhu, C. Liu, K. R. Lee, W. S. Hung, Z. Wang, Y. Li, M. Elimelech, J. Jin, S. Lin, *Nat. Commun.* **2020**, 11, 2015.

[16] D. Lu, T. Ma, S. Lin, Z. Zhou, G. Li, Q. An, Z. Yao, Q. Sun, Z. Sun, L. Zhang, *J. Membr. Sci.* **2021**, 635, 119504.

[17] D. L. Gin, R. D. Noble, *Science* **2011**, 332, 674.

[18] S. Karan, Z. Jiang, A. G. Livingston, *Science* **2015**, 348, 1347.

[19] P. Sarkar, S. Modak, S. Ray, V. Adupa, K. A. Reddy, S. Karan, *J. Mater. Chem. A Mater.* **2021**, 9, 20714.

[20] Z. Tan, S. Chen, X. Peng, L. Zhang, C. Gao, *Science* **2018**, 360, 518.

[21] X. Song, B. Gan, S. Qi, H. Guo, C. Y. Tang, Y. Zhou, C. Gao, *Environ. Sci. Technol.* **2020**, 54, 3559.

[22] S. Wang, S. Bing, Y. Li, Y. Zhou, L. Zhang, C. Gao, *J. Membr. Sci.* **2021**, 628, 119230.

[23] T. E. Culp, B. Khara, K. P. Brickey, M. Geitner, T. J. Zimudzi, J. D. Wilbur, S. D. Jons, A. Roy, M. Paul, B. Ganapathysubramanian, A. L. Zydney, M. Kumar, E. D. Gomez, *Science* **2021**, 371, 72.

[24] J. He, J. Yang, J. R. McCutcheon, Y. Li, *J. Membr. Sci.* **2022**, 658, 120731.

[25] P. Sarkar, C. Wu, Z. Yang, C. Y. Tang, *Chem. Soc. Rev.* **2024**, 53, 4374.

[26] P. W. Morgan, S. L. Kwolek, *J. Polym. Sci.* **1959**, 40, 299.

[27] V. Freger, S. Srebnik, *J. Appl. Polym. Sci.* **2003**, 88, 1162.

[28] J.-H. Xin, B.-B. Guo, C. Liu, C.-Y. Zhu, H. Zhang, B. Z. Tang, Z.-K. Xu, *Macromolecules* **2023**, 56, 5415.

[29] V. Freger, *Langmuir* **2005**, 21, 1884.

[30] S. Zhou, L. Long, Z. Yang, S. L. So, B. Gan, H. Guo, S. P. Feng, C. Y. Tang, *Environ. Sci. Technol.* **2022**, 56, 10279.

[31] J. Xu, H. Yan, Y. Zhang, G. Pan, Y. Liu, *J. Membr. Sci.* **2017**, 541, 174.

[32] H. B. Park, J. Kamcev, L. M. Robeson, M. Elimelech, B. D. Freeman, *Science* **2017**, 356, eaab0530.

[33] M. R. Chowdhury, J. Steffes, B. D. Huey, J. R. McCutcheon, *Science* **2018**, 361, 682.

[34] S. Huang, J. Mansouri, P. Le-Clech, G. Leslie, C. Y. Tang, A. G. Fane, *J. Membr. Sci.* **2022**, 646, 120248.

[35] Z. Ma, L.-F. Ren, D. Ying, J. Jia, J. Shao, *J. Membr. Sci.* **2021**, 632, 119369.

[36] C. Y. Tang, Y.-N. Kwon, J. O. Leckie, *Desalination* **2009**, 242, 149.

[37] A. Gañán-Calvo, J. Dávila, A. Barrero, *J. Aerosol. Sci.* **1997**, 28, 249.

[38] R. W. Paynter, *Surf. Interface. Anal.* **1999**, 27, 103.

[39] Y. Gao, Y. Zhao, X.-m. Wang, C. Tang, X. Huang, *Environ. Sci. Technol.* **2022**, 56, 14038.

[40] B. Gan, S. Qi, X. Song, Z. Yang, C. Y. Tang, X. Cao, Y. Zhou, C. Gao, *J. Membr. Sci.* **2020**, 612, 118402.

[41] S. Huang, J. Mansouri, J. A. McDonald, S. J. Khan, G. Leslie, C. Y. Tang, A. G. Fane, *J. Membr. Sci.* **2023**, 673, 121461.

[42] J. E. Gu, S. Lee, C. M. Stafford, J. S. Lee, W. Choi, B. Y. Kim, K. Y. Baek, E. P. Chan, J. Y. Chung, J. Bang, J. H. Lee, *Adv. Mater.* **2013**, 25, 4778.

[43] W. Choi, S. Jeon, S. J. Kwon, H. Park, Y.-I. Park, S.-E. Nam, P. S. Lee, J. S. Lee, J. Choi, S. Hong, E. P. Chan, J.-H. Lee, *J. Membr. Sci.* **2017**, 527, 121.

[44] S.-J. Park, W. Choi, S.-E. Nam, S. Hong, J. S. Lee, J.-H. Lee, *J. Membr. Sci.* **2017**, 526, 52.

[45] Z. Jiang, S. Karan, A. G. Livingston, *Adv. Mater.* **2018**, 30, e1705973.

[46] Y. Wen, X. Zhang, X. Li, Z. Wang, C. Y. Tang, *ACS Applied Nano Materials* **2020**, 3, 9238.

[47] H.-Q. Huo, Y.-F. Mi, X. Yang, H.-H. Lu, Y.-L. Ji, Y. Zhou, C.-J. Gao, *J. Membr. Sci.* **2023**, 669.

[48] Y. Qin, P. Qi, S. Hao, W. Shi, J. Xiao, J. Wang, Y. Hu, *Nature Water* **2025**, 3, 110.

[49] S. Y. Kwak, M. O. Yeom, Ⅱ. J. Roh, D. Y. Kim, J. J. Kim, *Journal of Membrane Science Letters* **1997**, 132, 183.

[50] Y. Yao, P. Zhang, C. Jiang, R. M. DuChanois, X. Zhang, M. Elimelech, *Nature Sustainability* **2020**, 4, 138.

[51] Y. Liu, W. Fang, Z. Yue, Y. Wang, Y. Zhu, J. Jin, L. Jiang, *Nature Water* **2025**, 3, 430.

[52] H. Wang, J. Zhao, Y. Li, Y. Cao, Z. Zhu, M. Wang, R. Zhang, F. Pan, Z. Jiang, *Nanomicro Lett* **2022**, 14, 216.

[53] Z. Li, Y. Zheng, T. Gu, X. Meng, H. Wang, K. Xu, L. Cheng, R. Kasher, R. Zhang, Z. Jiang, *J. Membr. Sci.* **2023**, 675.

[54] I. Koyuncu, J. Brant, A. Lüttge, M. R. Wiesner, *J. Membr. Sci.* **2006**, 278, 410.

[55] J. Du, H. Yuan, H. Kou, Z. Yao, P. Gao, P. Wu, C. Zhang, *Appl. Surf. Sci.* **2024**, 663, 160194.

[56] S. Tanuma, C. J. Powell, D. R. Penn, *Surf. Interface. Anal.* **2004**, 20, 77.

[57] M. Tuteja, M. Kang, C. Leal, A. Centrone, *Analyst* **2018**, 143, 3808.

[58] L. Quaroni, *Analyst* **2020**, 145, 5940.

[59] C. Lu, C. Hu, Z. Chen, P. Wang, F. Feng, G. He, F. Wang, Y. Zhang, J. Z. Liu, X. Zhang, J. Qu, *Sci. Adv.* **2023**, 9, eadf8412.

[60] Q. Fu, N. Verma, H. Ma, F. J. Medellin-Rodriguez, R. Li, M. Fukuto, C. M. Stafford, B. S. Hsiao, B. M. Ocko, *ACS Macro Lett.* **2019**, 8, 352.

[61] P. S. Singh, P. Ray, Z. Xie, M. Hoang, *J. Membr. Sci.* **2012**, 421-422, 51.

[62] X. Tian, Z. Jing, J. Wang, Y. Wei, L. Tian, H. Wang, N. Chang, *J. Membr. Sci.* **2024**, 693, 122330.

[63] J. T. Arena, B. McCloskey, B. D. Freeman, J. R. McCutcheon, *J. Membr. Sci.* **2011**, 375, 55.

[64] N.-N. Bui, J. R. McCutcheon, *Environ. Sci. Technol.* **2013**, 47, 1761.

[65] G. T. Gray, J. R. McCutcheon, M. Elimelech, *Desalination* **2006**, 197, 1.

[66] V. Gekas, B. Hallström, *J. Membr. Sci.* **1987**, 30, 153.
