# Supplementary material for: Customizable Manufacturing of Polyamide Membranes with Programmable Layers and High Permselectivity by Electrospray Printer
Source: Adv Sci (Weinh). 2025 Sep 12;12(45):e09127. doi: 10.1002/advs.202509127 (PMC12677634; doi:10.1002/advs.202509127)
Supplement: Supplementary file 2 — Supplemental Data [file ADVS-12-e09127-s005.docx]

Code S1 Slicing Program

This file is the **Slicing Program** for programmable electrospray 3D printer. It was written by Xieyang XU in Python and includes 19 .py files:

[**main.py** 2](#_Toc197461942)

[**pg_base.py** 8](#_Toc197461943)

[**pg_line.py** 14](#_Toc197461944)

[**pg_ray.py** 15](#_Toc197461945)

[**pg_segment.py** 16](#_Toc197461946)

[**pg_polyline.py** 18](#_Toc197461947)

[**pg_algo.py** 21](#_Toc197461948)

[**di_base.py** 25](#_Toc197461949)

[**dp_base.py** 29](#_Toc197461950)

[**dp_blueprint.py** 32](#_Toc197461951)

[**dt_blueprints.py** 35](#_Toc197461952)

[**dt_character.py** 39](#_Toc197461953)

[**bs_base.py** 44](#_Toc197461954)

[**bs_vertex.py** 46](#_Toc197461955)

[**bs_linkvertex.py** 49](#_Toc197461956)

[**cl_adaptor.py** 52](#_Toc197461957)

[**pp_base.py** 53](#_Toc197461958)

[**pp_genpath.py** 56](#_Toc197461959)

[**pp_splitregion.py** 58](#_Toc197461960)

**main.py**

# -*- coding:UTF-8 -*-

# By

# gcode_output

from pp_genpath import *

class GcodeOutput:

def __init__(self, path_list, sol_list, character, solution, process, printer):

self.path_list = path_list

self.sol_list = sol_list

self.character = character

self.solution = solution

self.process = process

self.printer = printer

self.list_height = len(sol_list)

self.list_width = len(sol_list[0]) if self.list_height > 0 else 0

self.rd_list = [[[] for _ in range(self.list_width)] for _ in range(self.list_height)]

file_path = '%s%s.gcode' % ('C:/Users/Xu/Documents/Gcode/', self.character.name)

self.file = open(file_path, 'w', encoding='utf-8')

def calculate_rotation_distance(self):

e0, e1, e2, e3, e4 = 60, -1, -1, -1, -1 #

water_mpd = self.process.pushSpeed[0] / self.process.pushSpeed[1]

pst_mpd = self.solution.pst_MPD()

pst_tmc = self.solution.pst_TMC()

pst_solvent = 1 - self.solution.pst_MPD() - self.solution.pst_TMC()

for m in range(self.list_height):

for n in range(self.list_width):

if m == 0:

e1 = format(60 / water_mpd, '.5f')

else:

e1 = -1

if self.sol_list[m][n][1] >= 1:

e0 = format(60 / self.sol_list[m][n][1] * 255 / pst_tmc, '.5f')

else:

e0 = -1

if self.sol_list[m][n][2] >= 1:

e2 = format(60 / self.sol_list[m][n][2] * 255 / pst_mpd, '.5f')

else:

e2 = -1

if self.sol_list[m][n][3] < 1:

e3 = -1

e4 = format(60 / pst_solvent, '.5f')

elif self.sol_list[m][n][3] > 254:

e3 = format(60 / pst_solvent, '.5f')

e4 = -1

else:

e3 = format(60 / pst_solvent / self.sol_list[m][n][3] * 255, '.5f')

e4 = format(60 / pst_solvent / (255 - self.sol_list[m][n][3]) * 255, '.5f')

self.rd_list[m][n] = [e0, e1, e2, e3, e4]

def switch_extruder(self, m, n):

rds = self.rd_list[m][n]

extruder_name = ['extruder', 'solution_water', 'solution_organic2', 'solution_organic3', 'solution_organic4']

codes = []

code0 = "SET_EXTRUDER_ROTATION_DISTANCE EXTRUDER=%s DISTANCE=%s " % (str(extruder_name[0]), str(rds[0]))

codes.append(code0)

for ext in range(1, 5):

if rds[ext] == -1:

code = "SYNC_EXTRUDER_MOTION EXTRUDER=%s MOTION_QUEUE= " % (extruder_name[ext])

codes.append(code)

else:

code = ("SYNC_EXTRUDER_MOTION EXTRUDER=%s MOTION_QUEUE=%s \n\

SET_EXTRUDER_ROTATION_DISTANCE EXTRUDER=%s DISTANCE=%s"

% (extruder_name[ext], extruder_name[0], extruder_name[ext], str(rds[ext])))

codes.append(code)

switch_code = " %s\n%s\n%s\n%s\n%s\n;push switch" % (codes[0], codes[1], codes[2], codes[3], codes[4])

return switch_code

def print_path_spray(self, point1, point2):

dpm = self.character.dpm

width = self.character.width()

dsx = self.printer.startPoint[0]

dsy = self.printer.startPoint[1]

print_speed = self.process.printSpeed * 60

push_speed = self.process.pushSpeed

ld = point2.distance(point1)

pntx = format(point1.y / dpm + dsx, '.3f')

pnty = format((width - point1.x) / dpm + dsy, '.3f')

pnte = format(ld / print_speed * push_speed[1], '.5f')

print_code = "G1 F%s X%s Y%s E%s" % (str(print_speed), str(pntx), str(pnty), str(pnte))

return print_code

def free_path_spray(self, point1, point2):

dpm = self.character.dpm

width = self.character.width()

dsx = self.printer.startPoint[0]

dsy = self.printer.startPoint[1]

free_speed = self.process.freeSpeed * 60

push_speed = self.process.pushSpeed

ld = point2.distance(point1)

pntx = format(point1.y / dpm + dsx, '.3f')

pnty = format((width - point1.x) / dpm + dsy, '.3f')

pnte = format(ld / free_speed * push_speed[1], '.5f')

free_code = "G1 F%s X%s Y%s E%s" % (str(free_speed), str(pntx), str(pnty), str(pnte))

return free_code

def write_code_spray(self):

file = self.file

file.write(str(self.start_code_spray()))

pnt_link = Point2D()

for m in range(self.list_height):

for n in range(self.list_width):

paths = self.path_list[m][n]

region_nmb = 1

file.write('\n\n;' + str(m + 1) + 'layer ' + str(n + 1) + 'color' + '\n')

file.write(self.switch_extruder(m, n))

for path in paths:

file.write('\n;area'+str(region_nmb)+'\n')

for i, pnt1 in enumerate(path.points):

pnt2 = path.points[i-1]

if i == 0:

file.write(str(self.free_path_spray(pnt1, pnt_link))+'\n')

elif path.points[i-1].y == path.points[i].y:

file.write(str(self.print_path_spray(pnt1, pnt2))+'\n')

else:

file.write(str(self.free_path_spray(pnt1, pnt2))+'\n')

if i == len(path.points):

pnt_link = pnt1

else:

pass

region_nmb += 1

file.write(str(self.end_code_spray()))

file.close()

def start_code(self):

startcode = ';Writed by *** \n\

M140 S30 ;*** \n\

M105 ;*** \n\

M190 S30 ;*** \n\

M107 S0 ;*** \n\

M82 ;*** \n\

G90 ;*** \n\

G28 ;Hoom \n\

G0 F3000 X30 Y230 Z12 ;*** \n\

G4 P300000 ;*** \n\

G0 F300 X30 Y90 Z12 ;*** \n\

G0 F300 X30 Y230 ;*** \n\

G0 F300 X30 Y90 Z12 ;*** \n\

G0 F300 X30 Y230 ;*** \n\

G0 F300 X30 Y90 Z12 ;*** \n\

M107 ;*** \n\

\n;***'

return startcode

def start_code_spray(self):

startcode = ';Writed by *** \n\

M140 S60 ;*** \n\

M190 S60 ;*** \n\

G90 ;*** \n\

M83 ;*** \n\

G28 ;Hoom \n\

G0 F3000 X30 Y190 Z12 ;*** \n\

G4 P180000 ;*** \n\

G0 F300 X30 Y50 Z12 ;*** \n\

G0 F300 X30 Y190 ;*** \n\

G0 F300 X30 Y50 Z12 ;*** \n\

G0 F300 X30 Y190 ;*** \n\

G0 F300 X30 Y50 Z12 ;*** \n\

\n;***'

return startcode

def end_code(self):

endcode = '\n;*** \n\

M140 S0 ;*** \n\

G91 ;*** \n\

G0 F3000 Y5 ;*** \n\

G90 ;*** \n\

G0 F3000 X30 ;*** \n\

G0 F3000 Y230 ;*** \n\

G0 F3000 Z12 ;*** \n\

M106 S0 ;Turn-off fan *** \n\

M104 S0 ;Turn-off hotend *** \n\

M140 S0 ;Turn-off bed *** \n\

M82 ;*** \n\

M104 S0 ;*** \n\

;End of Gcode \n'

return endcode

def end_code_spray(self):

endcode = '\n;*** \n\

M140 S0 ;*** \n\

G91 ;*** \n\

G0 F3000 Y5 ;*** \n\

G90 ;*** \n\

G0 F3000 X30 ;*** \n\

G0 F3000 Y190 ;*** \n\

G0 F3000 Z12 ;*** \n\

M104 S0 ;Turn-off hotend *** \n\

M140 S0 ;Turn-off bed *** \n\

M82 ;*** \n\

;End of Gcode \n'

return endcode

if __name__ == '__main__':

blueprint = Blueprints('membrane_printer_test', 'blueprints/*.jpg', 5)

solution = Solution([0.5, 10], [0.025, 0.5])

process = Process()

printer = Printer()

bptreat = Blueprintstreat(blueprint, solution, process)

bptreat.whole_treat()

sol_list = bptreat.sol_list

for m in range(bptreat.layers_number):

for n in range(bptreat.area_number):

surface_list = bptreat.sfc_list[m][n]

border_list = bptreat.bdr_list[m][n]

vertexes = Vertexes(surface_list, border_list)

vertexes.create_vertexes()

border = vertexes.bdr

vertex = vertexes.vtx

linkvertex = Linkvertex(border, vertex)

linkvertex.link_vertex()

contours = linkvertex.contours

interval = process.routeSpacing * blueprint.dpm

gen_path = GenPath(contours, interval, 0)

paths = gen_path.generate_angle_zero()

bptreat.path_list[m][n] = paths

path_list = bptreat.path_list

gcode_output = GcodeOutput(path_list, sol_list, blueprint, solution, process, printer)

gcode_output.calculate_rotation_distance()

gcode_output.write_code_spray()

**pg_base.py**

# -*- coding:UTF-8 -*-

# By

# plane_geometry

import math

epsilon = 1e-7

class Point2D:

def __init__(self, x=0.0, y=0.0, w=1.0):

self.x = x

self.y = y

self.w = w

def __str__(self):

return 'Point2D: %s, %s' % (self.x, self.y)

def clone(self):

return Point2D(self.x, self.y)

def point_to(self, other):

return Vector2D(other.x - self.x, other.y - self.y, other.w - self.w)

def translate(self, vec):

self.x = self.x + vec.dx

self.y = self.y + vec.dy

self.w = self.w + vec.dw

def translated(self, vec):

return Point2D(self.x + vec.dx, self.y + vec.dy, self.w + vec.dw)

def multiplied(self, m):

x = self.x * m.a[0][0] + self.y * m.a[1][0] + self.w * m.a[2][0]

y = self.x * m.a[0][1] + self.y * m.a[1][1] + self.w * m.a[2][1]

return Point2D(x, y)

def distance(self, other):

return math.sqrt(self.distance_square(other))

def distance_square(self, other):

return self.point_to(other).length_square()

def middle(self, other):

return Point2D((self.x + other.x)/2.0, (self.y + other.y)/2.0, (self.w + other.w)/2.0)

def is_coincide(self, other):

return self.distance_square(other) < epsilon

def is_identical(self, other):

return self.x == other.x and self.y == other.y

def __add__(self, vec):

return self.translated(vec)

def __sub__(self, other):

if isinstance(other, Point2D):

return other.point_to(self)

else:

return self.translated(other.reversed())

def __mul__(self, m):

return self.multiplied(m)

class Vector2D:

def __init__(self, dx=0.0, dy=0.0, dw=0.0):

self.dx = dx

self.dy = dy

self.dw = dw

def __str__(self):

return 'Vector2D: %s, %s' % (self.dx, self.dy)

def clone(self):

return Vector2D(self.dx, self.dy)

def reverse(self):

self.dx = -self.dx

self.dy = -self.dy

def dot_product(self, other):

return self.dx * other.dx + self.dy * other.dy

def cross_product(self, other):

return self.dx * other.dy - self.dy * other.dx

def amplify(self, f):

self.dx = self.dx * f

self.dy = self.dy * f

def amplified(self, f):

vcta = Vector2D(self.dx * f, self.dy * f)

return vcta

def reversed(self):

return Vector2D(-self.dx, -self.dy)

def length(self):

return math.sqrt(self.length_square())

def length_square(self):

return self.dx * self.dx + self.dy * self.dy

def normalize(self):

lov = self.length()

self.dx = self.dx / lov

self.dy = self.dy / lov

def normalized(self):

lov = self.length()

return Vector2D(self.dx/lov, self.dy/lov)

def is_zero_vector(self):

return self.length_square() == 0.0

def multiplied(self, m):

x = self.dx * m.a[0][0] + self.dy * m.a[1][0] + self.dw * m.a[2][0]

y = self.dx * m.a[0][1] + self.dy * m.a[1][1] + self.dw * m.a[2][1]

return Vector2D(x, y)

def is_parallel(self, other):

return self.cross_product(other) == 0

def get_angle(self, vec):

v1 = self.normalized()

v2 = vec.normalized()

dot = v1.dot_product(v2)

if dot < -1.0:

dot = -1.0

if dot > 1.0:

dot = 1.0

return math.acos(dot)

def get_angle_x(self):

rad = self.get_angle(Vector2D(1.0, 0))

if self.dy < 0:

rad = 2 * math.pi - rad

return rad

def get_ortho_vector(self):

if self.dx == 0:

return Vector2D(1.0, 0.0)

else:

return Vector2D(-self.dy / self.dx, 1.0).normalized()

def __add__(self, other):

return Vector2D(self.dx + other.dx, self.dy + other.dy)

def __sub__(self, other):

return Vector2D(self.dx - other.dx, self.dy - other.dy)

def __mul__(self, m):

return self.multiplied(m)

class Matrix2D:

def __init__(self):

self.a = [[1.0, 0.0, 0.0], [0.0, 1.0, 0.0], [0.0, 0.0, 1.0]]

def __str__(self):

return "Matrix2D: \n%s\n%s\n%s" % (self.a[0], self.a[1], self.a[2])

def make_identical(self):

self.a = [[1.0, 0.0, 0.0], [0.0, 1.0, 0.0], [0.0, 0.0, 1.0]]

def multiplied(self, other):

m = Matrix2D()

for i in range(3):

for j in range(3):

m.a[i][j] = self.a[i][0] * other.a[0][j] \

+ self.a[i][1] * other.a[1][j] \

+ self.a[i][2] * other.a[2][j]

return m

def get_determinant(self):

pass

def get_reverse_matrix(self):

pass

@staticmethod

def create_translate_matrix(dx, dy):

m = Matrix2D()

m.a[2][0] = dx

m.a[2][1] = dy

return m

@staticmethod

def create_scale_matrix(sx, sy):

m = Matrix2D()

m.a[0][0] = sx

m.a[1][1] = sy

return m

@staticmethod

def create_rotate_matrix(axis, angle):

m = Matrix2D()

sin = math.sin(angle)

cos = math.cos(angle)

if axis == "X" or axis == "x":

m.a[2][2] = -1

elif axis == "Y" or axis == "y":

m.a[1][1] = -1

elif axis == "Z" or axis == "z":

m.a[0][0], m.a[0][1], m.a[1][0], m.a[1][1] = cos, sin, -sin, cos

return

@staticmethod

def create_mirror_matrix(point, normal):

pass

def __mul__(self, other):

return self.multiplied(other)

def __add__(self, other):

m = Matrix2D()

for i in range(3):

for j in range(4):

m.a[i][j] = self.a[i][j] + other.a[i][j]

return m

def __sub__(self, other):

m = Matrix2D()

for i in range(3):

for j in range(4):

m.a[i][j] = self.a[i][j] - other.a[i][j]

return m

if __name__ == '__main__':

pass

**pg_line.py**

# -*- coding:UTF-8 -*-

# By

# plane_geometry

from pg_base import *

class Line: #

def __init__(self, pot, vec):

self.pot = pot.clone()

self.vec = vec.clone().normalized()

def __str__(self):

return "Line\npot %s\nvec %s\n" % (str(self.pot), str(self.vec))

if __name__ == '__main__':

p = Point2D(1, 2)

v = Vector2D(4, 5)

ln = Line(p, v)

print(ln)

p.x = 0

print(ln)

**pg_ray.py**

# -*- coding:UTF-8 -*-

# By

# plane_geometry

from pg_base import *

class Ray:

def __init__(self, pot, vec):

self.pot = pot.clone()

self.vec = vec.clone().normalized()

def __str__(self):

return "Ray\npot %s\nvec %s\n" % (str(self.pot), str(self.vec))

if __name__ == '__main__':

p = Point2D(1, 2)

v = Vector2D(4, 5)

ln = Ray(p, v)

print(ln)

p.x = 0

ln = Ray(p, v)

print(ln)

**pg_segment.py**

# -*- coding:UTF-8 -*-

# By

# plane_geometry

from pg_base import *

class Segment:

def __init__(self, pot1, pot2):

self.pot1 = pot1.clone()

self.pot2 = pot2.clone()

def __str__(self):

return "Segment\npot1 %s\npot2 %s\n" % (str(self.pot1), str(self.pot2))

def length(self):

return self.pot1.distance(self.pot2)

def direction(self):

return self.pot1.point_to(self.pot2)

def swap(self):

self.pot1, self.pot2 = self.pot2, self.pot1

def multiply(self, m):

self.pot1 = self.pot1.multiplied(m)

self.pot2 = self.pot2.multiplied(m)

def multiplied(self, m):

seg = Segment(self.pot1, self.pot2)

seg.multiply(m)

return seg

def y_min(self):

return min(self.pot1.y, self.pot2.y)

def y_max(self):

return max(self.pot1.y, self.pot2.y)

if __name__ == '__main__':

p = Point2D(1, 2)

v = Point2D(4, 5)

ln = Segment(p, v)

print(ln)

p.x = 0

ln = Segment(p, v)

print(ln)

**pg_polyline.py**

# -*- coding:UTF-8 -*-

# By

# plane_geometry

from pg_base import *

class Polyline:

def __init__(self):

self.points = []

def __str__(self):

pass

def clone(self):

poly = Polyline()

for pot in self.points:

poly.points.append(pot.clone())

return poly

def count(self):

return len(self.points)

def add_point(self, pot):

self.points.append(pot)

def add_pnt_tpl(self, pnt_tpl):

self.points.append(Point2D(pnt_tpl[0], pnt_tpl[1]))

def radd_point(self, pot):

self.points.insert(0, pot)

def remove_point(self, index):

return self.points.pop(index)

def point(self, index):

return self.points[index]

def start_point(self):

return self.points[0]

def end_point(self):

return self.points[-1]

def is_close(self):

if self.count() <= 2:

return False

else:

return self.start_point().is_coincide(self.end_point())

def reverse(self):

sz = self.count()

for i in range(int(sz/2)):

self.points[i], self.points[sz-1-i] = self.points[sz-1-i], self.points[i]

def get_area(self):

area = 0.0

for i in range(self.count()):

area += 0.5*(self.points[i].x*self.points[i+1].y-self.points[i].y*self.points[i+1].x)

return area

def make_ccw(self):

if self.get_area() < 0:

self.reverse()

def make_cw(self):

if self.get_area() > 0:

self.reverse()

def is_ccw(self):

return self.get_area() > 0

def translate(self, vec):

for i in range(len(self.points)):

self.points[i].translate(vec)

def append_segment(self, seg):

if self.count() == 0:

self.points.append(seg.pot1)

self.points.append(seg.pot2)

else:

if seg.pot1.is_conincide(self.end_point()):

self.add_point(seg.pot2)

elif seg.pot2.is_conincide(self.end_point()):

self.add_point(seg.pot1)

elif seg.pot1.is_conincide(self.start_point()):

self.radd_point(seg.pot2)

elif seg.pot2.is_conincide(self.start_point()):

self.radd_point(seg.pot1)

else:

return False

return True

def multiply(self, m):

for pt in self.points:

pt.multiply(m)

def multiplied(self, m):

poly = Polyline()

for pt in self.points:

poly.add_point(pt * m)

return poly

def write_polyline(path, polyline: Polyline):

f = None

try:

f = open(path, 'w')

f.write('%s\n' % polyline.count())

for pot in polyline.points:

txt = "%s, %s" % (pot.x, pot.y)

f.write(txt)

except Exception as ex:

print(ex)

finally:

if f:

f.close()

**pg_algo.py**

# -*- coding:UTF-8 -*-

# By

# plane_geometry

from pg_line import *

from pg_ray import *

from pg_segment import *

from pg_polyline import *

a = Point2D()

def near_zero(e):

return math.fabs(e) < epsilon

def distance(obj1, obj2):

if isinstance(obj1, Point2D) and isinstance(obj2, Line):

P, Q, V = obj2.pot, obj1, obj2.vec

t = P.point_to(Q).dot_product(V)

R = P + V.amplifed(t)

d = Q.distance(R)

return d

elif isinstance(obj1, Point2D) and isinstance(obj2, Ray):

P, Q, V = obj2.pot, obj1, obj2.vec

t = P.point_to(Q).dot_product(V)

if t >= 0:

R = P + V.amplifed(t)

return Q.distance(R)

else:

return Q.distance(P)

elif isinstance(obj1, Point2D) and isinstance(obj2, Segment):

Q, P, P1, V = obj1, obj2.pot1, obj2.pot2, obj2.direction().normalized()

L = obj2.length()

t = P.point_to(Q).dot_product(V)

if t <= 0:

return Q.distance(P)

elif t >= L:

return Q.distance(P1)

else:

R = P + V.amplifed(t)

pass

def intersect_line_line(line1 : Line, line2 : Line):

P1, V1, P2, V2 = line1.pot, line1.vec, line2.pot, line2.vec

P1P2 = P1.point_to(P2)

deno = V1.dy * V2.dx - V1.dx * V2.dy

if deno != 0:

t1 = -(-P1P2.dy*V2.dx + P1P2.dx*V2.dy)/deno

t2 = -(-P1P2.dy*V1.dx + P1P2.dx*V1.dy)/deno

return P1 + V1.amplified(t1), t1, t2

else:

return None, 0, 0

def intersect(obj1, obj2):

if isinstance(obj1, Line) and isinstance(obj2, Line):

P, t1, t2 = intersect_line_line(obj1, obj2)

return P

elif isinstance(obj1, Segment) and isinstance(obj2, Segment):

line1 = Line(obj1.pot1, obj1.direction())

line2 = Line(obj2.pot1, obj2.direction())

P, t1, t2 = intersect_line_line(line1, line2)

if P is not None:

if t1 >= 0 and t1 <= obj1.length() and t2 >= 0 and t2 <= obj2.length():

return P

return None

elif isinstance(obj1, Line) and isinstance(obj2, Segment):

line1 = obj1

line2 = Line(obj2.pot1, obj2.direction())

P, t1, t2 = intersect_line_line(line1, line2)

if P is not None and t2 >= 0 and t2 <= obj2.length():

return P

return None

elif isinstance(obj1, Line) and isinstance(obj2, Ray):

pass

elif isinstance(obj1, Ray) and isinstance(obj2, Segment):

pass

elif isinstance(obj1, Ray) and isinstance(obj2, Ray):

pass

def point_on_ray(p: Point2D, ray: Ray):

v = ray.pot.point_to(p)

if v.dot_product(ray.vec) >=0 and v.cross_product(ray.vec) == 0:

return True

return False

def point_in_polygon(p: Point2D, polygon: Polyline):

passcount = 0

ray = Ray(p, Vector2D(1, 0))

segments = []

for i in range(polygon.count()-1):

seg = Segment(polygon.point(i), polygon.point(i+1))

segments.append(seg)

for seg in segments:

line1 = Line(ray.pot, ray.vec)

line2 = Line(seg.pot1, seg.direction())

P, t1, t2 = intersect_line_line(line1, line2)

if P is not None:

if near_zero(t1):

return -1

elif seg.pot1.y != p.y and seg.pot2 != p.y and t1 > 0 and t2 > 0 and t2 < seg.length():

passcount += 1

upsegments, downsegment = [], []

for seg in segments:

if seg.pot1.is_identical(ray.pot) or seg.pot2.is_identical(ray.pot):

return -1

elif point_on_ray(seg.pot1, ray) ^ point_on_ray(seg.pot2, ray):

if seg.pot1.y >= p.y and seg.pot2.y >= p.y:

upsegments.append(seg)

elif seg.pot1.y <= p.y and seg.pot2.y <= p.y:

downsegment.append(seg)

passcount += min(len(upsegments), len(downsegment))

if passcount % 2 == 1:

return 1

return 0

def adjust_polygon_dirs(polygons):

for i in range(len(polygons)):

pt = polygons[i].start_point()

inside_count = 0

for j in range(len(polygons)):

if i == j:

continue

rest_poly = polygons[j]

if 1 == point_in_polygon(pt, rest_poly):

inside_count += 1

if inside_count % 2 == 0:

polygons[i].make_ccw()

else:

polygons[i].make_cw()

def rotatePolygons(polygons, angle, center = None):

dx = 0 if center is None else center.x

dy = 0 if center is None else center.y

mt = Matrix2D.create_translate_matrix(-dx, -dy)

mr = Matrix2D.create_rotate_matrix('Z', angle)

mb = Matrix2D.create_translate_matrix(dx, dy)

m = mt + mr + mb

new_polys = []

for poly in polygons:

new_polys.append(poly.multiplied(m))

return new_polys

if __name__ == '__main__':

pass

**di_base.py**

# -*- coding:UTF-8 -*-

# By

# data_input

import glob

import cv2 as cv

import matplotlib.pyplot as plt

class Character:

def __init__(self, nm, og, dpm=16.0):

self.name = nm

self.fileName = og

self.org = cv.imread(og)

self.dpm = dpm

def __str__(self):

return "Characteristic\nName: %s\nFileName: %s\nDots/mm: %s\n" \

% (str(self.name), str(self.fileName), str(self.dpm))

def cut_filename(self):

return self.fileName[:-4]

def show(self):

plt.rcParams['font.sans-serif'] = ['SimHei']

titles = self.name

images = self.org

plt.subplot(1, 1, 1), plt.imshow(images, 'gray')

plt.title(titles)

plt.xticks(), plt.yticks()

plt.show()

def height(self):

return self.org.shape[0]

def width(self):

return self.org.shape[1]

class Indicator:

def __init__(self, rgb, cri):

self.RGB = rgb

self.CRI = cri

self.std = [[0, 0, 0, 0], [100, 100, 100, 100], [200, 200, 200, 200], [255, 255, 255, 255]]

def __str__(self):

return "Indicator\nRGB: %s\nCRI: %s\n" % (str(self.RGB), str(self.CRI))

def rgb_sum(self):

return self.RGB[0] + self.RGB[1] + self.RGB[2]

def rw(self):

return self.RGB[2] / self.rgb_sum()

def gw(self):

return self.RGB[1] / self.rgb_sum()

def bw(self):

return self.RGB[0] / self.rgb_sum()

class Blueprints:

def __init__(self, nm, og, dpm=16.0):

self.name = nm

self.folderPath = og

self.bluePrints = glob.glob(og)

self.base = cv.imread(self.bluePrints[0])

self.fileNum = len(self.bluePrints)

self.dpm = dpm

def __str__(self):

return "Characteristic\nName: %s\nFileName: %s\nDots/mm: %s\n" \

% (str(self.name), str(self.folderPath), str(self.dpm))

def cut_filename(self):

filename = []

for i in self.bluePrints:

cut = i[:-4]

filename.append(cut)

return filename

def show(self):

for i in self.bluePrints:

plt.rcParams['font.sans-serif'] = ['SimHei']

titles = i

images = cv.imread(i)

plt.subplot(1, 1, 1), plt.imshow(images, 'gray')

plt.title(titles)

plt.xticks(), plt.yticks()

plt.show()

def height(self):

return self.base.shape[0]

def width(self):

return self.base.shape[1]

class Solution:

def __init__(self, msl, hdd):

self.msl = msl

self.hdd = hdd

self.std = [[0, 255, 255, 255], [100, 100, 100, 100], [200, 200, 200, 200], [255, 255, 255, 255]]

def __str__(self):

return "Solution\nMother Solution: %s\n100 standard: %s\n" % (str(self.msl), str(self.hdd))

def pst_TMC(self):

return self.hdd[0] / self.msl[0]

def pst_MPD(self):

return self.hdd[1] / self.msl[1]

class Process:

def __init__(self, sprayr=5.0, temp=298.15, method=0, routes=1, fs=100, ps=10):

self.pushSpeed = [0.10, 0.15]

self.sprayRadius = sprayr

self.temperature = temp

self.method = method

self.routeSpacing = routes

self.freeSpeed = fs

self.printSpeed = ps

def __str__(self):

prc = ["Process parameter",

"Push speed: %s mm/min" % str(self.pushSpeed),

"Spray radius: %s mm" % str(self.sprayRadius),

"Temperature: %s K" % str(self.temperature),

"Move method: %s method" % str(self.method),

"Route spacing: %s mm" % str(self.routeSpacing),

"Print speed: %s mm/s" % str(self.printSpeed),

"Speed(translate): %s mm/s" % str(self.freeSpeed)]

return str('\n'.join(prc))

class Printer:

def __init__(self, dl=(235, 235, 250), sl=100, sp=(0, 0, 12), nz=(0, -14, 8), mn=(-18, 0, 0)):

self.diLimit = dl

self.speedLimit = sl

self.startPoint = sp

self.nozzleOffset = nz

self.multiNozzle = mn

def __str__(self):

prt = ["Printer parameter",

"Dimension limit: %s mm" % str(self.diLimit),

"Speed limit: %s mm/s" % str(self.speedLimit),

"Start point: %s mm" % str(self.startPoint),

"Nozzle offset: %s mm" % str(self.nozzleOffset),

"MultiNozzle: %s mm" % str(self.multiNozzle)]

return str('\n'.join(prt))

if __name__ == '__main__':

pass

**dp_base.py**

# -*- coding:UTF-8 -*-

# By

# data_pretreat

import numpy as np

from pg_base import *

from di_base import *

class Charactertreat:

def __init__(self, cha, idt, prc):

self.cha = cha

self.idt = idt

self.prc = prc

self.height = int(cha.height())

self.width = int(cha.width())

self.spraydots = int(prc.sprayRadius * cha.dpm)

self.routedots = int(cha.dpm * prc.routeSpacing)

self.thresh_value = int(0)

# 新建预处理中间文件

self.gray = np.zeros((self.height, self.width, 3), np.uint8)

self.filter = np.zeros((self.height, self.width, 3), np.uint8)

self.sampling = np.zeros((self.height, self.width, 3), np.uint8)

self.thresh = np.zeros((self.height, self.width, 3), np.uint8)

self.smooth = np.zeros((self.height, self.width, 3), np.uint8)

self.laplacian = np.zeros((self.height, self.width, 3), np.uint8)

self.border = np.zeros((self.height, self.width, 3), np.uint8)

self.sfc_list = []

self.bdr_list = []

def idt_gray(self):

rw1 = self.idt.rw()

gw1 = self.idt.gw()

bw1 = self.idt.bw()

for i in range(self.height):

for j in range(self.width):

gray = 255 - (rw1 * self.cha.org[i, j][2] + gw1 * self.cha.org[i, j][1] + bw1 * self.cha.org[i, j][0])

log_gray = 255 - 45.9 * np.log(1.0 + gray)

self.gray[i, j] = np.uint8(log_gray)

def gray_filter(self):

self.filter = cv.blur(self.gray, (int(self.spraydots / 2), int(self.spraydots / 2)))

def filter_samp(self):

sampdots_h = int(self.height / self.routedots)

sampdots_w = int(self.width / self.routedots)

for i in range(sampdots_h):

y = i * self.routedots

for j in range(sampdots_w):

x = j * self.routedots

g = self.filter[y, x]

for n in range(self.routedots):

for m in range(self.routedots):

self.sampling[y + n, x + m] = np.uint8(g)

def samp_thresh(self):

ret, self.thresh = cv.threshold(self.sampling, self.thresh_value, 255, cv.THRESH_BINARY)

def thresh_smooth(self):

self.smooth = self.thresh.copy()

for m in range(5):

k_open = np.ones((m + self.routedots, m + self.routedots), np.uint8)

self.smooth = cv.morphologyEx(self.smooth, cv.MORPH_OPEN, k_open)

k_close = np.ones((m + self.routedots, m + self.routedots), np.uint8)

self.smooth = cv.morphologyEx(self.smooth, cv.MORPH_CLOSE, k_close)

def smooth_lap(self):

dst = cv.Laplacian(self.smooth, cv.CV_16S, ksize=3)

self.laplacian = cv.convertScaleAbs(dst)

def show_border(self):

self.border = self.cha.org.copy()

for y in range(self.height):

for x in range(self.width):

if self.laplacian[y, x][0] == 0 and self.laplacian[y, x][1] == 0 and self.laplacian[y, x][2] == 0:

pass

else:

self.border[y, x] = [0, 0, 255]

def create_sfr(self):

sfc = [[0 for _ in range(self.width)] for _ in range(self.height)]

for y in range(self.height):

for x in range(self.width):

sfc[y][x] = int(self.smooth[y, x][0] / 255 + epsilon)

self.sfc_list.append(sfc)

def create_bdr(self):

bdr = [[0 for _ in range(self.width)] for _ in range(self.height)]

for y in range(self.height):

for x in range(self.width):

bdr[y][x] = int(self.laplacian[y, x][0] / 255 + epsilon)

self.bdr_list.append(bdr)

def whole_pretreat(self):

self.idt_gray()

cv.imwrite('gray.jpg', self.gray, [int(cv.IMWRITE_JPEG_QUALITY), 100])

self.gray_filter()

cv.imwrite('filter.jpg', self.filter, [int(cv.IMWRITE_JPEG_QUALITY), 100])

self.filter_samp()

cv.imwrite('sampling.jpg', self.sampling, [int(cv.IMWRITE_JPEG_QUALITY), 100])

ls = len(self.idt.std)

for li in range(ls):

self.thresh_value = self.idt.std[li]

self.samp_thresh()

cv.imwrite('thresh' + str(li) + '.jpg', self.thresh, [int(cv.IMWRITE_JPEG_QUALITY), 100])

self.thresh_smooth()

cv.imwrite('smooth' + str(li) + '.jpg', self.smooth, [int(cv.IMWRITE_JPEG_QUALITY), 100])

self.smooth_lap()

cv.imwrite('lap' + str(li) + '.jpg', self.laplacian, [int(cv.IMWRITE_JPEG_QUALITY), 100])

self.create_sfr()

self.create_bdr()

if __name__ == '__main__':

pass

**dp_blueprint.py**

# -*- coding:UTF-8 -*-

# By

# data_pretreat

import numpy as np

from pg_base import *

from di_base import *

class Pretreat:

def __init__(self, cha, idt, prc):

self.cha = cha

self.idt = idt

self.prc = prc

self.height = int(cha.height())

self.width = int(cha.width())

self.spraydots = int(prc.sprayRadius * cha.dpm)

self.routedots = int(cha.dpm * prc.routeSpacing)

self.thresh_value = int(0)

self.gray = np.zeros((self.height, self.width, 3), np.uint8)

self.filter = np.zeros((self.height, self.width, 3), np.uint8)

self.sampling = np.zeros((self.height, self.width, 3), np.uint8)

self.thresh = np.zeros((self.height, self.width, 3), np.uint8)

self.smooth = np.zeros((self.height, self.width, 3), np.uint8)

self.laplacian = np.zeros((self.height, self.width, 3), np.uint8)

self.border = np.zeros((self.height, self.width, 3), np.uint8)

self.sfc_list = []

self.bdr_list = []

def idt_gray(self):

rw1 = self.idt.rw()

gw1 = self.idt.gw()

bw1 = self.idt.bw()

for i in range(self.height):

for j in range(self.width):

gray = 255 - (rw1 * self.cha.org[i, j][2] + gw1 * self.cha.org[i, j][1] + bw1 * self.cha.org[i, j][0])

log_gray = 255 - 45.9 * np.log(1.0 + gray)

self.gray[i, j] = np.uint8(log_gray)

def gray_filter(self):

self.filter = cv.blur(self.gray, (int(self.spraydots / 2), int(self.spraydots / 2)))

def filter_samp(self):

sampdots_h = int(self.height / self.routedots)

sampdots_w = int(self.width / self.routedots)

for i in range(sampdots_h):

y = i * self.routedots

for j in range(sampdots_w):

x = j * self.routedots

g = self.filter[y, x]

for n in range(self.routedots):

for m in range(self.routedots):

self.sampling[y + n, x + m] = np.uint8(g)

def samp_thresh(self):

ret, self.thresh = cv.threshold(self.sampling, self.thresh_value, 255, cv.THRESH_BINARY)

def thresh_smooth(self):

self.smooth = self.thresh.copy()

for m in range(5):

k_open = np.ones((m + self.routedots, m + self.routedots), np.uint8)

self.smooth = cv.morphologyEx(self.smooth, cv.MORPH_OPEN, k_open)

k_close = np.ones((m + self.routedots, m + self.routedots), np.uint8)

self.smooth = cv.morphologyEx(self.smooth, cv.MORPH_CLOSE, k_close)

def smooth_lap(self):

dst = cv.Laplacian(self.smooth, cv.CV_16S, ksize=3)

self.laplacian = cv.convertScaleAbs(dst)

def show_border(self):

self.border = self.cha.org.copy()

for y in range(self.height):

for x in range(self.width):

if self.laplacian[y, x][0] == 0 and self.laplacian[y, x][1] == 0 and self.laplacian[y, x][2] == 0:

pass

else:

self.border[y, x] = [0, 0, 255]

def create_sfr(self):

sfc = [[0 for _ in range(self.width)] for _ in range(self.height)]

for y in range(self.height):

for x in range(self.width):

sfc[y][x] = int(self.smooth[y, x][0] / 255 + epsilon)

self.sfc_list.append(sfc)

def create_bdr(self):

bdr = [[0 for _ in range(self.width)] for _ in range(self.height)]

for y in range(self.height):

for x in range(self.width):

bdr[y][x] = int(self.laplacian[y, x][0] / 255 + epsilon)

self.bdr_list.append(bdr)

def whole_pretreat(self):

self.idt_gray()

cv.imwrite('gray.jpg', self.gray, [int(cv.IMWRITE_JPEG_QUALITY), 100])

self.gray_filter()

cv.imwrite('filter.jpg', self.filter, [int(cv.IMWRITE_JPEG_QUALITY), 100])

self.filter_samp()

cv.imwrite('sampling.jpg', self.sampling, [int(cv.IMWRITE_JPEG_QUALITY), 100])

ls = len(self.idt.std)

for li in range(ls):

self.thresh_value = self.idt.std[li]

self.samp_thresh()

cv.imwrite('thresh' + str(li) + '.jpg', self.thresh, [int(cv.IMWRITE_JPEG_QUALITY), 100])

self.thresh_smooth()

cv.imwrite('smooth' + str(li) + '.jpg', self.smooth, [int(cv.IMWRITE_JPEG_QUALITY), 100])

self.smooth_lap()

cv.imwrite('lap' + str(li) + '.jpg', self.laplacian, [int(cv.IMWRITE_JPEG_QUALITY), 100])

self.create_sfr()

self.create_bdr()

if __name__ == '__main__':

pass

**dt_blueprints.py**

# -*- coding:UTF-8 -*-

# By

# data_pretreat

import numpy as np

from pg_base import *

from di_base import *

class Blueprintstreat:

def __init__(self, bps, sol, prc):

self.bps = bps

self.sol = sol

self.prc = prc

self.height = int(bps.height())

self.width = int(bps.width())

self.spraydots = int(prc.sprayRadius * bps.dpm)

self.routedots = int(bps.dpm * prc.routeSpacing)

self.upper_bound = int(0)

self.lower_bound = int(0)

self.layers_number = len(bps.bluePrints)

self.area_number = len(sol.std) - 1

self.gray = np.zeros((self.height, self.width, 3), np.uint8)

self.sampling = np.zeros((self.height, self.width, 3), np.uint8)

self.thresh = np.zeros((self.height, self.width, 3), np.uint8)

self.laplacian = np.zeros((self.height, self.width, 3), np.uint8)

self.border = np.zeros((self.height, self.width, 3), np.uint8)

self.sol_list = [[[] for _ in range(self.area_number)] for _ in range(self.layers_number)]

self.sfc_list = [[[] for _ in range(self.area_number)] for _ in range(self.layers_number)]

self.bdr_list = [[[] for _ in range(self.area_number)] for _ in range(self.layers_number)]

self.path_list = [[[] for _ in range(self.area_number)] for _ in range(self.layers_number)]

def idt_gray(self, i):

layer_i = cv.imread(self.bps.bluePrints[i-1])

for i in range(self.height):

for j in range(self.width):

value = layer_i[i, j][0] * 1000000 + layer_i[i, j][1] * 1000 + layer_i[i, j][2]

gray = int(value / 1001001)

self.gray[i, j] = np.uint8(gray)

def gray_samp(self):

sampdots_h = int(self.height / self.routedots)

sampdots_w = int(self.width / self.routedots)

for i in range(sampdots_h):

y = i * self.routedots

for j in range(sampdots_w):

x = j * self.routedots

g = self.gray[y, x]

for n in range(self.routedots):

for m in range(self.routedots):

self.sampling[y + n, x + m] = np.uint8(g)

def samp_thresh(self):

ret, self.thresh = cv.threshold(self.sampling, self.upper_bound, 255, cv.THRESH_TOZERO)

ret, self.thresh = cv.threshold(self.thresh, self.lower_bound, 255, cv.THRESH_TOZERO_INV)

ret, self.thresh = cv.threshold(self.thresh, self.upper_bound + 1, 255, cv.THRESH_BINARY_INV)

def thresh_lap(self):

dst = cv.Laplacian(self.thresh, cv.CV_16S, ksize=3)

self.laplacian = cv.convertScaleAbs(dst)

def show_border(self, nmb):

self.border = cv.imread(self.bps[nmb])

for y in range(self.height):

for x in range(self.width):

if self.laplacian[y, x][0] == 0 and self.laplacian[y, x][1] == 0 and self.laplacian[y, x][2] == 0:

pass

else:

self.border[y, x] = [0, 0, 255]

def create_sfr(self, m, n):

sfc = [[0 for _ in range(self.width)] for _ in range(self.height)]

for y in range(self.height):

for x in range(self.width):

sfc[y][x] = int(self.thresh[y, x][0] / 255 + epsilon)

self.sfc_list[m][n] = sfc

def create_bdr(self, m, n):

bdr = [[0 for _ in range(self.width)] for _ in range(self.height)]

for y in range(self.height):

for x in range(self.width):

bdr[y][x] = int(self.laplacian[y, x][0] / 255 + epsilon)

self.bdr_list[m][n] = bdr

def whole_treat(self):

for m in range(self.layers_number):

self.idt_gray(m)

cv.imwrite('gray.jpg', self.gray, [int(cv.IMWRITE_JPEG_QUALITY), 100])

self.gray_samp()

cv.imwrite('sampling.jpg', self.sampling, [int(cv.IMWRITE_JPEG_QUALITY), 100])

for n in range(self.area_number):

self.upper_bound = self.sol.std[n][0]

self.lower_bound = self.sol.std[n + 1][0]

self.samp_thresh()

cv.imwrite(str(m) + str(n) + 'thresh' + '.jpg', self.thresh, [int(cv.IMWRITE_JPEG_QUALITY), 100])

self.thresh_lap()

cv.imwrite(str(m) + str(n) + 'lap' + '.jpg', self.laplacian, [int(cv.IMWRITE_JPEG_QUALITY), 100])

self.sol_list[m][n] = self.sol.std[n]

self.create_sfr(m, n)

self.create_bdr(m, n)

def pre_process(self, m):

self.idt_gray(m)

cv.imwrite('gray.jpg', self.gray, [int(cv.IMWRITE_JPEG_QUALITY), 100])

self.gray_samp()

cv.imwrite('sampling.jpg', self.sampling, [int(cv.IMWRITE_JPEG_QUALITY), 100])

def post_process(self, n):

self.upper_bound = self.sol.std[n][0]

self.lower_bound = self.sol.std[n+1][0]

self.samp_thresh()

cv.imwrite(str(n) + 'thresh' + '.jpg', self.thresh, [int(cv.IMWRITE_JPEG_QUALITY), 100])

self.thresh_lap()

cv.imwrite(str(n) + 'lap' + '.jpg', self.laplacian, [int(cv.IMWRITE_JPEG_QUALITY), 100])

if __name__ == '__main__':

pass

**dt_character.py**

# -*- coding:UTF-8 -*-

# By

# data_treat

import numpy as np

from pg_base import *

from di_base import *

class Charactertreat:

def __init__(self, cha, idt, prc):

self.cha = cha

self.idt = idt #

self.prc = prc #

self.height = int(cha.height()) #

self.width = int(cha.width()) #

self.spraydots = int(prc.sprayRadius * cha.dpm) #

self.routedots = int(cha.dpm * prc.routeSpacing) #

self.upper_bound = int(130) #

self.lower_bound = int(150) #

self.area_number = len(idt.std) - 1

# 新建预处理中间文件

self.gray = np.zeros((self.height, self.width, 3), np.uint8)

self.filter = np.zeros((self.height, self.width, 3), np.uint8)

self.sampling = np.zeros((self.height, self.width, 3), np.uint8)

self.thresh = np.zeros((self.height, self.width, 3), np.uint8)

self.smooth = np.zeros((self.height, self.width, 3), np.uint8)

self.laplacian = np.zeros((self.height, self.width, 3), np.uint8)

self.border = np.zeros((self.height, self.width, 3), np.uint8)

#

self.sfc_list = []

self.bdr_list = []

def idt_gray(self): #

rw1 = self.idt.rw()

gw1 = self.idt.gw()

bw1 = self.idt.bw()

for i in range(self.height):

for j in range(self.width):

#

gray = 255 - (rw1 * self.cha.org[i, j][2] + gw1 * self.cha.org[i, j][1] + bw1 * self.cha.org[i, j][0])

log_gray = 255 - 45.9 * np.log(1.0 + gray)

self.gray[i, j] = np.uint8(log_gray)

def gray_filter(self): #

self.filter = cv.blur(self.gray, (int(self.spraydots / 2), int(self.spraydots / 2)))

def filter_samp(self): #

sampdots_h = int(self.height / self.routedots)

sampdots_w = int(self.width / self.routedots)

for i in range(sampdots_h):

#

y = i * self.routedots

for j in range(sampdots_w):

#

x = j * self.routedots

#

g = self.filter[y, x]

#

for n in range(self.routedots):

for m in range(self.routedots):

self.sampling[y + n, x + m] = np.uint8(g)

def samp_thresh(self): #

ret, self.thresh = cv.threshold(self.sampling, self.upper_bound, 255, cv.THRESH_TOZERO)

ret, self.thresh = cv.threshold(self.thresh, self.lower_bound, 255, cv.THRESH_TOZERO_INV)

ret, self.thresh = cv.threshold(self.thresh, self.upper_bound+1, 255, cv.THRESH_BINARY_INV)

def thresh_smooth(self): #

self.smooth = self.thresh.copy()

for m in range(5):

#

k_open = np.ones((m + self.routedots, m + self.routedots), np.uint8)

self.smooth = cv.morphologyEx(self.smooth, cv.MORPH_OPEN, k_open)

#

k_close = np.ones((m + self.routedots, m + self.routedots), np.uint8)

self.smooth = cv.morphologyEx(self.smooth, cv.MORPH_CLOSE, k_close)

def smooth_lap(self): #

dst = cv.Laplacian(self.smooth, cv.CV_16S, ksize=3)

self.laplacian = cv.convertScaleAbs(dst)

def show_border(self): #

self.border = self.cha.org.copy()

for y in range(self.height):

for x in range(self.width):

if self.laplacian[y, x][0] == 0 and self.laplacian[y, x][1] == 0 and self.laplacian[y, x][2] == 0:

pass

else:

self.border[y, x] = [0, 0, 255]

def create_sfr(self, m, n): #

sfc = [[0 for _ in range(self.width)] for _ in range(self.height)]

for y in range(self.height):

for x in range(self.width):

sfc[y][x] = int(self.smooth[y, x][0] / 255 + epsilon)

self.sfc_list[m][n] = sfc

def create_bdr(self, m, n): #

bdr = [[0 for _ in range(self.width)] for _ in range(self.height)]

for y in range(self.height):

for x in range(self.width):

bdr[y][x] = int(self.laplacian[y, x][0] / 255 + epsilon)

self.bdr_list[m][n] = bdr

def whole_treat(self): #

self.idt_gray()

cv.imwrite('gray.jpg', self.gray, [int(cv.IMWRITE_JPEG_QUALITY), 100])

self.gray_filter()

cv.imwrite('filter.jpg', self.filter, [int(cv.IMWRITE_JPEG_QUALITY), 100])

self.filter_samp()

cv.imwrite('sampling.jpg', self.sampling, [int(cv.IMWRITE_JPEG_QUALITY), 100])

for n in range(self.area_number):

self.upper_bound = self.idt.std[n][0]

self.lower_bound = self.idt.std[n + 1][0]

self.samp_thresh()

cv.imwrite(str(n) + 'thresh' + '.jpg', self.thresh, [int(cv.IMWRITE_JPEG_QUALITY), 100])

self.thresh_smooth()

cv.imwrite(str(n) + 'smooth' + '.jpg', self.smooth, [int(cv.IMWRITE_JPEG_QUALITY), 100])

self.smooth_lap()

cv.imwrite(str(n) + 'lap' + '.jpg', self.laplacian, [int(cv.IMWRITE_JPEG_QUALITY), 100])

self.create_sfr(0, n)

self.create_bdr(0, n)

def pre_process(self): #

self.idt_gray()

cv.imwrite('gray.jpg', self.gray, [int(cv.IMWRITE_JPEG_QUALITY), 100])

self.gray_filter()

cv.imwrite('filter.jpg', self.filter, [int(cv.IMWRITE_JPEG_QUALITY), 100])

self.filter_samp()

cv.imwrite('sampling.jpg', self.sampling, [int(cv.IMWRITE_JPEG_QUALITY), 100])

def post_process(self, n):

self.upper_bound = self.idt.std[n][0]

self.lower_bound = self.idt.std[n+1][0]

self.samp_thresh()

cv.imwrite(str(n) + 'thresh' + '.jpg', self.thresh, [int(cv.IMWRITE_JPEG_QUALITY), 100])

self.thresh_smooth()

cv.imwrite(str(n) + 'smooth' + '.jpg', self.smooth, [int(cv.IMWRITE_JPEG_QUALITY), 100])

self.smooth_lap()

cv.imwrite(str(n) + 'lap' + '.jpg', self.laplacian, [int(cv.IMWRITE_JPEG_QUALITY), 100])

self.create_sfr(0, n)

self.create_bdr(0, n)

if __name__ == '__main__':

character = Character('exp', 'm2.png', 16)

indicator = Indicator((255, 255, 0), 100)

process = Process()

pretreated = Charactertreat(character, indicator, process)

# pretreated.pre_process()

# pretreated.post_process()

pretreated.whole_treat()

# pretreated = dataPretreat(character, indicator, process)

# border = pretreated[1]

# print(border)

# sm = pretreated[0]

# cv.imwrite('sm.jpg', sm, [int(cv.IMWRITE_JPEG_QUALITY), 100])

**bs_base.py**

# -*- coding:UTF-8 -*-

# By

# border_splicing

from pg_algo import *

from di_base import *

class LinkPoint: #

def __init__(self, pnt2d): #

self.x = pnt2d.x

self.y = pnt2d.y

self.other = None

self.used = False

self.index = 0

def __str__(self):

return 'LinkPoint: used: %s; self: %s, %s; other: %s, %s; link to: %s' \

% (self.used, self.x, self.y, self.other.x, self.other.y, self.other.index)

def to_point2d(self): #

return Point2D(self.x, self.y)

if __name__ == '__main__':

# character = Character('exp', 'm5.png', 16)

# indicator = Indicator((0, 255, 255), 100)

# process = Process()

# pretreat = dp.dataPretreat(character, indicator, process)

# crtvtx = create_vertex(character, pretreat)

# border = crtvtx[1]

# vertex = crtvtx[2]

# l = len(vertex)

# for m in range(l):

# p1x = int(vertex[m].x)

# p1y = int(vertex[m].y)

# print(p1x, p1y)

#

# vertex_point = np.zeros((character.height(), character.width(), 3), np.uint8)

# for m in range(l):

# p1x = int(vertex[m].x)

# p1y = int(vertex[m].y)

# vertex_point[p1y, p1x] = [255, 255, 255]

# cv.imwrite('vertex_test.jpg', vertex_point, [int(cv.IMWRITE_JPEG_QUALITY), 100])

#

# edge = create_edge(border, vertex)

# print(edge)

# routedots = int(character.dpm * process.routeSpacing)

# for n in range(5):

# p2x = int(vertex[n].x)

# p2y = int(vertex[n].y)

# print(p2x)

# print(p2y)

# pmx = int((p1x + p2x + routedots / 2) / 2)

# pmy = int((p1y + p2y + routedots / 2) / 2)

# mdl = border[pmy][pmx]

# if (p1x != p2x or p1y != p2y) and mdl != 0:

# print(pnt1)

# print(pnt2)

pass

**bs_vertex.py**

# -*- coding:UTF-8 -*-

# By

# border_splicing

from dt_character import *

from dt_blueprints import *

from bs_base import *

class Vertexes:

def __init__(self, sfc, bdr): #

self.sfc = sfc

self.bdr = bdr

self.vtx = []

self.ra = []

self.crs1 = []

self.crs2 = []

def classify_vertex(self):

height = len(self.bdr)

width = len(self.bdr[0])

for y in range(2, height - 2, 1):

for x in range(2, width - 2, 1):

if self.bdr[y][x] == 1:

jy1 = self.bdr[y - 1][x] * self.bdr[y + 1][x]

jx1 = self.bdr[y][x - 1] * self.bdr[y][x + 1]

jy2 = self.bdr[y - 2][x] * self.bdr[y + 2][x]

jx2 = self.bdr[y][x - 2] * self.bdr[y][x + 2]

if jy1 == jx1 == 1 and jy2 == jx2 == 0:

pnt2d = Point2D(int(x), int(y), 2)

self.ra.append(pnt2d)

elif jy1 == jx1 == jy2 == jx2 == 1 and self.bdr[y - 1][x - 1] == 0:

if self.sfc[y - 1][x - 1] == 0:

pnt3 = Point2D(int(x), int(y), 3)

self.crs1.append(pnt3)

elif self.sfc[y - 1][x - 1] == 1:

pnt4 = Point2D(int(x), int(y), 4)

self.crs2.append(pnt4)

def collect_vertex(self):

lra = len(self.ra)

lc1 = len(self.crs1)

lc2 = len(self.crs2)

for ri in range(lra):

prx = int(self.ra[ri].x)

pry = int(self.ra[ri].y)

pntra = Point2D(int(prx), int(pry), 2)

self.vtx.append(pntra)

for c1i in range(lc1):

pc1x = int(self.crs1[c1i].x)

pc1y = int(self.crs1[c1i].y)

pntc11 = Point2D(int(pc1x), int(pc1y), 2)

pntc12 = Point2D(int(pc1x + 1), int(pc1y + 1), 2)

self.vtx.append(pntc11)

self.vtx.append(pntc12)

for c2i in range(lc2):

pc2x = int(self.crs2[c2i].x)

pc2y = int(self.crs2[c2i].y)

pntc21 = Point2D(int(pc2x + 1), int(pc2y), 2)

pntc22 = Point2D(int(pc2x), int(pc2y + 1), 2)

self.vtx.append(pntc21)

self.vtx.append(pntc22)

def border_modify(self):

lc1 = len(self.crs1)

for c1i in range(lc1):

p3x = int(self.crs1[c1i].x)

p3y = int(self.crs1[c1i].y)

self.bdr[p3y + 1][p3x] = 0

self.bdr[p3y][p3x + 1] = 0

self.bdr[p3y - 1][p3x - 1] = 1

self.bdr[p3y + 2][p3x + 2] = 1

lc2 = len(self.crs2)

for c2i in range(lc2):

p4x = int(self.crs2[c2i].x)

p4y = int(self.crs2[c2i].y)

self.bdr[p4y][p4x] = 0

self.bdr[p4y + 1][p4x + 1] = 0

self.bdr[p4y + 2][p4x - 1] = 1

self.bdr[p4y - 1][p4x + 2] = 1

def create_vertexes(self):

self.classify_vertex()

self.collect_vertex()

self.border_modify()

if __name__ == '__main__':

pass

**bs_linkvertex.py**

# -*- coding:UTF-8 -*-

# By

# border_splicing

from bs_vertex import *

class Linkvertex:

def __init__(self, bdr, vtx):

self.bdr = bdr

self.vtx = vtx

self.dic = {}

self.contours = []

self.polys = []

self.segs = []

def add_pnt_dic(self, pnt1, pnt2):

lp1, lp2 = LinkPoint(pnt1), LinkPoint(pnt2)

lp1.other = lp2

lp2.other = lp1

if (lp1.x, lp1.y) not in self.dic.keys():

self.dic[(lp1.x, lp1.y)] = []

self.dic[(lp1.x, lp1.y)].append(lp1)

pnt1.w -= 1

if (lp2.x, lp2.y) not in self.dic.keys():

self.dic[(lp2.x, lp2.y)] = []

self.dic[(lp2.x, lp2.y)].append(lp2)

pnt2.w -= 1

def is_pnts_link(self, pnt1, pnt2):

dtx = int(abs(pnt2.x - pnt1.x))

dty = int(abs(pnt2.y - pnt1.y))

midpnt = 1

link = False

if (dtx == 0 and dty == 0) or pnt1.w == 0 or pnt2.w == 0:

pass

elif dty <= 1:

xmax = max(pnt1.x, pnt2.x)

xmin = min(pnt1.x, pnt2.x)

for p in range(xmin + 1, xmax, 1):

midpnt *= self.bdr[pnt1.y][p]

if midpnt == 0:

pass

else:

link = True

elif dtx <= 1:

ymax = max(pnt1.y, pnt2.y)

ymin = min(pnt1.y, pnt2.y)

for q in range(ymin + 1, ymax, 1):

midpnt *= self.bdr[q][pnt1.x]

if midpnt == 0:

pass

else:

link = True

else:

pass

return link

def create_lp_dic(self):

lov = len(self.vtx)

for m in range(lov):

pnt1 = self.vtx[m]

for n in range(lov):

pnt2 = self.vtx[n]

if self.is_pnts_link(pnt1, pnt2):

self.add_pnt_dic(pnt1, pnt2)

else:

pass

def print_lp_dic(self):

nmb = 1

for pnt in self.vtx:

px = pnt.x

py = pnt.y

print(nmb, ':')

print(pnt)

lkpnts = self.dic[(px, py)]

ll = len(lkpnts)

for li in range(ll):

lkpnt = lkpnts[li]

print(lkpnt)

nmb += 1

def find_unused_pnt(self):

for pnts in self.dic.values():

for pnt in pnts:

if pnt.used is False:

return pnt

return None

def find_next_pnt(self, pnt):

other = pnt.other

pnts = self.dic[(other.x, other.y)]

next_pnt = None

for pntn in pnts:

if pntn.other.x != pnt.x or pntn.other.y != pnt.y:

next_pnt = pntn

else:

pass

return next_pnt

def link_vertex(self):

self.create_lp_dic()

while True:

vp = self.find_unused_pnt()

if vp is None:

break

poly = Polyline()

while True:

poly.add_point(vp.to_point2d())

vp.used = True

vp.other.used = True

vp = self.find_next_pnt(vp)

if poly.is_close():

self.contours.append(poly)

break

if vp is None:

self.polys.append(poly)

break

def polys_to_segs(self):

pass

if __name__ == '__main__':

pass

**cl_adaptor.py**

# -*- coding:UTF-8 -*-

# By

# clipper

from pyclipper import *

from pg_polyline import *

class ClipperAdaptor:

def __init__(self, digits=7):

self.f = math.pow(10, digits)

self.arcTolerance = 0.005

def to_path(self, poly):

path = []

for pt in poly.points:

path.append((pt.x * self.f, pt.y * self.f))

return path

def to_paths(self, polys):

paths = []

for poly in polys:

paths.append(self.to_path(poly))

return paths

def to_poly(self, path, closed=True):

poly = Polyline()

for tp in path:

poly.add_point(Point2D(tp[0]/self.f, tp[1]/self.f))

if len(path) > 0 and closed:

poly.add_point(poly.start_point())

return poly

def to_polys(self, paths, closed=True):

polys = []

for path in paths:

polys.append(self.to_poly(path, closed))

return polys

if __name__ == '__main__':

pass

**pp_base.py**

# -*- coding:UTF-8 -*-

# By

# path_planning

from pg_algo import *

class SweepLine:

def __init__(self):

self.segs = []

def sweepline_intersect(self, y):

ips = [] # 交点列表

y_line = Line(Point2D(0, y), Vector2D(1, 0, 0))

for seg in self.segs:

if seg.pot1.y == y:

ips.append(seg.pot1.clone())

elif seg.pot2.y == y:

ips.append(seg.pot2.clone())

else:

ip = intersect(y_line, seg)

if ip is not None:

ips.append(ip)

ips.sort(key=lambda p: p.x)

i = len(ips) - 1

while i > 0:

if ips[i].distance(ips[i - 1]) == 0:

del ips[i]

del ips[i-1]

i = i - 2

else:

i = i - 1

return ips

def calc_path_points(polygons, ys):

segs = []

for poly in polygons:

for i in range(poly.count() - 1):

seg = Segment(poly.point(i), poly.point(i + 1))

segs.append(seg)

segs.sort(key=lambda s: s.y_min())

k = 0

sweep = SweepLine()

ipses = []

for y in ys:

for i in range(len(sweep.segs) - 1, -1, -1):

if sweep.segs[i].y_max() < y:

del sweep.segs[i]

for i in range(k, len(segs)):

if segs[i].y_min() < y and segs[i].y_max() >= y:

sweep.segs.append(segs[i])

elif segs[i].y_min() >= y:

k = i

break

if len(sweep.segs) > 0:

ips = sweep.sweepline_intersect(y)

ipses.append(ips)

return ipses

def gen_sweep_hatches(polygons, interval, angle):

mt, mb = Matrix2D.create_rotate_matrix('Z', -angle), Matrix2D.create_rotate_matrix('Z', angle)

rot_polys = []

for poly in polygons:

rot_polys.append(poly.multplied(mt))

ymin, ymax = float('inf'), float('-inf')

for poly in rot_polys:

for pt in poly.points:

ymin, ymax = min(ymin, pt.y), max(ymin, pt.y)

ys = []

y = ymin + interval

while y < ymax:

ys.append(y)

y += interval

segs = gen_hatch(rot_polys, ys)

for seg in segs:

seg.multiply(mb)

return segs

def gen_hatch(polygons, ys):

segs = []

ipses = calc_path_points(polygons, ys)

for ips in ipses:

for i in range(0, len(ips), 2):

segs.append(Segment(ips[i], ips[i+1]))

return segs

**pp_genpath.py**

# -*- coding:UTF-8 -*-

# By

# path_planning

from pp_splitregion import *

from bs_linkvertex import *

class GenPath:

def __init__(self, polygons, interval, angle):

self.polygons = polygons

self.interval = interval

self.angle = angle

self.splitPolys = []

def generate(self):

rot_polys = rotatePolygons(self.polygons, -self.angle)

ys = self.gen_scan_ys(rot_polys)

self.splitPolys = split_region(rot_polys)

paths = []

for poly in self.splitPolys:

segs = gen_hatch([poly], ys)

if len(segs) > 0:

path = self.link_local_hatch(segs)

paths.append(path)

return rotatePolygons(paths, self.angle)

def generate_angle_zero(self):

org_polys = self.polygons

ys = self.gen_scan_ys(org_polys)

self.splitPolys = split_region(org_polys)

paths = []

for poly in self.splitPolys:

segs = gen_hatch([poly], ys)

if len(segs) > 0:

path = self.link_local_hatch(segs)

paths.append(path)

return paths

def gen_scan_ys(self, polygons):

ys = []

ymin, ymax = float('inf'), float('-inf')

for poly in polygons:

for pt in poly.points:

ymin, ymax = min(ymin, pt.y), max(ymax, pt.y)

y = ymin + self.interval/2

while y < ymax:

ys.append(y)

y += self.interval

return ys

def link_local_hatch(self, segs):

poly = Polyline()

for i, seg in enumerate(segs):

poly.add_point(seg.pot1 if (i % 2 == 0) else seg.pot2)

poly.add_point(seg.pot2 if (i % 2 == 0) else seg.pot1)

return poly

if __name__ == '__main__':

pass

**pp_splitregion.py**

# -*- coding:UTF-8 -*-

# By

# path_planning

from pp_base import *

from cl_adaptor import *

class SplitRegion:

def __init__(self, polygons, adjust_poly_dirs=False):

self.polygons = polygons

if adjust_poly_dirs:

adjust_polygon_dirs(self.polygons)

self.splitPolygons = self.split()

def split(self):

turn_pts = self.find_turn_point()

if len(turn_pts) != 0:

ys = []

for pt in turn_pts:

ys.append(pt.y)

ys.sort()

route_ptses = calc_path_points(self.polygons, ys)

splitters = []

for turn_pt in turn_pts:

lpt, rpt = self.find_lr_points(turn_pt, route_ptses)

if lpt is not None and rpt is not None:

splitter = self.create_splitter(lpt, rpt)

splitters.append(splitter)

if len(splitters) != 0:

clipper, ca = Pyclipper(), ClipperAdaptor()

clipper.AddPaths(ca.to_paths(self.polygons), PT_SUBJECT)

clipper.AddPaths(ca.to_paths(splitters), PT_CLIP)

sln = clipper.Execute(CT_DIFFERENCE)

return ca.to_polys(sln)

return self.polygons

def find_turn_point(self):

vx = Vector2D(1, 0, 0)

turn_pts = []

for poly in self.polygons:

for i in range(poly.count()-1):

pts = poly.points

v1 = pts[-2 if (i == 0) else (i - 1)].point_to(pts[i])

v2 = pts[i].point_to(pts[i + 1])

if v1.cross_product(vx) * v2.cross_product(vx) <= 0:

if v1.cross_product(v2) <= 0:

turn_pts.append(pts[i])

return turn_pts

def find_lr_points(self, pt, ptses):

for pts in ptses:

if len(pts) > 0 and pts[0].y == pt.y:

for i in range(len(pts) - 1):

if pt.x > pts[i].x and pt.x < pts[i+1].x:

return pts[i], pts[i+1]

return None, None

def create_splitter(self, p1, p2, delta=1.0e-5):

vx, vy = Vector2D(1, 0, 0), Vector2D(0, 1, 0)

splitter = Polyline()

splitter.add_point(p1 - vx.amplified(delta) - vy.amplified(delta))

splitter.add_point(p2 + vx.amplified(delta) - vy.amplified(delta))

splitter.add_point(p2 + vx.amplified(delta) + vy.amplified(delta))

splitter.add_point(p1 - vx.amplified(delta) + vy.amplified(delta))

splitter.add_point(splitter.start_point())

return splitter

def split_region(polygons, adjust_poly_dirs=False):

return SplitRegion(polygons, adjust_poly_dirs).splitPolygons
